# Non‐PFAS‐Based Magnetic Polymer Sorbents for Efficient Removal of Perfluorinated Compounds from Landfill Leachate

**DOI:** 10.1002/adma.202502427

**Published:** 2025-09-15

**Authors:** Xiao Tan, Zhuojing Yang, Zhou Chen, Yutong Zhu, Chunrong Yu, Yiqing Wang, Kehan Liu, Pradeep Dewapriya, Xuemei Li, Zicheng Su, Marcus J. Giansiracusa, Colette Boskovic, Biao Wang, Kevin V. Thomas, Cheng Zhang

**Affiliations:** ^1^ Australian Institute for Bioengineering and Nanotechnology The University of Queensland Brisbane Queensland 4072 Australia; ^2^ Centre for Advanced Imaging The University of Queensland Brisbane Queensland 4072 Australia; ^3^ Queensland Alliance for Environmental Health Sciences (QAEHS) The University of Queensland Woolloongabba Queensland 4102 Australia; ^4^ School of Chemistry University of Melbourne Parkville Victoria 3010 Australia

**Keywords:** PFAS remediation, magnetic fluorinated polymer sorbents, magnetic separation, landfill leachate, regeneration and reusability

## Abstract

Per‐ and polyfluoroalkyl substances (PFAS) are carcinogenic and environmentally persistent contaminants, necessitating their efficient removal to protect environmental and human health. In this study, a series of fluorinated, non‐PFAS‐based magnetic polymer sorbents is developed for the selective removal of multiple PFAS from landfill leachate containing high levels of co‐contaminants. Crosslinked polymer networks are shown to enhance both magnetic separation and PFAS sorption performance. The magnetic sorbent with a higher proportion of quaternary ammonium groups and a lower proportion of fluorinated segments shows improved sorption kinetics for most PFAS and greater sorption capacity for perfluorobutanesulfonic acid (PFBS), the most abundant compound in the tested landfill leachate. However, this formulation exhibits lower sorption selectivity and reduced equilibrium removal efficiencies when PFAS are present at low initial concentrations. A compact and portable treatment device is constructed to simulate practical deployment. The system achieves high PFAS removal efficiencies (>90% for the majority of PFAS) and maintains good regeneration and reusability over five sorption‐desorption cycles at environmentally relevant concentrations. Compared to four commercially available sorbents, the developed magnetic polymer sorbents exhibit superior performance in PFAS removal, indicating their potential as efficient candidates for remediating PFAS from landfill leachate.

## Graphical abstract

Magnetic polymer sorbents featuring non‐per‐ and polyfluoroalkyl substances (non‐PFAS)‐based fluorinated and cationic groups enable selective and efficient removal of multiple PFAS from complex landfill leachate. By optimizing functional group balance and polymer design, the sorbents achieve >90% removal, excellent reusability, and outperform commercial materials—offering a promising solution for PFAS remediation in challenging real‐world water matrices.

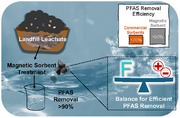

## 1 Introduction

Per‐ and polyfluoroalkyl substances (PFAS) are a class of synthetic chemicals widely used in the manufacturing industry and household products, such as non‐stick cookware, food packaging, and firefighting foam.^[^ ^1^, ^2^ ^]^ Due to the presence of carbon‐fluorine bonds, PFAS are highly stable and resistant to heat, water, and oil, and are often referred to as “forever chemicals”.^[^ ^3^ ^]^ Their extensive use has led to widespread environmental contamination, especially in water sources, where they accumulate over time and can pose significant health risks, including liver damage,^[^ ^4^ ^]^ immune system suppression,^[^ ^5^ ^]^ reproductive health issues,^[^ ^6^ ^]^ and cancers.^[^ ^7^ ^]^

Landfill leachate is a liquid formed when water or moisture percolates through waste materials in a landfill, picking up various contaminants along the way.^[^ ^8^ ^]^ It typically contains a mixture of organic and inorganic compounds, including heavy metals, organic compounds, and increasingly, PFAS.^[^ ^9^, ^10^ ^]^ PFAS contamination in landfill leachate poses significant environmental and health risks. However, the efficient removal of PFAS from landfill leachate is challenging due to the interference from other co‐contaminants. Traditional PFAS sorption technologies, such as granular activated carbon (GAC) and ion‐exchange (IEX) resins,^[^ ^11^ ^]^ have several limitations when treating landfill leachate. GAC removes PFAS primarily through hydrophobic interactions and is more effective for removing PFAS with longer chain lengths than those with shorter chain lengths.^[^ ^12^ ^]^ In addition, the difficulty in regenerating GAC makes it expensive and challenging to replace or regenerate the carbon media.^[^ ^13^, ^14^ ^]^ IEX resins can remove different types of PFAS through a combination of hydrophobic and electrostatic interactions,^[^ ^15^, ^16^, ^17^ ^]^ with the additional benefit of being regenerable. However, the presence of high concentrations of other co‐contaminants in landfill leachate competes for active sorption sites on resins,^[^ ^18^, ^19^ ^]^ significantly reducing the efficiency of PFAS removal and thus limiting the feasibility of using such sorbents in practical applications.

Fluorinated polymer sorbents are emerging as effective materials for the rapid, efficient, and selective removal of PFAS from various contaminated sources.^[^ ^20^, ^21^, ^22^, ^23^, ^24^, ^25^, ^26^, ^27^, ^28^, ^29^ ^]^ The key to their success lies in the inclusion of fluorous interactions, which selectively capture PFAS in aqueous solutions by exploiting fluorophilicity. For example, Koda et al. from the Sawamoto group developed fluorous‐core star polymers consisting of a polyfluorinated microgel core and hydrophilic poly(ethylene glycol) methyl ether (PEG)‐functionalized arms, synthesized via ruthenium‐catalyzed living radical polymerization.^[^ ^20^ ^]^ Using the dialysis method, these star polymers demonstrated 97.5% removal of perfluorooctanoic acid (PFOA) from water at an initial PFAS concentration of 10 parts per million (ppm). By incorporating 2‐(dimethylamino)ethyl methacrylate (DMAEMA) and crosslinking the star polymers,^[^ ^21^ ^]^ the same research group prepared fluorine/amine‐functionalized star polymer gels that removed perfluorohexanoic acid (PFHxA) at an initial concentration of 10 ppm, achieving up to 91% removal through simple mixing and filtration. Taking the extra advantage of acid/base interaction between the in‐core amine of the gel and the carboxylic acid functional group of the PFAS, the authors observed a higher removal efficiency on PFHxA compared with the fluorous gel in the absence of DMAEMA, suggesting a synergistic effect of both fluorous and acid/base interactions in PFAS removal. Building on this concept, Quan et al. produced fluorous‐core nanoparticle‐embedded hydrogels via metal‐free tandem photocontrolled radical polymerization.^[^ ^22^ ^]^ These fluorous gels exhibited improved chemical stability due to the replacement of ester functional groups with methacrylamides. After treating aqueous solutions containing PFOA at initial concentrations ranging from 1 to 1000 parts per billion (ppb) for 6 h and filtration, 99.08%–99.999% removal of PFOA was achieved. These results demonstrate the potential of such fluorinated sorbents for PFAS removal at environmentally relevant concentrations. Kumarasamy and co‐workers extended this concept through a more comprehensive study,^[^ ^23^ ^]^ by developing ionic fluorogels using perfluoropolyether (PFPE) as the fluorophilic matrix material, along with quaternized ammonium functional groups. The prepared ionic fluorogels demonstrated >80% removal of 18 legacy and emerging PFAS at an initial concentration of 1 ppb each from settled water collected at a water treatment plant within 2 h. More recently, our group reported thermo‐responsive quaternized PFPE‐containing block copolymers,^[^ ^26^ ^]^ synthesized via reversible addition‐fragmentation chain transfer polymerization, as efficient PFAS sorbents. By leveraging the thermo‐sensitive property of *N*‐isopropylacrylamide, the polymer demonstrated >99% removal of multiple PFAS through sorption, heating, and filtration.

An important consideration when designing PFAS sorbents is the separation of the sorbents from the purified solution after water treatment. The above‐mentioned studies typically employed techniques such as dialysis, filtration, or centrifugation to separate sorbent‐PFAS mixtures following complete sorption. To achieve a simpler separation process, magnetic polymer sorbents have been explored.^[^ ^24^, ^30^, ^31^, ^32^, ^33^ ^]^ We have prepared a series of PFPE‐containing magnetic sorbents that demonstrate efficient PFAS removal at an environmentally relevant concentration (1 ppb) from ground wastewaters.^[^ ^24^ ^]^ Nevertheless, several inherent limitations persist that constrain the practical application of such sorbents in water treatment, including 1) the use of PFPE, a type of PFAS,^[^ ^34^, ^35^ ^]^ may leach to the environment and cause secondary pollution, 2) the dynamic nature of the coordination bond formed through ligand exchange between the polymer and magnetic iron oxide nanoparticles (IONPs) is not stable in dirty PFAS‐contaminated water sources, such as landfill leachate,^[^ ^36^, ^37^ ^]^ and 3) the presence of large amounts of ester functional groups reduces the stability of the sorbents, particularly under basic conditions or elevated temperatures.^[^ ^38^, ^39^ ^]^

This study addresses the above limitations by design and preparation of a new type of regenerable magnetic polymer sorbent for efficient PFAS removal from dirty landfill leachate. The magnetic sorbent was prepared by grafting IONPs onto crosslinked polymer matrices synthesized using free radical polymerization, followed by quaternization to impart a permanent positive charge. Replacing PFAS segments of PFPE with 2,3,4,5,6‐pentafluorostyrene (PFS), a fluorinated but non‐PFAS monomer,^[^ ^34^ ^]^ facilitates selective PFAS capture through fluorous interactions but reduces environmental concerns. The substitution of esters with aromatic groups and the formation of covalent Si‐O‐Fe bonds improve the chemical stability of the sorbent.^[^ ^36^, ^37^, ^38^, ^39^, ^40^, ^41^ ^]^ The new sorbent exhibits superior performance in PFAS removal efficiency, capacity, and magnetic separation for regeneration. The balance between quaternary ammonium groups and PFS segments was explored, as it is essential for enhancing PFAS capture. A compact, portable device enables a continuous removal of >90% of the majority of PFAS at environmentally relevant concentrations and demonstrates excellent sorbent regeneration across five sorption‐desorption cycles. The superior performance of the new sorbent, compared to commercial alternatives, underscores its potential for efficient PFAS remediation in landfill leachate.

## 2 Results and Discussion

### 2.1 Design and Preparation of Magnetic PFS‐ and Styrene‐Containing Polymer Sorbents

**Figure 1:**
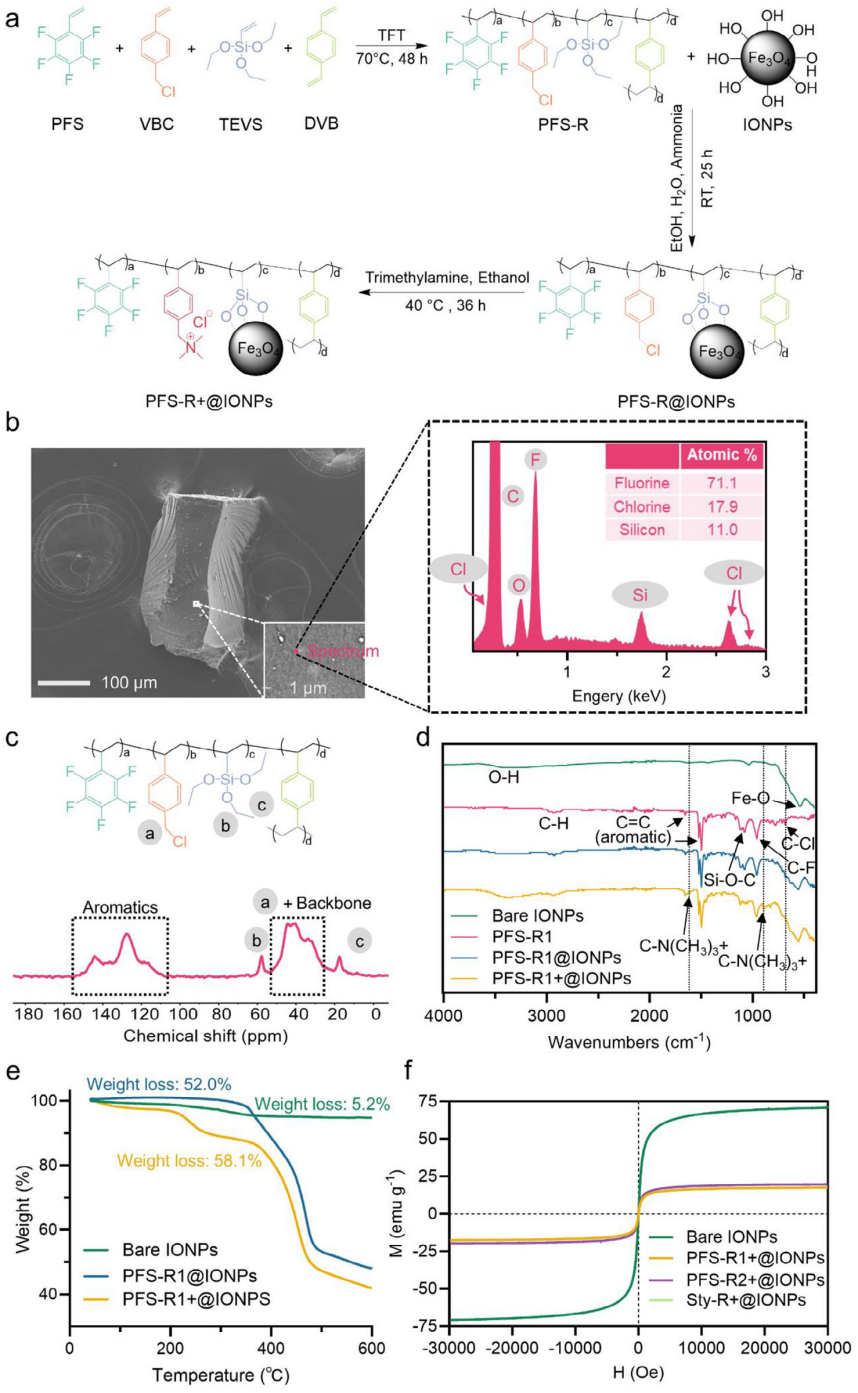
Characterizations of the new magnetic sorbents. a) Typical synthetic routine for PFS‐R+@IONPs. b) SEM and EDS spectra of PFS‐R1. c) ^13^C SS NMR of PFS‐R1. d) FT‐IR spectra of bare IONPs, PFS‐R1 before and after grafting with IONPs, and PFS‐R1+@IONPs. e) TGA of bare IONPs before and after grafting with PFS‐R1, and PFS‐R1+@IONPs. f) Magnetic hysteresis loops of bare IONPS and three crosslinked magnetic polymer sorbents after quaternization.

Before preparing the new magnetic polymer sorbents, four polymer matrices were first synthesized using free radical polymerization, including a PFS‐containing linear polymer, two PFS‐containing crosslinked resins, and a styrene‐containing crosslinked resin, namely PFS‐LP, PFS‐R1, PFS‐R2, and Sty‐R, respectively. Feeding ratios of each monomer in both weight and mole are summarized in Table S1 (Supporting Information). PFS‐LP, PFS‐R1, and Sty‐R have similar weight percentages of each monomer, while PFS‐R2 has a reversed percentage between 4‐vinylbenzyl chloride (VBC) and PFS compared with PFS‐R1. The overall synthetic scheme for preparing the magnetic polymeric sorbents is illustrated in **Figure** **1**a; Schemes S1, and S2 (Supporting Information).

The successful synthesis of PFS‐LP was confirmed by ^1^H and ^19^F NMR spectroscopy. ^1^H NMR (Figure S1a, Supporting Information) demonstrates that the conversions of VBC and triethoxyvinylsilane (TEVS) were calculated to be ≈100% and 21.9%, respectively, while the conversion of PFS was determined to be 97.7% by ^19^F NMR (Figure S1b, Supporting Information). After purification, both ^1^H and ^19^F NMR spectra in Figure S2 (Supporting Information) demonstrate the successful synthesis of the linear polymer.

PFS‐R1, PFS‐R2, and Sty‐R contain crosslinked networks and are not soluble in any deuterated solvent. Therefore, scanning electron microscopy‐energy dispersive spectroscopy (SEM‐EDS), ^13^C solid‐state (SS) NMR, and Fourier transform infrared spectroscopy (FT‐IR) were employed to characterize the three crosslinked resins. SEM‐EDS results (Figure 1b) show that the atomic percentages of F, Cl, and Si in PFS‐R1 after purification are 71.1%, 17.9%, and 11%, respectively, indicating the presence of all three functional groups from the feed. As expected, PFS‐R2 exhibits a significantly lower content of PFS and a much higher content of VBC, as reflected in the atomic percentages of F, Cl, and Si, measured at 61.1%, 31.9%, and 7.1%, respectively (Figure S3a, Supporting Information). A higher VBC content suggests a potentially greater degree of quaternization in PFS‐R2 compared to PFS‐R1 following the quaternization process. Due to the absence of a fluorinated functional group, Sty‐R shows atomic percentages of 71.5% for Cl and 28.5% for Si (Figure S4a, Supporting Information). ^13^C SS NMR spectra (Figure 1c; Figures S3b, and S4b, Supporting Information) further confirm the successful preparation of PFS‐R1, PFS‐R2, and Sty‐R. Broad overlapped peaks observed between 107.3 and 155.2 ppm are attributed to the carbons from aromatics, while the other set of broad peaks from 20.4 to 54.1 ppm corresponds to those carbons adjacent to chlorine (peak a) and those in the resin backbone.^[^ ^42^ ^]^ The presence of TEVS can be confirmed by two distinct peaks at 57.8 ppm (peak b) and 17.5 ppm (peak c), which are assigned to the methylene and methyl carbons of TEVS, respectively.^[^ ^43^ ^]^ FT‐IR results for the three crosslinked resins (Figure 1d (pink); Figures S6a and S7a, Supporting Information) also demonstrate their successful preparation. Taking PFS‐R1 as an example, peaks at 683 and 961 cm^−1^ correspond to the stretching vibrations of C‐Cl and C‐F, respectively,^[^ ^44^, ^45^ ^]^ and peaks at 1082 and 1116 cm^−1^ are attributed to Si‐O‐C stretching vibrations,^[^ ^46^ ^]^ indicating the presence of VBC, PFS, and TEVS functional groups. Compared to the spectrum of PFS‐R2, the peak at 683 cm^−1^ for PFS‐R1 shows significantly lower intensity, due to its lower content of VBC, in line with the observation from SEM‐EDS mentioned above.

Magnetic Fe_3_O_4_ IONPs bearing surface hydroxyl groups were synthesized via the co‐precipitation method. The FT‐IR spectrum (Figure 1d, green) shows a characteristic peak at 541 cm^−1^, typically attributed to Fe‐O stretching,^[^ ^47^ ^]^ confirming the successful formation of Fe_3_O_4_. The broad absorption band at ≈3400 cm^−1^ indicates the presence of hydroxyl groups on the IONPs surface.

All four polymer materials, along with magnetic IONPs, were ground into powder. Hydrolysis and condensation reactions further allow the grafting of IONPs onto polymer materials to produce PFS‐LP@IONPs, PFS‐R1@IONPs, PFS‐R2@IONPs, and Sty‐R@IONPs, as confirmed by FT‐IR. Typically, the FT‐IR spectrum for PFS‐R1@IONPs (Figure 1d, blue) shows peaks belonging to both PFS‐R1 and magnetic IONPs as discussed above, confirming the successful grafting of IONP powder onto the PFS‐R1 resin. Similar results were obtained for the other three magnetic polymer materials, as shown in Figures S5a,S6a, and S7a (Supporting Information). The grafting efficiency for each polymer material was measured using thermogravimetric analysis (TGA), showing 50%, 52%, 49.7%, and 68.1% for PFS‐LP@IONPs, PFS‐R1@IONPs, PFS‐R2@IONPs, and Sty‐R@IONPs, respectively (Figure 1e (blue); Figures S5b–S7b, Supporting Information).

Quaternization of VBC using trimethylamine was then conducted to produce PFS‐LP+@IONPs, PFS‐R1+@IONPs, PFS‐R2+@IONPs, and Sty‐R+@IONPs, as confirmed by FT‐IR, TGA, and light microscope (Figure 1d (yellow); Figures S5–S8, Supporting Information). For example, the disappearance of the peak at 683 cm^−1^ (attributed to C‐Cl stretching), and the formation of new peaks at 857 and 892 cm^−1^ due to the bending vibrations of the trimethyl group from quaternary ammonium cations,^[^ ^48^ ^]^ demonstrate the successful conversion of VBC to its quaternized form. Similarly, after quaternization, TGA results show an increase in weight loss upon heating, from 52.0% to 58.1% for PFS‐R1+@IONPs, 49.7% to 64.1% for PFS‐R2+@IONPs, and 68.1% to 76.7% for Sty‐R+@IONPs. However, a significant decrease in weight loss was observed for PFS‐LP+@IONPs after quaternization (from 50.0% to 14.5%). This may be attributed to the inefficient magnetic recovery of PFS‐LP+@IONPs, as the aqueous solution remained pale yellow after magnetic separation using a neodymium magnet (N50) for 10 min (Figure S9a, Supporting Information). The high water solubility and strong electrostatic repulsion between PFS‐LP+@IONPs particles (>50 mV, Figure S9b, Supporting Information) result in such low recovery via magnetic separation. Finally, rapid magnetic recovery of the new crosslinked magnetic sorbents can be achieved within 2 min in water (Figure S10, Supporting Information).

The magnetic properties of the three crosslinked sorbents were investigated via magnetic hysteresis measurements. As shown in Figure 1f, the bare IONPs exhibit a saturation magnetization of 70.9 emu g^−1^. In comparison, the saturation magnetizations of PFS‐R1+@IONPs, PFS‐R2+@IONPs, and Sty‐R+@IONPs are significantly lower, measured at 17.5, 19.7, and 17.7 emu g^−1^, respectively. This reduction reflects the presence of non‐magnetic constituents in the composite materials, which dilute the magnetic signal relative to that of the bare IONPs.^[^ ^49^ ^]^

### 2.2 Equilibrium Removal of Multiple PFAS from Landfill Leachate

**Figure 2:**
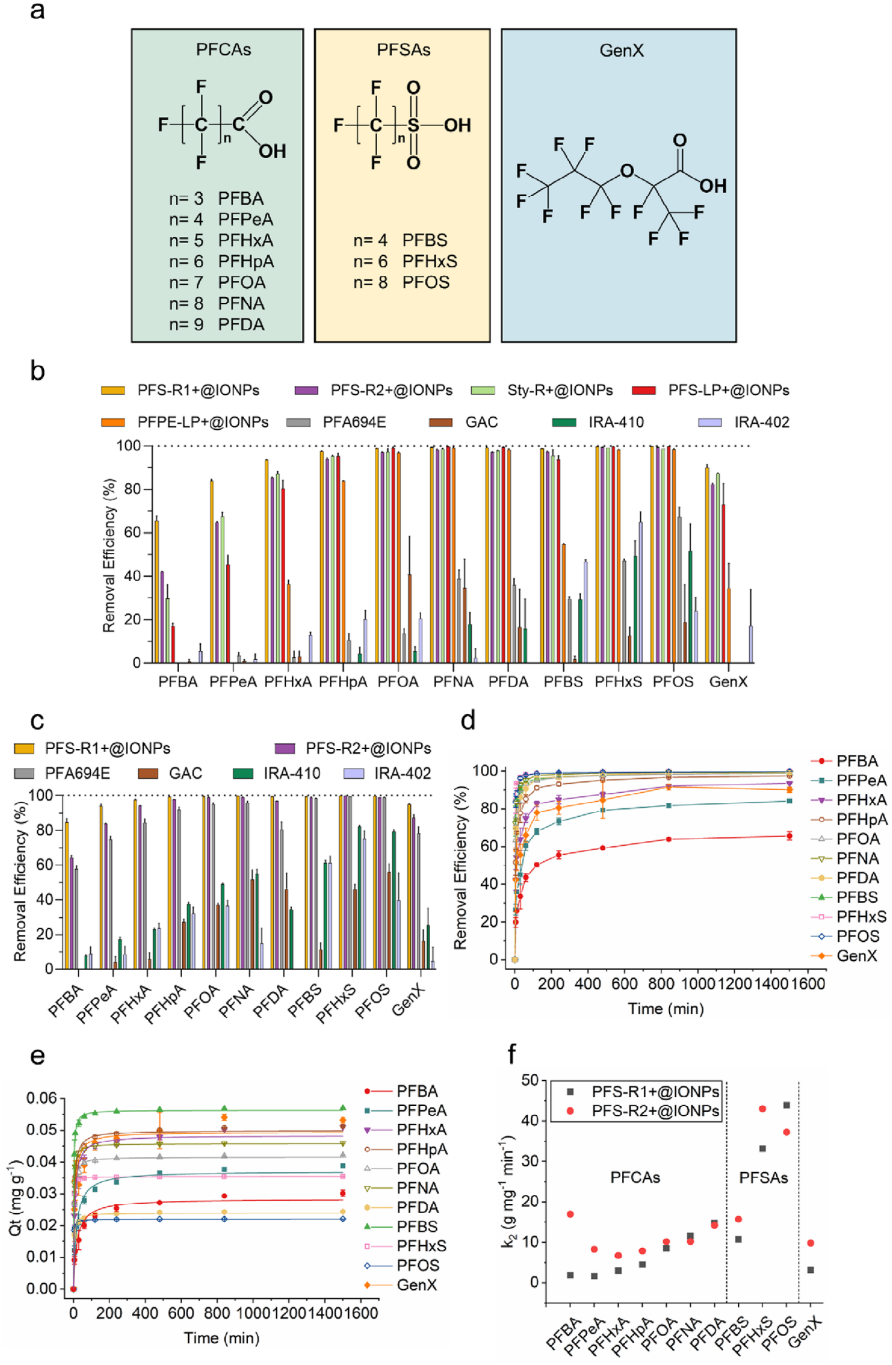
Removal of 11 different PFAS from landfill leachate by magnetic polymer sorbents. a) Chemical structures of the 11 PFAS tested in this work. b) Equilibrium sorption of the 11 PFAS by different types of sorbents at a relatively low initial sorbent concentration. c) Equilibrium sorption of the 11 PFAS by different types of sorbents at a relatively high initial sorbent concentration. d) Removal efficiency of the 11 PFAS by PFS‐R1+@IONPs as a function of sorption time. e) Experimental data from d) fitted by Pseudo‐second‐order. f) Rate constant *k*_2_ for the 11 PFAS by PFS‐R1+@IONPs and PFS‐R2+@IONPs. Sorbent: 2 mg mL^−1^ (excluding IONPs) each for b), d), e), and f), and 4 mg mL^−1^ (excluding IONPs) each for c). Each PFAS initial concentration: 44–118 ppb. Treatment duration: 25 h. pH 8.24. The results for b), c), d), and e) are the average of three replicates, and one standard deviation is shown.

The above magnetic polymer sorbents were employed to treat landfill leachate spiked with 11 different types of PFAS, including short‐chain perfluoroalkyl carboxylic acids (PFCAs, C*_n_* F_2_ *_n_*_+1_COOH, *n*<7), long‐chain PFCAs (*n*≥7), short‐chain perfluorosulfonic acids (PFSAs, C*_n_* F_2_ *_n_*_+1_SO_3_H, *n*<6), long‐chain PFSAs (*n*≥6), and an emerging PFAS ammonium salt of hexafluoropropylene oxide dimer acid (GenX) (**Figure** **2**a).^[^ ^34^, ^50^ ^]^ Before PFAS spiking, initial PFAS concentrations in landfill leachate were measured and are shown in Table S2 (Supporting Information). After spiking of the 11 PFAS, the initial concentration of each PFAS ranged from 44–118 parts per billion (ppb), and the sorption duration was set to 25 h. Upon complete sorption, a magnet was applied for rapid and efficient recovery of sorbents within 2 min.

Using liquid chromatography with tandem mass spectrometry (LC‐MS/MS), results in Figure S11 (Supporting Information) demonstrate that magnetic sorbents before quaternization show little‐to‐no removal of short‐chain PFCAs and PFSAs. Moderate removal of long‐chain PFCAs, including perfluorooctanoic acid (PFOA, 38.7%), perfluorononanoic acid (PFNA, 37.9%), and perfluorodecanoic acid (PFDA, 16.0%), as well as a long‐chain PFSA, perfluorooctanesulfonic acid (PFOS, 13.1%), was observed for PFS‐R1@IONPs. This effectiveness is primarily attributed to fluorous interactions between the magnetic sorbent and long‐chain PFAS, enabling selective recognition and removal.^[^ ^20^, ^24^, ^25^, ^51^ ^]^

Upon quaternization, PFS‐R1+@IONPs exhibits the best performance among the four cationic sorbent candidates, with the difference being particularly pronounced for the removal of short‐chain PFCAs (*n*<5, Figure 2b). For example, PFS‐R1+@IONPs shows a removal efficiency of 65.7% for perfluorobutanoic acid (PFBA, *n *= 3), outperforming PFS‐R2+@IONPs, Sty‐R+@IONPs, and PFS‐LP+@IONPs, which remove 42.0%, 29.6%, and 16.9%, respectively. For perfluoropentanoic acid (PFPeA), the removal reaches 84.1% for PFS‐R1+@IONPs, significantly higher than the values observed for PFS‐R2+@IONPs (64.8%), Sty‐R+@IONPs (67.6%), and PFS‐LP+@IONPs (45.3%).

Note that PFS‐R2+@IONPs is a variant with lower PFS content but higher cationic group content than PFS‐R1+@IONPs. Its lower PFAS removal could be attributed to the much stronger competitive binding with co‐contaminants in landfill leachate to the active sorption sites on PFS‐R2+@IONPs (Table S3, Supporting Information). Additionally, Sty‐R+@IONPs is a non‐fluorinated counterpart to PFS‐R1+@IONPs, highlighting the importance of fluorous interaction for efficient PFAS removal, especially in the complex matrix of landfill leachate. The reduced performance of PFS‐LP+@IONPs with linear polymer attachment on short‐chain PFCAs is primarily due to the significant loss of the sorbent fraction with a high cationic content, resulting from poor magnetic recovery during the purification process.

As the chain length of PFCAs increases from *n* = 3 to *n* = 8, PFS‐R1+@IONPs demonstrates progressively higher removal efficiencies, ranging from 65.7% for PFBA (*n* = 3) to 99.4% for PFNA (*n* = 8, Figure 2b). PFSAs show a similar trend, with removal efficiencies increasing in the order of perfluorobutanesulfonic acid (PFBS, *n* = 4), perfluorohexanesulfonic acid (PFHxS, *n* = 6), and PFOS (*n* = 8), achieving 98.8%, 99.7%, and 99.8%, respectively (Figure 2b). The increased PFAS removal with longer chain lengths aligns with previous studies,^[^ ^23^, ^24^, ^26^ ^]^ mainly due to the higher mobility of shorter‐chain PFAS, making them more difficult for the magnetic polymer sorbent to capture in landfill leachate. For PFCAs and PFSAs with the same number of carbons in their fluorinated backbone, the removal efficiencies of PFCAs are consistently lower than those of PFSAs, including PFPeA and PFBS (n = 4, 84.1% vs. 98.8%), perfluoroheptanoic acid (PFHpA) and PFHxS (n = 6, 97.4% vs. 99.7%), and PFNA and PFOS (*n* = 8, 99.4% vs. 99.8%) (Figure 2b). This difference can be attributed to two main factors: 1) the more hydrophobic nature of PFSAs compared to PFCAs, which leads to stronger hydrophobic and fluorous interactions between the PFAS and the PFS segments,^[^ ^52^ ^]^ and 2) the stronger negative inductive effect of the sulfonate group compared to the carboxylate group, due to resonance stabilization.^[^ ^53^ ^]^

Batch‐to‐batch reproducibility was assessed by synthesizing each of the four magnetic sorbents two additional times and evaluating their PFAS removal performance across all three batches (Figure S12, Supporting Information). As shown in Figure S13 (Supporting Information), the three crosslinked sorbents show slightly higher RSDs (5%–7%) for PFBA, while values for other PFAS are generally <4%. In contrast, PFS‐LP+@IONPs exhibits higher variability (RSDs of 7%–13%) for short‐chain PFCAs, including PFBA, PFPeA, and perfluorohexanoic acid (PFHxA), likely due to their low removal efficiencies and possibly variable magnetic recovery across batches. RSDs <5% were achieved for the rest eight PFAS. These results confirm reproducible performance across all sorbents,^[^ ^54^, ^55^ ^]^ with crosslinked systems offering greater batch‐to‐batch consistency.

Enhanced PFAS removal performance and increased chemical stability were observed after refining the chemical structure of the magnetic polymeric sorbent. PFPE‐LP+@IONPs, a cationic PFPE‐containing linear polymer‐coated magnetic IONPs from our previous work,^[^ ^24^ ^]^ shows no removal of the short‐chain PFCAs PFBA and PFPeA (Figure 2b). This indicates limited efficacy for the sorbent in removing short‐chain PFAS from landfill leachate. For the remaining nine PFAS, PFPE‐LP+@IONPs achieves removal rates between 34.6% and 99.1%. In contrast, all four quaternized magnetic sorbents developed herein demonstrate significantly higher removal efficiencies for all 11 PFAS, particularly PFS‐LP+@IONPs, which provides a direct comparison to PFPE‐LP+@IONPs. The poorer performance of PFPE‐LP+@IONPs is attributed to the instability of its chemical structure. To investigate this, an experiment was conducted to amplify the adverse effects by individually dispersing PFPE‐LP+@IONPs and PFS‐R1+@IONPs in aqueous solutions containing sodium phosphate, a common constituent in landfill leachate.^[^ ^56^, ^57^ ^]^ After 25 h of incubation, peaks a‐f from the grafted polymer were observed in the ^1^H NMR spectrum (Figure S14, Supporting Information), indicating polymer detachment and leaching into the solution. In addition, hydrolysis of the cationic functional groups, indicated by the appearance of a single peak h, suggests low stability of PFPE‐LP+@IONPs under basic conditions.^[^ ^38^ ^]^ In contrast, the solution treated with PFS‐R1+@IONPs shows no obvious new peaks, demonstrating greater chemical stability and improved adaptability of the current magnetic sorbent under varying environmental conditions.

All four magnetic polymer sorbents prepared in this work demonstrate superior performance compared to four commercially available sorbents, including ion‐exchange resins Purolite Purofine PFA694E, DuPont AmberLite IRA410 Cl, and DuPont AmberLite IRA402 Cl, as well as activated charcoal‐DARCO (20‐40 mesh particle size, granular), abbreviated as PFA694E, IRA‐410, IRA‐402, and GAC, respectively, under the same testing condition (Figure 2b). Among these, PFA694E is specifically designed for water treatment applications,^[^ ^58^ ^]^ however, it shows a low PFAS removal efficiency in landfill leachate (<30% and <70% for short‐chain and long‐chain PFAS, respectively). IRA‐402, another ion‐exchange resin containing quaternary ammonium functional groups, removes <66% of any of the 11 PFAS tested. IRA‐410 and GAC achieve even lower PFAS removal, with each individual PFAS showing <52% removal efficiency.

Importantly, increasing the dosage of the magnetic polymer sorbents from 2 to 4 and subsequently to 8 mg L^−1^ (excluding IONPs) increases the number of available sorption sites, thereby enhancing PFAS removal efficiency (Figure 2c; Figure S15, Supporting Information). For example, at 8 mg/L, PFS‐R1+@IONPs achieves removal efficiencies of 92.2% for PFBA and 97.5% for PFPeA, with >99% removal for the remaining nine PFAS. However, the performance difference between the synthesized magnetic sorbents and commercial sorbents becomes less pronounced at higher sorbent concentrations. No significant variation in PFAS removal was observed across the tested pH range of 6.3–10.0 (Figure S16, Supporting Information), indicating that the sorbent performance is pH‐independent within this interval. This finding aligns with previous studies demonstrating that quaternary ammonium groups retain a permanent positive charge across a wide pH range (pH 1–14),^[^ ^59^ ^]^ in contrast to primary, secondary, or tertiary amines that are pH‐dependent and only become protonated under acidic conditions.^[^ ^60^ ^]^ As PFAS are predominantly anionic under environmentally relevant conditions due to their low p*K*_a_ values (≈0),^[^ ^61^, ^62^ ^]^ the persistent cationic nature of quaternary ammonium functionalities ensures stable electrostatic interactions and effective PFAS binding across the tested pH range. pH values <6 were excluded from testing due to visible organic matter flocculation under acidic conditions (Figure S17, Supporting Information), which can largely alter the initial concentration of co‐contaminants and confound direct comparisons of PFAS removal performance.^[^ ^63^ ^]^

### 2.3 Multiple PFAS Sorption Kinetics of Magnetic Polymer Sorbents in Landfill Leachate

Sorption kinetics experiments for 11 PFAS were performed in landfill leachate, using both PFS‐R1+@IONPs and PFS‐R2+@IONPs as sorbents. The results shown in Figure 2d and Figure S18 (Supporting Information) demonstrate that all 11 PFAS reach sorption equilibrium after 25 h of treatment. The experimental data were fitted to two sorption kinetics models, the Pseudo‐first‐order and Pseudo‐second‐order models (Figure 2e; Figures S19 and S20, Supporting Information)^[^ ^49^ ^]^ as shown in Equations (1) and (2) below:

(1) $$Q_{t}=Q_{e}\left(1-e^{-k_{1}t}\right)$$

(2) $$Q_{t}=\frac{k_{2}Q_{e}^{2}t}{1+k_{2}Q_{e}t}$$

where *t* (min) represents the time elapsed during the sorption process, *Q*_t_ (mg g^−1^) is the amount of PFAS at time *t*, *Q*_e_ (mg g^−1^) is the equilibrium amount of PFAS when the system has reached equilibrium, *k*_1_ (min^−1^) is the rate constant for the process described by the Pseudo‐first‐order model, and *k*_2_ (g mg^−1^ min^−1^) is the rate constant for the process described by the Pseudo‐second‐order model.

Fitting parameters for sorption kinetics are summarized in Tables S4 and S5 (Supporting Information). The data fit the Pseudo‐second‐order model better than the Pseudo‐first‐order one for both magnetic polymer sorbents, as indicated by the higher coefficients of determination (R^2^) values for all 11 tested PFAS. This suggests that the sorption processes were likely driven by chemisorption rather than simple physisorption.^[^ ^64^, ^65^ ^]^

The second‐order rate constant, *k*_2_, which represents the sorption rate, was plotted for each PFAS based on the Pseudo‐second‐order model for both magnetic sorbents (Figure 2f). At a comparable initial concentration for each PFAS, PFS‐R1+@IONPs generally exhibits increasing *k*_2_ values with increasing PFAS chain length, indicating faster sorption kinetics for both PFCAs and PFSAs. Compared to PFS R1+@IONPs, PFS R2+@IONPs, which contains a higher content of quaternary ammonium groups but a lower content of PFS segments, shows larger *k*_2_s for short‐chain PFAS, the long‐chain PFSA PFHxS, and GenX, while comparable or smaller *k*_2_s were observed for the other long‐chain PFAS. These results indicate that the sorption of short‐chain PFAS is enhanced when the sorbent contains more cationic functional groups, facilitating stronger electrostatic attraction. However, an increased content of cationic groups does not necessarily improve PFAS removal performance. To be more specific, although PFS‐R2+@IONPs exhibits faster sorption kinetics for most tested PFAS compared to PFS‐R1+@IONPs, it shows lower PFAS removal efficiencies at equilibrium (after 25 h) (Figure 2b,d; Figure S18, Supporting Information). These findings highlight the importance of balancing the content of fluorinated segments and cationic functional groups to achieve both rapid and efficient removal of multiple PFAS from landfill leachate. Finally, for both sorbents, PFSAs exhibit larger *k*_2_ values than PFCAs with the same number of fluorinated carbon atoms, primarily attributed to a higher affinity between PFSAs and sorbents than PFCAs.

### 2.4 Sorption Isotherms of Magnetic Polymer Sorbents in Landfill Leachate

Sorption isotherms were performed using PFS‐R1+@IONPs, PFS‐R2+@IONPs, and PFA694E, to further reveal binding mechanisms between sorbents and PFAS in landfill leachate. As PFBS has the highest initial concentration at 21.2 ppb among all PFAS in the leachate (Table S2, Supporting Information), it was chosen for the following isotherm studies.

**Figure 3:**
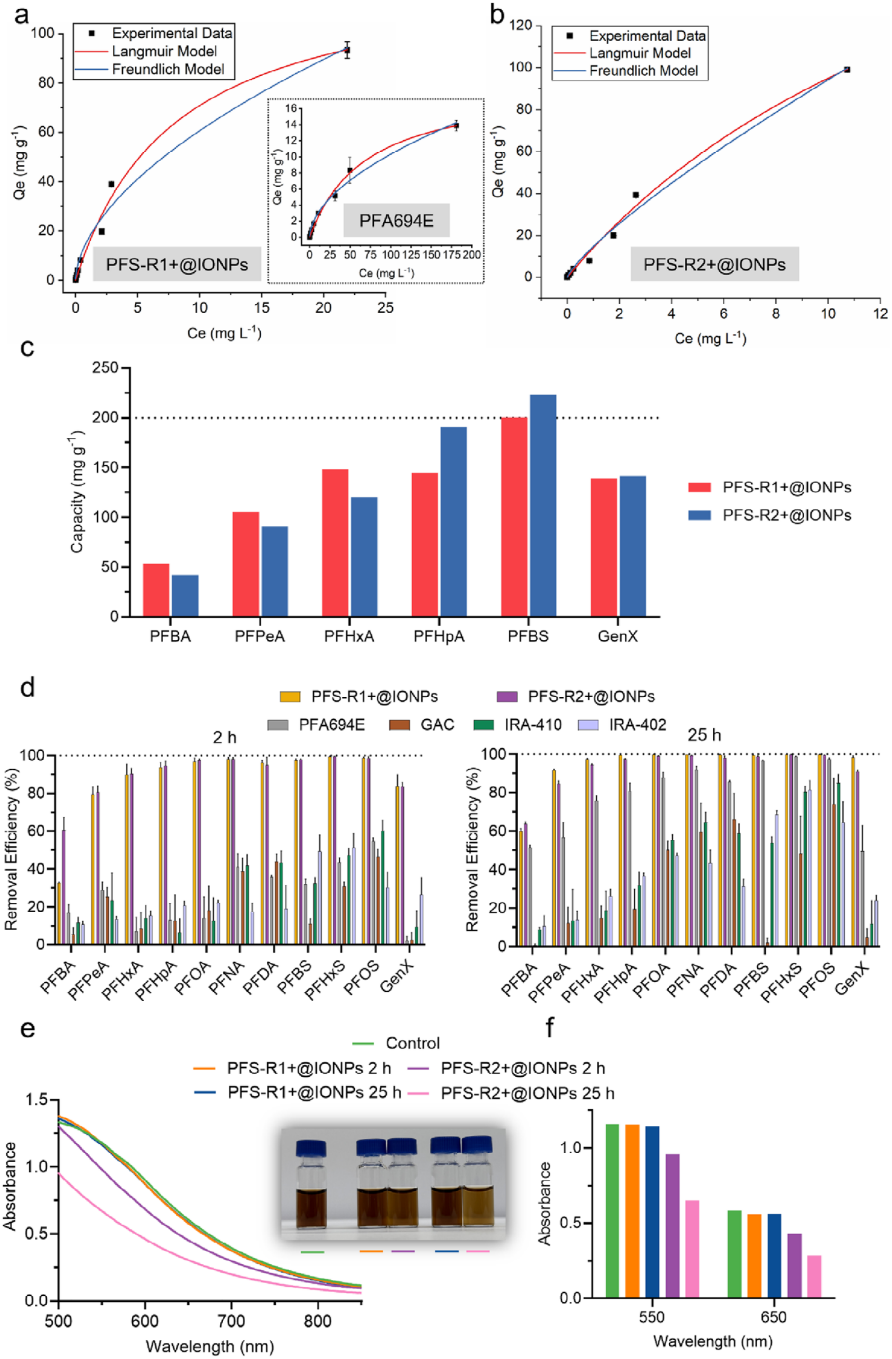
Sorption characteristics of PFAS in landfill leachate using newly developed magnetic polymer sorbents. a) Sorption isotherms of PFBS by PFS‐R1+@IONPs and PFA694E (inset). b) Sorption isotherms of PFBS by PFS‐R2+@IONPs. c) Sorption capacities of five short‐chain PFAS and GenX. d) Removal of 11 PFAS at environmentally relevant concentrations (pH 8.51) by PFS‐R1+@IONPs and PFS‐R2+@IONPs after treatment for either 2 h (left) or 25 h (right). e) Visible light absorbance of landfill leachate before and after treatment by either PFS‐R1+@IONPs or PFS‐R2+@IONPs. f) Absorbance intensity of leachate solution at 550 and 650 nm. Sorbent: 2 mg mL^−1^ (excluding IONPs) for a) and b); 1 mg mL^−1^ (excluding IONPs) for c); 4 mg mL^−1^ for d), e) and f). PFAS initial concentration: 0.1–250 ppm for a) and b); 0.25 mg mL^−1^ each for c); 22 ppb for PFBS and 1–5 ppb for the rest of PFAS for d), e), and f). Treatment duration: 27 h for a), b), and c); 2 or 25 h for d), e), and f). The results for a), b), and d) are the average of three replicates, and one standard deviation is shown.

After treating the leachate solution containing PFBS with initial concentrations ranging from 0.1 to 250 parts per million (ppm), experimental data for the three sorbents were fitted using two sorption isotherm models, i.e., Langmuir and Freundlich models (**Figure** **3**a,b)^[^ ^66^ ^]^ using their non‐linear curve fittings shown as Equations (3) and (4) below:

(3) $$Q_{e}=\frac{Q_{m}bC_{e}}{\left(1+bC_{e}\right)}$$

(4) $$Q_{e}=K_{F}C_{e}^{1/n}$$

where *C*_e_ is the residual concentration of PFBS at equilibrium (mg L^−1^), *Q*_m_ (mg g^−1^) is the estimated maximum sorption capacity, *Q*_e_ (mg g^−1^) is the amount of PFBS bound to the sorbent at equilibrium, *b* (L mg^−1^) is the Langmuir equilibrium constant, representing the binding affinity, *K*_F_ ((mg g^−1^)(L mg^−1^)^1/^ *^n^*) is the Freundlich constant, and *n* is the intensity of sorption.

Upon complete sorption, all three sorbents exhibit a better fit with the Langmuir model than the Freundlich model (Table S6, Supporting Information), as indicated by higher R^2^ values. This suggests that the interactions between sorbents and PFBS are more likely to involve monolayer sorption.^[^ ^67^ ^]^ Based on the Langmuir model, the estimated maximum sorption capacities for PFS‐R1+@IONPs, PFS‐R2+@IONPs, and PFA694E were calculated to be 128, 263, and 19.2 mg g^−1^, respectively. The higher sorption capacity of PFS‐R2+@IONPs (∼doubled compared to PFS‐R1+@IONPs) can be attributed to its higher content of quaternary ammonium groups, resulting in more active sorption sites for PFBS. However, PFS‐R2+@IONPs exhibits a lower binding affinity than PFS‐R1+@IONPs (0.056 vs. 0.124 L mg^−1^). This difference likely explains its lower equilibrium PFAS removal efficiencies compared with PFS‐R1+@IONPs, further highlighting the importance of balancing fluorous and electrostatic interactions. Compared to PFA694E, PFS‐R1+@IONPs and PFS‐R2+@IONPs exhibit ≈7 and ≈14 times higher capacity, as well as ≈9 and ≈4 times higher binding affinity, respectively. These results demonstrate the clear advantages of the developed magnetic polymer sorbents over commercially available IEX resins for PFAS removal from landfill leachate.

The individual sorption capacity of five short‐chain PFAS and GenX was further examined using ^19^F NMR (Figure 3c; Figures S21 and S22, Supporting Information).^[^ ^42^ ^]^ 2,2,2‐Trifluoroethanol was used as the internal standard to calculate the changes in PFAS concentrations before and after treatment with the sorbent. After equilibrium sorption, PFS‐R1+@IONPs shows higher capacities for PFBA, PFPeA, and PFHxA compared to PFS‐R2+@IONPs. However, for PFHpA with a longer‐chain length and the only short‐chain PFSA, PFBS, PFS‐R2+@IONPs exhibits higher capacities. Comparable sorption capacities for GenX were observed for both magnetic polymer sorbents. The lower sorption capacities for PFS‐R2+@IONPs on shorter‐chain PFCAs than PFS‐R1+@IONPs indicate the adverse effect from complex components on cationic groups of the sorbent in the landfill leachate, again, underscoring the importance of balancing fluorous and electrostatic interactions for efficient PFAS removal.

### 2.5 Removal of Multiple PFAS at Environmentally Relevant Concentrations from Landfill Leachate

In this section, the removal of 11 PFAS compounds was performed at environmentally relevant concentrations with initial concentrations of 22 ppb for PFBS and 1–5 ppb for the other ten PFAS. The solution was treated using either PFS‐R1+@IONPs or PFS‐R2+@IONPs for 2 and 25 h. The results in Figure 3d demonstrate that, after 2 h, PFS‐R2+@IONPs exhibits higher removal efficiencies than PFS‐R1+@IONPs for the majority of PFAS tested. When the treatment duration was extended to 25 h, substantial increases in removal efficiencies were observed, especially for PFCAs using PFS‐R1+@IONPs. In contrast, PFS‐R2+@IONPs shows no significant improvement upon prolonged treatment, indicating that the sorption reaches the equilibrium within 2 h. At equilibrium, PFS‐R1+@IONPs demonstrates superior performance in removing all 11 PFAS compared to PFS‐R2+@IONPs, which is consistent with the results shown in Figure 2d and Figure S18 (Supporting Information). Both magnetic polymer sorbents demonstrate consistently superior performance compared to commercial alternatives after both 2 and 25 h of treatment (Figure 3d).

We hypothesize that the presence of complex components i.e., co‐contaminants from the landfill leachate, has stronger interactions with PFS‐R2+@IONPs than PFS‐R1+@IONPs through electrostatic interactions. Such interactions likely contribute to the reduced PFAS removal efficiency for PFS‐R2+@IONPs. In addition, components such as humic substances and metals are known to lead to a brown to dark color in landfill leachate.^[^ ^68^, ^69^ ^]^ Therefore, we monitored changes in absorbance using an ultraviolet‐visible (UV–vis) spectrophotometer, focusing on the visible wavelength range of 500‐800 nm before and after treatment.^[^ ^70^, ^71^ ^]^

Figure 3e shows that within the tested wavelength range, no significant difference in absorbance was observed when comparing the untreated leachate with the ones treated by PFS‐R1+@IONPs at both 2 and 25 h, suggesting minimal to no sorption of co‐contaminants. In contrast, PFS‐R2+@IONPs exhibits significantly lower absorbance across the entire wavelength range after 2 h of treatment, with a further decrease in absorbance at 25 h. This reduction in absorbance indicates much stronger interactions and higher removal of co‐contaminants by PFS‐R2+@IONPs (Figure 3f). Therefore, although PFS‐R2+@IONPs has faster sorption kinetics on the majority of PFAS tested, its active sorption sites are largely occupied by co‐contaminants, such as humic substances, reducing their PFAS removal efficiency (Figure 3d).

Compared to previous studies, the magnetic polymer sorbents developed in this work exhibit significantly enhanced performance in removing PFAS from landfill leachate at environmentally relevant concentrations.^[^ ^72^, ^73^, ^74^, ^75^, ^76^ ^]^ Among these, the most relevant is the coal‐based magnetic activated carbon (MAC) developed by Zhang et al.,^[^ ^72^ ^]^ which showed 72.8%–89.6% removal of five different PFCAs, including PFHxA, PFHpA, PFOA, PFNA, and perfluorododecanoic acid, from landfill leachate at ppb levels. The authors used ≈53.3 mg mL^−1^ of MAC for treatment of PFAS, and did not find a further increase in PFAS removal performance with a higher sorbent dosage. In contrast, the magnetic sorbents like PFS‐R1+@IONPs prepared in this work, show 97.4%–99.9% removal for the same PFAS at a much lower dosage of 4 mg mL^−1^. In addition, the saturation magnetization of MAC was measured to be ≈4.9 emu g^−1^, much lower than the saturation magnetization i.e., 17.5–19.7 emu g^−1^ of the magnetic polymer sorbents developed in this work, indicating improved magnetic separation efficiency. Other reported sorptive methods using nonmagnetic sorbents also showed limited removal efficiency or capacity (e.g., <50 mg g^−1^ for PFBS) in landfill leachate.^[^ ^73^, ^74^, ^75^, ^76^ ^]^

The performance of the magnetic polymer sorbents against 11 PFAS (≈1 ppb) was further assessed in different water matrices, including drinking water and compost leachate (Tables S7 and S8, Figure S23, Supporting Information). After 2 h, PFS‐R1+@IONPs demonstrates removal efficiencies of 99.1% for PFBA and >99.7% for the other PFAS in drinking water, while PFS‐R2+@IONPs exhibits >99.7% removal for all PFAS. In compost leachate, both sorbents show removal efficiencies of 99.1%–99.2% for PFBA and >99.5% for the other PFAS. These results demonstrate the strong adaptability of the magnetic sorbents across water types, and highlight the ongoing challenge of PFAS removal from complex matrices such as landfill leachate.

### 2.6 Molecular Dynamics (MD) Simulations of Fluorous and Electrostatic Interactions Between Polymer Sorbents and PFAS in Complex Aqueous Media

**Figure 4:**
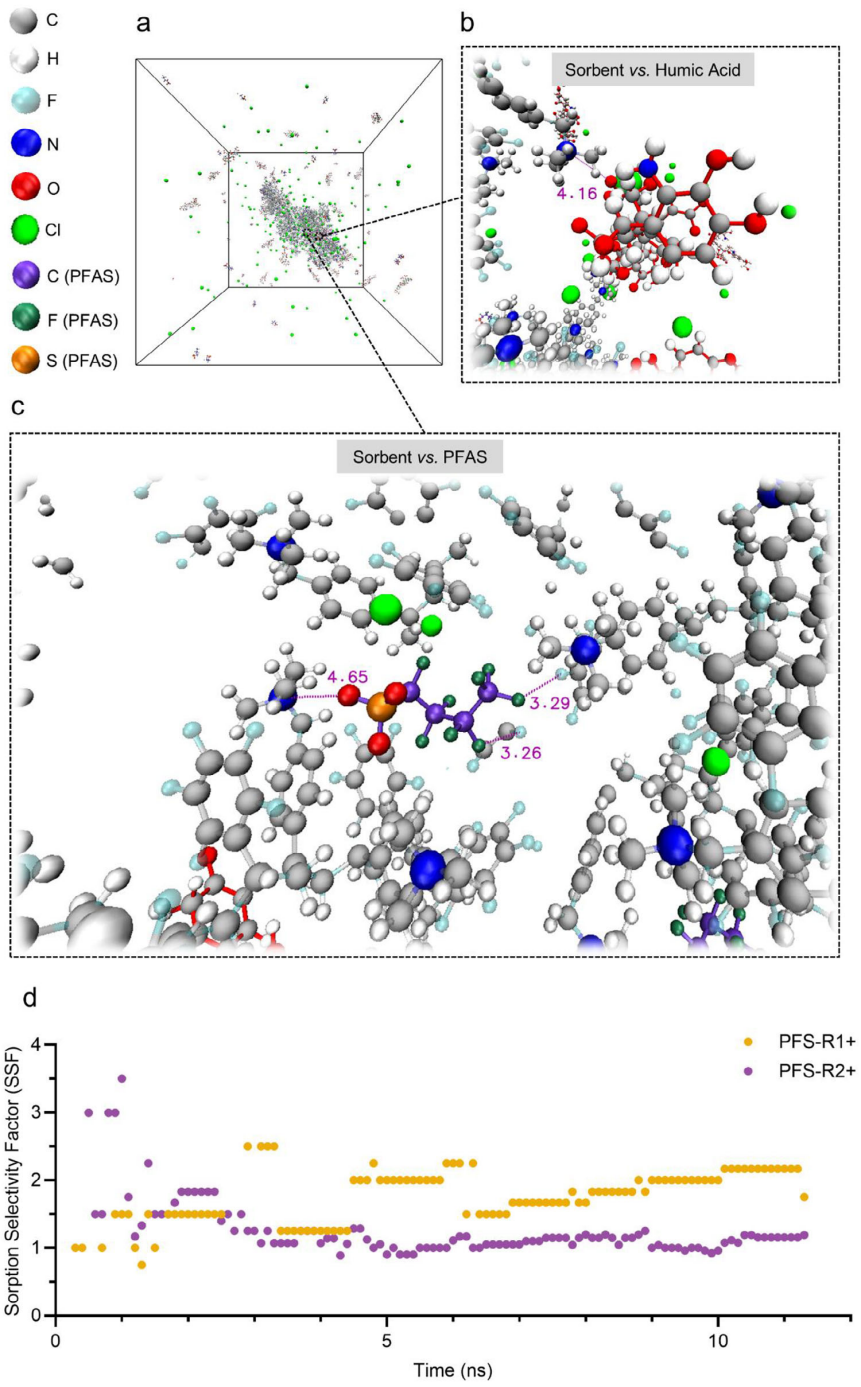
MD simulations of PFAS in water with simplified models of humic acid and either PFS R1+ or PFS R2+ sorbent. a) Snapshot of the simulation box for a mixture of PFBS, PFS‐R1+, and humic acid in water at 11 ns. b) Snapshot of humic acid interacting with PFS‐R1+ at 11 ns. c) Snapshot of PFBS interacting with PFS‐R1+ at 11 ns. d) SSFs of PFS‐R1+ and PFS‐R2+ for PFBS as a function of simulation time. Different atoms in the simulation box are shown in distinct colors. The missing data points in d) indicate infinite selectivity at the corresponding simulation time, due to the absence of humic acid sorption.

The balance between fluorous and electrostatic interactions governing PFAS removal from landfill leachate was examined at the molecular level by MD simulations (**Figure** **4**a; Tables S9–S12, Supporting Information). Humic acid was included to mimic the leachate matrix. Simplified models were used for humic acid (Figure S24, Supporting Information) and for both PFS‐R1+ and PFS‐R2+ (Figures S25 and S26, Supporting Information), to enable efficient MD simulations by capturing key interactions while reducing computational cost and structural complexity.^[^ ^77^ ^]^ PFBS was chosen as the representative PFAS due to its highest concentration among all PFAS identified in the landfill leachate. The simplified PFS‐R2+ model comprises 20 vinylbenzyltrimethylammonium chloride (VBTAC) and ten PFS units, while the PFS‐R1+ model contains ten VBTAC and 20 PFS units. Each simulation system includes 20 polymer chains forming a central spherical aggregate, surrounded by 50 PFAS molecules, 25 humic acid molecules, and 4 × 10^5^ water molecules. A molecule (either PFAS or humic acid) is defined as sorbed if any of its atoms approach within 4.5 Å of any atom on the polymer in at least one trajectory frame, reflecting a typical first solvation shell distance.^[^ ^78^, ^79^ ^]^

Figure 4b demonstrates that humic acid occupies active sites on PFS‐R1+ through electrostatic attraction. The N…O distance between the quaternary ammonium nitrogen and a carboxylate oxygen was measured to be 4.16 Å, confirming the inhibitory effect of humic acid. Figure 4c reveals that PFBS interacts with PFS‐R1+ through fluorous interactions, with F…F distances of 3.26 and 3.29 Å between PFBS and the perfluoroalkyl segments of the polymer. Additional electrostatic contacts were detected, with the N…O distance between the quaternary ammonium nitrogen and the PFBS sulfonate oxygen of 4.65 Å being observed, while a closer 2.59 Å contact occurs between an ammonium methyl group and the same oxygen (Figure S27, Supporting Information). These findings show that humic acid competes with PFAS, and that both fluorous and electrostatic forces drive PFBS sorption.

Time‐resolved sorption ratios for PFBS and humic acid (FigureS28, Supporting Information) indicate faster uptake by PFS‐R2+, reaching 62% PFBS and 52% humic acid removal within the simulation window, compared with 28% and 16%, respectively, for PFS‐R1+. These trends are consistent with the experimental observations in Figures 2, 3, and Figure S18 (Supporting Information).

In this work, PFAS selectivity was quantified by the sorption‐selectivity factor (SSF) using Equation (5) below:^[^ ^80^ ^]^

(5) $$SSF=\frac{SR_{\mathrm{PFAS}}}{SR_{\mathrm{HA}}}$$

where *SR*_PFAS_ and *SR*_HA_ are the sorption ratios of PFBS and humic acid, respectively.

Figure 4d shows that the SSF for PFS‐R2+ decreases from ≈3 to ≈1 within the first 3 ns and then stabilizes, indicating a loss of PFBS selectivity as humic acid sorption progresses. In contrast, PFS‐R1+ generally maintains SSF>1 throughout the 11 ns simulation, increasing from 1–1.5 before 2.5 ns to 1.25–2.5 between 2.9 and 11 ns. Although its initial selectivity is lower, PFS‐R1+ exhibits more sustained and consistent PFAS preference. The MD simulation results align with the findings from Figure 3d–f, reinforcing the importance of optimizing the balance between fluorinated segments and cationic sites for selective PFAS removal in complex matrices.

### 2.7 A Compact and Portable Device for Multiple PFAS Removal at Environmentally Relevant Concentrations from Landfill Leachate

**Figure 5:**
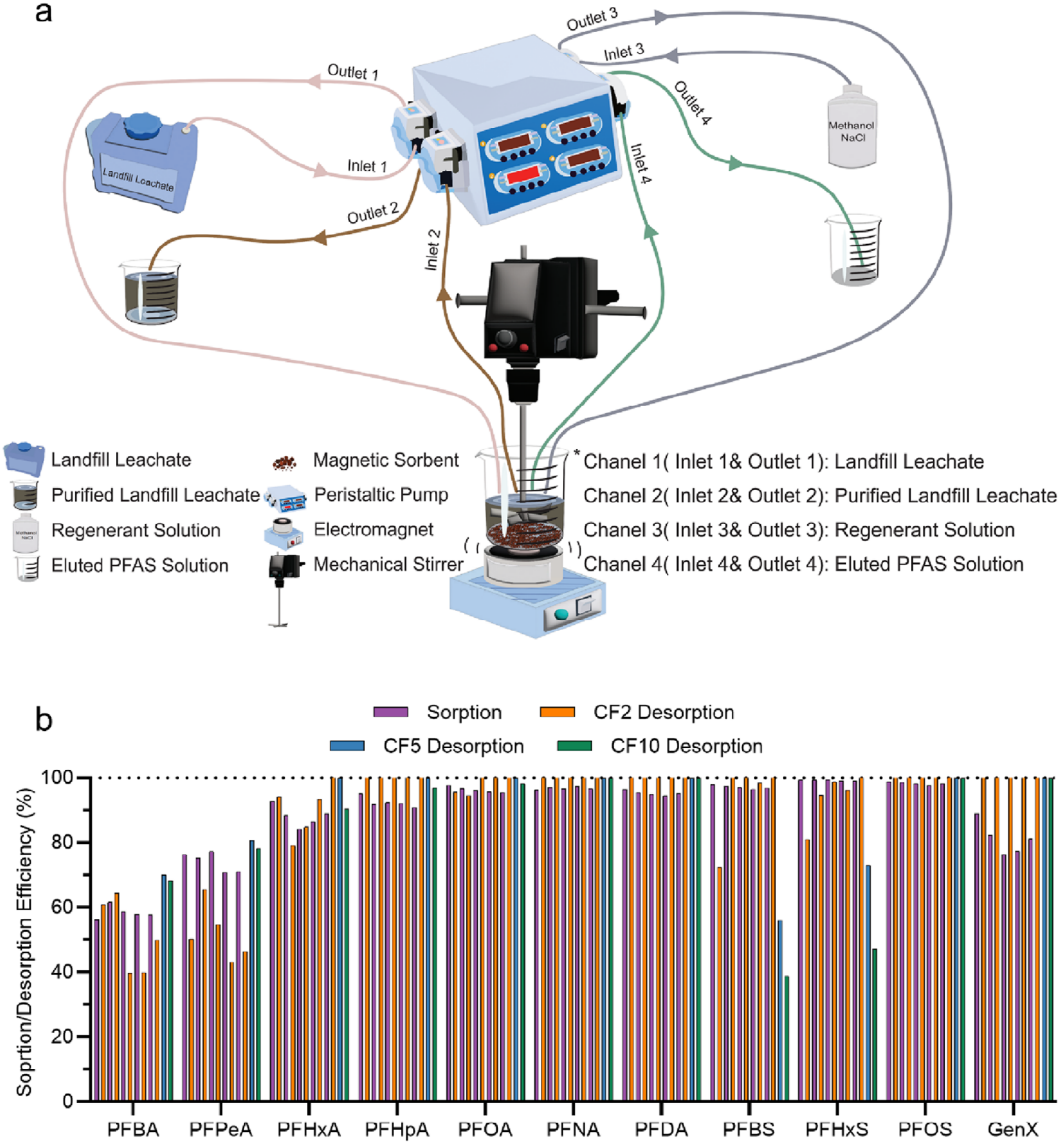
Continuous removal of 11 PFAS at environmentally relevant concentrations and sorbent regeneration using a compact and portable device. a) Schematic illustration of the device for both sorption and desorption experiments. b) Sorption and desorption efficiencies of 11 PFAS over five regeneration cycles at CF = 2, and desorption efficiencies at CF = 5 and 10. Sorbent: 4 mg mL^−1^ (excluding IONPs). PFAS initial concentration: 22 ppb for PFBS and 1–5 ppb for the rest of PFAS. Treatment duration: 2 h for sorption, and 30 min for desorption. pH 8.51.

Batch experiments provide critical insights into the sorption characteristics of the developed magnetic polymer sorbents. Building on these findings, a compact and portable device was designed for continuous PFAS removal from landfill leachate and for sorbent regeneration. The setup incorporates a four‐channel peristaltic pump and an electromagnet (**Figure** **5**a). Each channel of the pump operates independently and is equipped with both inlet and outlet tubing. A 100 mL aliquot of landfill leachate, consistent with that used in Section 2.5, was introduced into a beaker containing PFS‐R2+@IONPs via the peristaltic pump. PFS‐R2+@IONPs was selected due to its markedly faster sorption kinetics for most PFAS tested compared to PFS‐R1+@IONPs, facilitating efficient PFAS capture within short contact times. After stopping the pump, mechanical stirring was applied for 2 h to ensure thorough mixing and interaction between the sorbent and PFAS. Upon completion, the treated leachate was extracted through the second channel for LC‐MS/MS analysis, and the sorbent was recovered using an electromagnet. As shown in Figure 5b (purple columns), all 11 PFAS compounds were efficiently removed, with >90% removal observed for the majority.

After complete removal of the treated leachate solution, 50 mL of sodium chloride methanol solution was introduced via the third channel of the peristaltic pump^[^ ^42^ ^]^ with the electromagnet deactivated. The mixture was stirred for 30 min to facilitate desorption, after which the electromagnet was reactivated to separate the sorbent, and the supernatant was collected through the fourth channel for LC‐MS/MS analysis. Results shown in Figure 5b (orange columns) demonstrate a general increase in desorption efficiency of PFS‐R2+@IONPs with increasing PFAS chain length. For PFCAs, PFBA (*n *= 3) and PFPeA (*n *= 4) exhibit ≈50%–60% release from the magnetic polymer sorbent, while PFHxA (*n *= 5) demonstrates 94.1% desorption. As the chain length of PFCAs increases further, ≈100% desorption efficiency was observed. Similarly, for PFSAs, 72.4% of PFBS (*n *= 4) was released, followed by PFHxS (*n *= 6) and PFOS (*n *= 8), with desorption efficiencies of 81.0% and ≈100%, respectively. GenX also exhibits ≈100% release.

The cycle was extended, with each cycle consisting of both sorption and desorption processes (Figure 5b). No significant loss in sorption and desorption efficiency was observed for all 11 PFAS over five cycles, indicating that 1) our magnetic sorbent can effectively remove various types of PFAS in practical scenarios continuously, and 2) the sorbent can be regenerated and reused over multiple cycles, potentially enhancing their economic feasibility. FT‐IR analysis after five cycles reveals no notable reduction in peaks associated with aromatic C═C stretching vibrations (1460–1540 cm^−1^) or bending vibrations of the trimethyl groups in quaternary ammonium cations (820–900 cm^−1^) (Figure S29, Supporting Information), confirming the structural stability of the sorbent after repeated use.

The above regeneration process was performed at a concentration factor (CF) of 2. Higher CFs of 5 and 10 were achieved by reducing the regenerant volume from 50 to 20 mL and 10 mL, respectively. As shown in Figure 5b, comparable regeneration efficiencies were observed relative to CF = 2, except for PFBS and PFHxS, which exhibit reduced efficiencies of 55.9% and 73.0% at CF = 5 (blue columns), and 38.7% and 47.2% at CF = 10 (green columns), respectively. The decline in regeneration efficiency with increasing CF is consistent with our previous study,^[^ ^42^ ^]^ in which a continuous‐flow system treating potable water revealed that PFSAs (e.g., PFBS and PFHxS) eluted significantly later than PFCAs and GenX under limited regenerant volumes. These findings provide insight into the influence of regenerant volume on desorption efficiency and guide the design of up‐scaled treatment systems using magnetic polymer sorbents, particularly for landfill leachate.

## 3 Conclusion

Magnetic non‐PFAS‐based polymer sorbents show great promise for removing various types of PFAS from landfill leachate through a combination of fluorous and electrostatic interactions. The design and preparation of magnetic PFAS sorbents highlight two key findings: 1) the use of a crosslinked polymer matrix facilitates efficient magnetic separation and sorbent recovery, and 2) balancing fluorinated and quaternary ammonium functional groups leads to optimized PFAS removal performance. PFS‐R2+@IONPs, a magnetic polymer sorbent containing a greater proportion of cationic functional groups and a lower proportion of fluorinated segments compared with PFS‐R1+@IONPs, exhibits faster sorption kinetics for most PFAS but lower equilibrium removal efficiencies under low initial PFAS concentrations. When evaluated for PFBS, the predominant PFAS species in the landfill leachate examined, PFS‐R2+@IONPs shows markedly greater sorption capacity than PFS‐R1+@IONPs but lower PFAS selectivity in the presence of competing co‐contaminants. The magnetic polymer sorbents developed in this study outperform four commercially available PFAS sorbents, i.e., PFA694E, GAC, IRA‐410, and IRA‐402. Efficient PFAS removal (>90% for the majority of the tested PFAS), along with excellent regeneration and reusability over five cycles, was successfully demonstrated through a continuous and portable device. Overall, this study highlights the developed magnetic sorbents as promising candidates to potentially bridge the gap between market demands and existing solutions for PFAS remediation in landfill leachate.

## Conflict of Interest

The authors declare no competing financial interest. Commercial sorbents used in this study were purchased through standard laboratory suppliers: Purolite Purofine PFA694E (Ecolab), DuPont AmberLite IRA410 Cl (DuPont), DuPont AmberLite IRA402 Cl (DuPont), and activated charcoal ‐ DARCO (Sigma‐Aldrich/Merck). All sorbents were tested independently, and the manufacturers were not involved in the study design, data collection, analysis, or interpretation.

## Supporting information



Supporting Information

## Data Availability

The data that support the findings of this study are available from the corresponding author upon reasonable request.
