# Supplementary material for: Non‐PFAS‐Based Magnetic Polymer Sorbents for Efficient Removal of Perfluorinated Compounds from Landfill Leachate
Source: Adv Mater. 2025 Sep 15;38(1):e02427. doi: 10.1002/adma.202502427 (PMC12759220; doi:10.1002/adma.202502427)
Supplement: Supplementary file 1 — Supporting Information [file ADMA-38-e02427-s001.docx]

**Supporting Information**

Non-PFAS-Based Magnetic Polymer Sorbents for Efficient Removal of Perfluorinated Compounds from Landfill Leachate

Xiao Tan,^†,‡^ Zhuojing Yang,^†,‡^ Zhou Chen,^†,‡^ Yutong Zhu,^†,‡^ Chunrong Yu,^†,‡^ Yiqing Wang,^†,‡^ Kehan Liu,^†,‡^ Pradeep Dewapriya,^§^ Xuemei Li,^†,‡^ Zicheng Su,^†,‡^ Marcus J. Giansiracusa,^⊥^ Colette Boskovic,^⊥^ Biao Wang,^†,‡^ Kevin V. Thomas,^§^ and Cheng Zhang*^†,‡^

*^†^Australian Institute for Bioengineering and Nanotechnology, The University of Queensland, Brisbane, QLD 4072, Australia*

*^‡^Centre for Advanced Imaging, The University of Queensland, Brisbane, QLD 4072, Australia*

*^§^Queensland Alliance for Environmental Health Sciences (QAEHS), The University of Queensland, Woolloongabba, QLD 4102, Australia*

*^⊥^School of Chemistry, University of Melbourne, Parkville, Victoria, 3010, Australia*

Corresponding author: c.zhang3@uq.edu.au

**Experimental Section**

*Materials:* The initiator 2,2′-azobis(2-methylpropionitrile) (AIBN) was recrystallized twice from methanol before use. Heptafluorobutyric acid (PFBA, 96 %, CAS: 375-22-4), perfluoropentanoic acid (PFPeA, 97 %, CAS: 2706-90-3) and perfluoroheptanoic acid (PFHpA, 96 %, CAS: 375-85-9) were purchased from ThermoFisher Scientific. Undecafluorohexanoic acid (PFHxA, 97 %, CAS: 307-24-4) was purchased from FUJIFILM Wako Pure Chemical Corporation. Ammonium perfluoro(2-methyl-3-oxahexanoate) (GenX, 97 %, CAS: 62037-80-3) was purchased from Apollo Scientific. Perfluorooctanesulfonic acid (PFOS, CAS: 1763-23-1) was purchased from Synquest Laboratories. Perfluorooctanoic acid (PFOA, 95 %, CAS: 335-67-1), perfluorononanoic acid (PFNA, 97 %, CAS: 375-95-1), perfluorodecanoic acid (PFDA, 98 %, CAS: 335-76-2), nonafluorobutane-1-sulfonic acid (PFBS, 97 %, CAS: 375-73-5) and tridecafluorohexane-1-sulfonic acid potassium salt (PFHxS salt, CAS: 3871-99-6) were purchased from Sigma Aldrich. Milli-Q water with a resistivity of 18.2 MΩ/cm was used throughout the experimental windows. All other chemicals were purchased from Sigma-Aldrich and used as received. Commercial sorbents used in this study were purchased through standard laboratory suppliers: Purolite^TM^ Purofine^TM^ PFA694E (Ecolab), DuPont^TM^ AmberLite^TM^ IRA410 Cl (DuPont), DuPont^TM^ AmberLite^TM^ IRA402 Cl (DuPont), and activated charcoal - DARCO^®^ (20-40 mesh particle size, granular, Sigma-Aldrich/Merck). Landfill leachate and compost leachate were kindly provided by Gold Coast City Council and NuGrow Pty Ltd, respectively. Drinking water was collected from municipal tap water in Brisbane.

*Synthesis of PFS-R1*: In a typical reaction, PFS (2 g, 10.30 mmol), VBC (90%, 874 mg, 5.15 mmol), triethoxyvinylsilane (TEVS, 97%, 4.04 g, 20.59 mmol), and divinylbenzene (DVB, 80%, 419.2 mg, 2.58 mmol), AIBN (147.4 mg, 0.90 mmol) were dissolved in 3 mL of α,α,α-trifluorotoluene (TFT) in the presence of a magnetic stirrer. The solution mixture was degassed for 25 minutes using nitrogen, followed by reacting at 70 °C for 48 hours in an oil bath. Upon complete reaction, the crude mixture was dried under high vacuum using an oil pump for 10 minutes at room temperature, and crude resin was then taken out from the reaction vial and ground into powder. The resin was washed with diethyl ether, followed by centrifugation. The purification cycle was repeated five times. The product, PFS-R1, was obtained by evaporating the excess diethyl ether under high vacuum at 70 °C using an oil pump for 12 hours.

*Synthesis of PFS-LP*: In a typical reaction, PFS (2 g, 10.30 mmol), VBC (90%, 874 mg, 5.15 mmol), TEVS (97%, 4.04 g, 20.59 mmol) and AIBN (138.3 mg, 0.84 mmol) were dissolved in 6 mL of TFT in the presence of a magnetic stirrer. The solution mixture was deoxygenated in an ice bath for 25 minutes using nitrogen, followed by reacting at 70 °C in an oil bath for 48 hours. The reaction was then quenched by putting the solution mixture on ice and exposing to air. The polymer was purified by precipitating the solution mixture into large excess amount of methanol (50 mL), followed by centrifugation. The precipitate was redissolved into small amount of diethyl ether and precipitated again into the methanol. The purification cycle was repeated four times. The product, PFS-LP, was obtained by evaporating excess methanol at 60 °C for 12 hours under high vacuum using an oil pump.

*Synthesis of PFS-R2*: In a typical reaction, PFS (874 mg, 4.50 mmol), VBC (90%, 2 g, 11.79 mmol), TEVS (97%, 4.04 g, 20.59 mmol), DVB (80%, 419.2 mg, 2.58 mmol), and AIBN (147 mg, 0.90 mmol) were dissolved in 3 mL of TFT in the presence of a magnetic stirrer. The solution mixture was deoxygenated in an ice bath for 25 minutes using nitrogen, followed by reacting at 70 °C in an oil bath for 48 hours. Upon complete reaction, the crude mixture was dried under high vacuum using an oil pump for 10 minutes at room temperature, and crude resin was then taken out from the reaction vial and ground into powder. The resin was washed with diethyl ether, followed by centrifugation. The purification cycle was repeated five times. The product, PFS-R2, was obtained by evaporating the excess diethyl ether under high vacuum at 70 °C using an oil pump for 12 hours.

*Synthesis of Sty-R*: In a typical reaction, styrene (2 g, 19.20 mmol), VBC (90%, 874 mg, 5.15 mmol), TEVS (97%, 4.04 g, 20.59 mmol), and DVB (80%, 419.2 mg, 2.58 mmol), AIBN (147.4 mg, 0.90 mmol) were dissolved in 3 mL of TFT in the presence of a magnetic stirrer. The solution mixture was degassed for 25 minutes using nitrogen, followed by reacting at 70 °C for 48 hours in an oil bath. Upon complete reaction, the crude mixture was dried under high vacuum using an oil pump for 10 minutes at room temperature, and crude resin was then taken out from the reaction vial and ground into powder. The resin was washed with diethyl ether, followed by centrifugation. The purification cycle was repeated five times. The product, Sty-R, was obtained by evaporating the excess diethyl ether under high vacuum at 70 °C using an oil pump for 12 hours.

*Synthesis of bare magnetic iron oxide nanoparticles (IONPs)*: The magnetic IONPs were prepared using the co-precipitation method. Typically, ferric chloride hexahydrate (FeCl_3_·6H_2_O, 2.7 g, 10 mmol) and ferrous chloride (FeCl_2_, 0.63 g, 5 mmol) were dissolved in deionized water (50 mL) in a round bottom flask with a magnetic stirrer. The solution mixture was deoxygenated for 40 minutes by nitrogen, followed by dropwise addition of excess 25% (*w/w*) of ammonia water (7 mL, 45 mmol) with stirring at a constant rate of 1000 revolutions per minute (rpm) at room temperature. The color of the solution changed to black rapidly, and the reaction mixture was stirred continuously for 40 minutes. The particles were then magnetically recovered using a large neodymium magnet (N50) and washed three times with Milli-Q water for purification. The magnetic IONPs were obtained through freeze-drying.

*Preparation of PFS-R1@IONPs*: Before producing PFS-R1@IONPs, both PFS-R1 and magnetic IONPs were further ground into powder. In a typical reaction, bare magnetic IONPs (250.9 mg) and PFS-R1 (1g) were dispersed in a mixed solution containing Milli-Q water (7 mL), ammonia water (3 mL, 25% *w/w*), and ethanol (40 mL) with a magnetic stirring. After reacting at room temperature for 25 hours, a large neodymium magnet (N50) was applied for 5 minutes to recover the PFS-R1@IONPs, and the solution was discarded. The magnetic PFS-R1@IONPs were washed five times with excess amout of ethanol, and recovered by the magnet in each cycle. The product, PFS-R1@IONPs, were obtained by evaporating excess ethanol under high vacuum at 70 °C using an oil pump for 12 hours. The grafting procedures of magnetic IONPs onto PFS-LP, PFS-R2, and Sty-R were the same as that for PFS-R1.

*Quaternization of PFS-R1@IONPs to Produce PFS-R1+@IONPs*: In a typical quaternization reaction, 10 mL of trimethylamine solution (31-35% *w/w*) was added into a solution with 1 g of PFS-R1@IONPs dispersed in 5 mL of ethanol. The solution mixture was transferred into an oil bath and reacted at 40 °C for 36 hours. The reactor was shaken every 12 hours to re-disperse the magnetic PFS-R1@IONPs during the reaction. Upon complete reaction, PFS-R1+@IONPs was recovered using a large neodymium magnet (N50) for 10 minutes and the solution was discarded. The magnetic PFS-R1+@IONPs were washed five times with excess Milli-Q water, and recovered by the magnet in each cycle. PFS-R1+@IONPs were obtained by freeze-drying. The same procedure was applied for PFS-LP@IONPs, PFS-R2@IONPs, and Sty-R@IONPs to produce PFS-LP+@IONPs, PFS-R2+@IONPs, and Sty-R+@IONPs.

*Equilibrium Sorption of 11 PFAS Using Different Types of Sorbents*: Eight magnetic polymeric sorbents, including PFS-LP+@IONPs, PFS-R1+@IONPs, PFS-R2+@IONPs, Sty-R+@IONPs, PFS-R1@IONPs, PFS-R2@IONPs, Sty-R@IONPs, and the one prepared in our previous work, namely PFPE-LP+@IONPs, along with four commercially available sorbents *i.e.* including a PFAS-specific ion-exchange (IEX) resin PFA694E, granular activated carbon (GAC), and two standard IEX resins IRA-410 and IRA-402, were used for the equilibrium sorption study. 11 PFAS, including PFBA, PFPeA, PFHxA, PFHpA, PFOA, PFNA, PFDA, PFBS, PFHxS PFOS, and GenX, were manully spiked into the original landfill leachate and created initial concentrations of 44-118 parts per billion (ppb). For each sorbent, 5 mL of the above prepared PFAS stock solution in landfill leachate was added into a glass vial with 10 mg (excluding weight of IONPs for the prepared polymeric sorbents@IONPs based on TGA results) of the linear polymer/crosslinked-resin/commercailly available sorbent, to create a final concentration of linear polymer/crosslinked-resin/commercailly available sorbent of 2 mg/mL. After sorption for 25 hours on a shaker at room temperature, a large neodymium magnet (N50) was used for recovering the magnetic sorbents for 2 minutes, followed by collection of 1.5 mL of the residue solution and centrifugation at 7000 rpm for 15 minutes to remove natural organic matters. The supernatant (800 μL) was carefully collected for subsequent LC-MS/MS analysis. For the four commercailly availbale sorbents, direct centrifugation at 7000 rpm for 15 minutes was performed. The above experiments were performed in triplicate. Control experiments without addition of any sorbents were also performed under identical conditions. Each of the four magnetic sorbents after quaternization was synthesized in two additional batches, and equilibrium sorption experiments were conducted once for each batch under the same conditions as described above to evaluate batch-to-batch reproducibility.

For the sorbent dosage study (at 4 and 8 mg/mL (excluding IONPs)), experiments were performed in triplicate using the same stock landfill leachate solution and following identical procedures to ensure consistency. For the pH-dependent study, the leachate (spiked with 11 PFAS) pH was adjusted using HCl or NaOH to the desired values, and each condition was tested in triplicate at a sorbent dosage of 8 mg/mL (excluding IONPs) under identical experimental conditions.

*Stability Test Using PFPE-LP+ and PFS-R1+@IONPs in a Sodium Phosphate Solution*: A solution (pH= 11.13) of 0.5 mg/mL sodium phosphate was prepared using Milli-Q as the matrix. For each sorbent, 5 mL of the prepared sodium phosphate stock solution was added into a glass vial with 10 mg (excluding weight of IONPs based on TGA results) of the linear polymer/crosslinked-resin, to create a final concentration of linear polymer/crosslinked-resin of 2 mg/mL. After sorption for 25 hours on a shaker at room temperature, a large neodymium magnet (N50) was used for recovering the magnetic sorbents for 2 minutes, followed by collection of 500 μL of the residue solution into a 1.5 mL Eppendorf tube for freeze-drying. An equal volume of D_2_O was subsequently added into the tube and the solution was taken for ^1^H NMR spectroscopy analysis.

*Sorption Kinetics of 11 PFAS by PFS-R1+@IONPs and PFS-R2+@IONPs*: For each sorbent, aliquots of 5 mL of the same PFAS stock solution in landfill leachate prepared from the above sorption equilibrium study were added into glass vials containing 10 mg (excluding weight of IONPs based on TGA results) of either PFS-R1+ or PFS-R2+, to create a final concentration of crosslinked-resin of 2 mg/mL. After shaking for a pre-determined time of 5 minutes, 10 minutes, 30 minutes, 1 hour, 2 hours, 4 hours, 8 hours, 14 hours, and 25 hours, magnetic sorbents were recovered using a large neodymium magnet (N50) via magnetic separation for 2 minutes. 1.5 ml of the residue solution was collected, followed by centrifugation at 7000 rpm for 15 minutes. The supernatant (800 μL) was collected for LC-MS/MS analysis. The above experiments were performed in triplicate. Control experiments without addition of any sorbents were also performed under identical conditions.

*Sorption Isotherms of PFBS by PFS-R1+@IONPs, PFA694E, and PFS-R2+@IONPs*: PFBS was selected as a typical PFAS for the isotherm study. Landfill leachate stock solutions with different initial concentrations of PFBS, including 0.1 parts per million (ppm), 0.5 ppm, 2.5 ppm, 5 ppm, 10 ppm, 20 ppm, 50 ppm, 100 ppm, and 250 ppm, were prepared using the original leachate. For each magnetic sorbent, aliquots of 5 mL of the above PFBS stock solution in landfill leachate at each initial concentration were individually added into glass vials containing 10 mg (excluding weight of IONPs based on TGA results) of either PFS-R1+ or PFS-R2+, to create a final concentration of crosslinked-resin of 2 mg/mL. For PFA694E, aliquots of 5 mL of PFBS stock solution at each initial concentration were individually added into vials containing 10 mg of the sorbent. After shaking for 27 hours, 1.5 mL of the residue solution was collected after magnetic separation for 2 minutes using a large neodymium magnet (N50), followed by centrifugation at 7000 rpm for 15 minutes. Direct centrifugation was performed for PFA694E. The supernatant (800 μL) was collected and diluted using deionized water for LC-MS/MS analysis. The above experiments were performed in triplicate. Control experiments without addition of any sorbents were also performed under identical conditions.

*The Individual Sorption Capacity of Five Short-Chain PFAS and GenX by PFS-R1+@IONPs and PFS-R2+@IONPs*: Stock solutions that individually contain five short-chain PFAS, including PFBA, PFPeA, PFHxA, PFHpA, and PFBS, along with GenX, were prepared at an initial concentration of 250 ppm using original landfill leachate as the matrix. For each magnetic sorbent, aliquots of 5 mL of the above PFAS stock solution in landfill leachate were individually added into glass vials containing 5 mg (excluding weight of IONPs based on TGA results) of either PFS-R1+ or PFS-R2+, and created a final concentration of crosslinked-resin of 1 mg/mL. After shaking for 27 hours, 1.5 mL of the residue solution was collected after magnetic separation for 2 minutes using a large neodymium magnet (N50), followed by centrifugation at 7000 rpm for 15 minutes. The supernatant (500 μL) for each sample was collected for ^19^F NMR spectroscopy analysis. Control experiments without addition of any sorbents were also performed under identical conditions.

*Batch Experiments of Removing 11 PFAS at Environmentally Relevant Concentrations Using Different Types of Sorbents*: PFS-R1+@IONPs and PFS-R2+@IONPs, along with four commercially available sorbents including PFA694E, GAC, IRA-410, and IRA-402 were used for batch experiments of removing 11 PFAS at environmentally relevant concentrations. 11 PFAS, including PFBA, PFPeA, PFHxA, PFHpA, PFOA, PFNA, PFDA, PFBS, PFHxS PFOS, and GenX, were manully spiked into the original landfill leachate and created initial concentrations of 22 ppb for PFBS, and 1 to 5 ppb for the remaining PFAS. For each sorbent, 5 mL of the above prepared PFAS stock solution at environmentally relevant concentrations in landfill leachate was added into a glass vial with 20 mg (excluding weight of IONPs for PFS-R1+@IONPs and PFS-R2+@IONPs based on TGA results) of the crosslinked-resin/commercailly available sorbent, to create a final concentration of crosslinked-resin/commercailly available sorbent of 4 mg/mL. After sorption for either 2 or 25 hours on a shaker at room temperature, a large neodymium magnet (N50) was used for recovering the magnetic sorbents for 2 minutes, followed by collection of 2 mL of the residue solution and centridugation at 7000 rpm for 15 minutes. For each sample treated by magnetic sorbents, 800 μL of the supernatant was carefully collected for subsequent LC-MS/MS analysis, and another 800 μL of the supernantant was collected for UV-Vis experiments. For the four commercailly availbale sorbents, direct centrifugation at 7000 rpm for 15 minutes was performed. The above experiments were performed in triplicate. Control experiments without addition of any sorbents were also performed under identical conditions. For UV-Vis experiments, the solutions from the triplicate trials treated by a single magnetic sorbent with the same treatment duration were combined before analysis.

*Batch Experiments of Removing 11 PFAS at Environmentally Relevant Concentrations Using PFS-R1+@IONPs and PFS-R2+@IONPs in Drinking Water and Compost Leachate*: PFS-R1+@IONPs and PFS-R2+@IONPs were evaluated in batch experiments for the removal of 11 PFAS at environmentally relevant concentrations. The PFAS (PFBA, PFPeA, PFHxA, PFHpA, PFOA, PFNA, PFDA, PFBS, PFHxS, PFOS, and GenX) were manually spiked into original drinking water (tap water from Brisbane) and compost leachate to achieve initial concentrations of ~1 ppb for each compound in both matrices. For each sorbent, 5 mL of the spiked PFAS solution (either drinking water or compost leachate) was added to a glass vial containing 20 mg (excluding the weight of IONPs, as determined by TGA) of either PFS-R1+@IONPs or PFS-R2+@IONPs, yielding a final sorbent concentration of 4 mg/mL in each vial. After sorption for 2 hours on a shaker at room temperature, a large neodymium magnet (N50) was used for recovering the magnetic sorbents for 2 minutes, followed by collection of 1.5 mL of the residue solution and centridugation at 7000 rpm for 15 minutes. 800 μL of the supernatant was carefully collected for subsequent LC-MS/MS analysis. Control experiments without addition of any sorbents were also performed under identical conditions.

*Molecular Dynamics (MD) Simulations of Fluorous and Electrostatic Interactions Between Polymer Sorbents and PFAS in Complex Aqueous Media*: MD simulations were performed using the LAMMPS package to study the sorption behavior of two functionalized polymers, which are simplified models representing PFS-R2+ and PFS-R1+, toward PFAS and humic acid (HA) in aqueous environments.^1^ The simulation box was constructed by *PACKMOL* for each system, and contained 20 polymer chains assembled into a spherical aggregate positioned at the center, surrounded by 50 PFAS molecules, 25 HA molecules, and 400,000 water molecules described by the SPC model.^2^ The HA molecules were modeled using the Temple-Northeastern-Birmingham (TNB) surrogate structure to capture their natural aromatic and hydrophilic features **(Figure S24)**.^3,4^

Both polymers were modeled as random copolymers. As shown in **Figure S25** and **Figure S26**, the simplified model for PFS-R2+ was constructed from 20 vinylbenzyltrimethylammonium chloride (VBTAC, quaternary ammonium group) units and 10 PFS units, while the simplified model for PFS-R1+ was constructed from 10 VBTAC and 20 PFS units. Both polymer topologies were built using Moltemplate, with force field parameters assigned according to the OPLS-AA force field, including definitions of atom types, bonded interactions, and dihedrals.^5,6^ Atomic partial charges were derived from electrostatic potentials using RESP fitting (**Table S9-S12**), based on DFT calculations at the M06-2X/def2-TZVP level with Gaussian 16.^7-11^ The resulting charges were incorporated into the LAMMPS input via Moltemplate.^12^

Energy minimization was first performed to remove steric clashes, followed by a 1 ns NPT equilibration at 300 K and 1 atm using the Nosé-Hoover thermostat and barostat. Subsequently, production runs of 20 ns were carried out under the NVT ensemble with a 1 fs time step. Nonbonded interactions were modeled using a 14 Å cutoff for both Lennard-Jones and Coulomb interactions, with long-range electrostatics handled using the particle-particle particle-mesh (PPPM) solver at a relative accuracy of 10^-4^.

A molecule (either PFAS or HA) was defined as sorbed if any of its atoms approached within 4.5 Å of any atom on the polymer in at least one trajectory frame, reflecting a typical first solvation shell distance.^13,14^ The trajectory analysis and visualization were conducted using VMD with custom Tcl scripts to detect sorption events, highlight relevant molecular interactions, and generate structural snapshots for graphical representation.^15^ All visualization and sorption scripts are publicly available at [https:/github.com/Jeo-wq/Adsorption_script](https://github.com/Jeo-wq/Adsorption_script).

*A Compact and Portable Device for Removal of 11 PFAS at Environmentally Relevant Concentrations and the Subsequent Sorbent Regeneration Using PFS-R2+@IONPs*: In the device experiment, 624 mg of PFS-R2+@IONPs were added to a 100 mL glass beaker. During a single cycle, one channel of a four-channel peristaltic pump was activated to transfer 100 mL of PFAS-contaminated landfill leachate (the same stock leachate used in the above batch experiments) into the beaker, resulting in a concentration of 4 mg/mL of the crosslinked resin PFS-R2+ (excluding the weight of IONPs, as determined by TGA). The pump channel was then turned off, and mechanical stirring was initiated at 350 rpm for 2 hours. After complete sorption, the mechanical stirring was stopped, and an electromagnet (5 V, 20 kg suction) located at the bottom of the beaker was activated for 2 minutes to recover the PFS-R2+@IONPs. The second channel of the peristaltic pump was then turned on to remove the purified landfill leachate solution, followed by addition of 50 mL of pure methanolic salt solution (methanol+1% NaCl), with the second channel turned off and the third channel turned on. Afterwards, the electromagnet and third pump channel were turned off, and mechanical stirring was resumed to initiate the regeneration process for 30 minutes. Upon complete PFAS release, the mechanical stirring was stopped, and the electromagnet was turned on for 2 minutes to recover the sorbent. Finally, the eluted PFAS in the methanolic salt solution was collected by turning on the fourth channel of the peristaltic pump. The number of cycles was extended to five. The untreated landfill leachate, the landfill leachate treated with PFS-R2+@IONPs after each cycle, and the eluted PFAS solution after each cycle were collected for LC-MS/MS analysis. To investigate the concentration factor during regeneration, the same sorption process was performed using the device, but with 20 mL and 10 mL of regenerant.

*Instruments*

**Solution Nuclear Magnetic Resonance (NMR)**

^1^H NMR spectra of polymer solutions in either CDCl_3_ or D_2_O were acquired on a Bruker Avance 500 MHz spectrometer at 25 °C. A 90° pulse width 14 μs, relaxation delay 1 s, acquisition time 3.9 s, and 64 scans were used in all measurements.

^19^F NMR spectra were acquired on a Bruker Avance 500 MHz spectrometer with either CDCl_3_ or aqueous solution as solvent. Coaxial inserts containing 99% D_2_O and 1% of 2, 2, 2-trifluoroethanol (TFE, *v*/*v*) as the internal standard were used for quantifications of the individual capacity of six short-chain PFAS by PFS-R1+@IONPs and PFS-R2+@IONPs in aqueous solution. Spectra for the sample in CDCl_3_ were measured under the following conditions: 90° pulse width 15 μs, relaxation delay 2 s, acquisition time 0.46 s, and 128 scans. Spectra for samples from individual capacity analysis were measured under the following conditions: 90° pulse width 15 µs, relaxation delay 5 s, acquisition time 0.46 s, and 256 scans.

**Scanning Electron Microscopy-Energy Dispersive Spectroscopy (SEM-EDS)**

All SEM samples were mounted on an aluminium sample holder by carbon tape. The samples were sputter-coated with 35 nm carbon using Quorum Q150T Carbon Coater. SEM images and EDS spectrums were obtained from field emission SEM with a hot (Schottky) electron gun (JEOL JSM-7800F SEM EBSD) equipped with an Oxford HKL EBSD system, with accelerating voltage and probe current set to be 6 kV and 12 respectively.

**^13^C Solid State (SS) NMR Spectroscopy**

^13^C SS NMR experiments were performed on a Bruker Avance III spectrometer with a 300 MHz magnet equipped with a 4 mm double air bearing, magic angle spinning probe. The powdered samples were placed in zirconia rotor with a Kel-F cap and spinned at 5 kHz. ^13^C spectra were recorded with CPMAS and CPTOSS pulse sequences. The ramped cross-polarization time was 2 ms, high-power decoupling employed tppm15 sequence with 100 kHz proton power. Relaxation delay was 3 s and acquisition time 49 ms. Spectra were referenced to adamantane.

**Fourier Transform Infrared Spectroscopy (FTIR)**

FTIR spectra were recorded on a Thermo Scientific Nicolet 5700 FTIR spectrometer equipped with a smart orbit diamond ATR unit. The wavenumber range was 4000-400 cm^-1^ and the resolution was 4 cm^-1^. The number of scans was set as 64.

**Thermogravimetric Analysis (TGA)**

Weight percentage of either bare magnetic IONPs or polymeric materials@IONPs before and after quaternization were determined using a STARe thermogravimetric analyzer (MettlerToledo, LLC, Columbus, OH). Samples were heated from 40 to 600 °C at a rate of 10 °C min^-1^.

**Zeta Potential Measurement**

The Zeta Potential was measured on the Nanoseries Zetasizer (Malvern, UK) instrument using a DTS1070 folded capillary zeta cell. 800 µL of the sample solution was used for the measurement. The number of runs per measurement was set as between 20 to 50.

**Magnetic Hysteresis Measurement**

Magnetic hysteresis measurements were performed on a Quantum Design MPMS3 SQUID magnetometer measuring at 300 K with an applied field from -3 to 3 T and an average sweep rate of 20 Oe/s. The powder samples were loaded into plastic capsules suspended on a brass holder. The raw data was weight corrected to yield emu per gram and data was shape-corrected based on correction factors detailed by Quantum Design.

**Liquid Chromatography with Tandem Mass Spectrometry (LC-MS/MS)**

The samples were analyzed with a Shimadzu Nexera LC-40 coupled to a SCIEX Triple Quad 7500 system. Before analyzing, all the samples were spiked with mass labelled internal standards at a final concentration of 4 ng/mL. Chromatographic separation was achieved at 50 °C on a Phenomenex Kinetex EVO C18 2.6 µm, 100 x 2.1 m column (Part number 00D-4725-AN) equipped with a guard cartridge (Part number AJ0-9298) using gradient elution of mobile phase A (1 % methanol/ 8mM ammonium acetate in water) and mobile phase B (95 % methanol/ 8mM ammonium acetate in water). The flow rate was set at 0.4 mL/min with an injection volume of 5 µL. To mitigate interference from PFASs contamination in the UHPLC solvents and instrument system, a delay column Kinetex EVO C18, 5 µm, 30 x 2.1 mm) was installed between the solvent mixer and autosampler. The mass spectrometer was operated in multiple reaction monitoring (MRM) mode in negative ion mode and details of the MRM used for both analytes and internal standards are presented in **Table S13**. The Optiflow ion source with an electrospray ionisation was used. The source and MS parameters were as follows: source temperature=450 °C, curtain gas=42 psi, ion source gas 1=50 psi, ion source gas 2=70 psi, spray voltage=1500 V, CAD gas=9.

The removal efficiency was calculated as RE (%)=[(C_0_ - Cs)/C_0_] × 100 %, where C_0_ and Cs are the concentrations of PFAS before and after treatment with sorbents, respectively.

The limits of detection (LODs) for PFAS by direct injection were 0.001 ppb for all 11 compounds. The method of direction injection was applied for all the samples analyzed in this work. For PFAS concentrations below the LODs after treatment by sorbents, LODs were used for calculations of removal efficiency values of PFAS.

**Ultraviolet-Visible (UV-Vis) Spectrophotometer**

The UV–Vis spectra were acquired on a Shimadzu UV-2600 spectrophotometer. The transmittance of the landfill leachate solution before and after treatment using either PFS-R1+@IONPs or PFS-R2+@IONPs was recorded from 500 to 850 nm at room temperature using a polystyrene cuvette. Milli-Q was used as the reference sample.

*Statistical Analysis*

PFAS removal data were analyzed using either GraphPad Prism 10 or Origin 2024b. Mean values with standard deviations are reported for experiments conducted in triplicate. Only mean values are reported for single-run experiments.

**Table S1**. Compositions for all four polymeric sorbents prepared in this work.

|  | PFS-LP | | PFS-R1 | | Sty-R | | PFS-R2 | |
| --- | --- | --- | --- | --- | --- | --- | --- | --- |
|  | Feeding Ratios | | | | | | | |
|  | Wt  (%) | Mol (%) | Wt  (%) | Mol (%) | Wt  (%) | Mol (%) | Wt  (%) | Mol (%) |
| PFS/Styrene | 28.9 | 28.6 | 27.3 | 26.7 | 27.3 | 40.4 | 11.9 | 11.4 |
| VBC | 12.7 | 14.3 | 11.9 | 13.3 | 11.9 | 10.8 | 27.3 | 29.9 |
| TEVS | 58.4 | 57.1 | 55.1 | 53.3 | 55.1 | 43.4 | 55.1 | 52.2 |
| DVB | - | - | 5.7 | 6.7 | 5.7 | 5.4 | 5.7 | 6.5 |

**
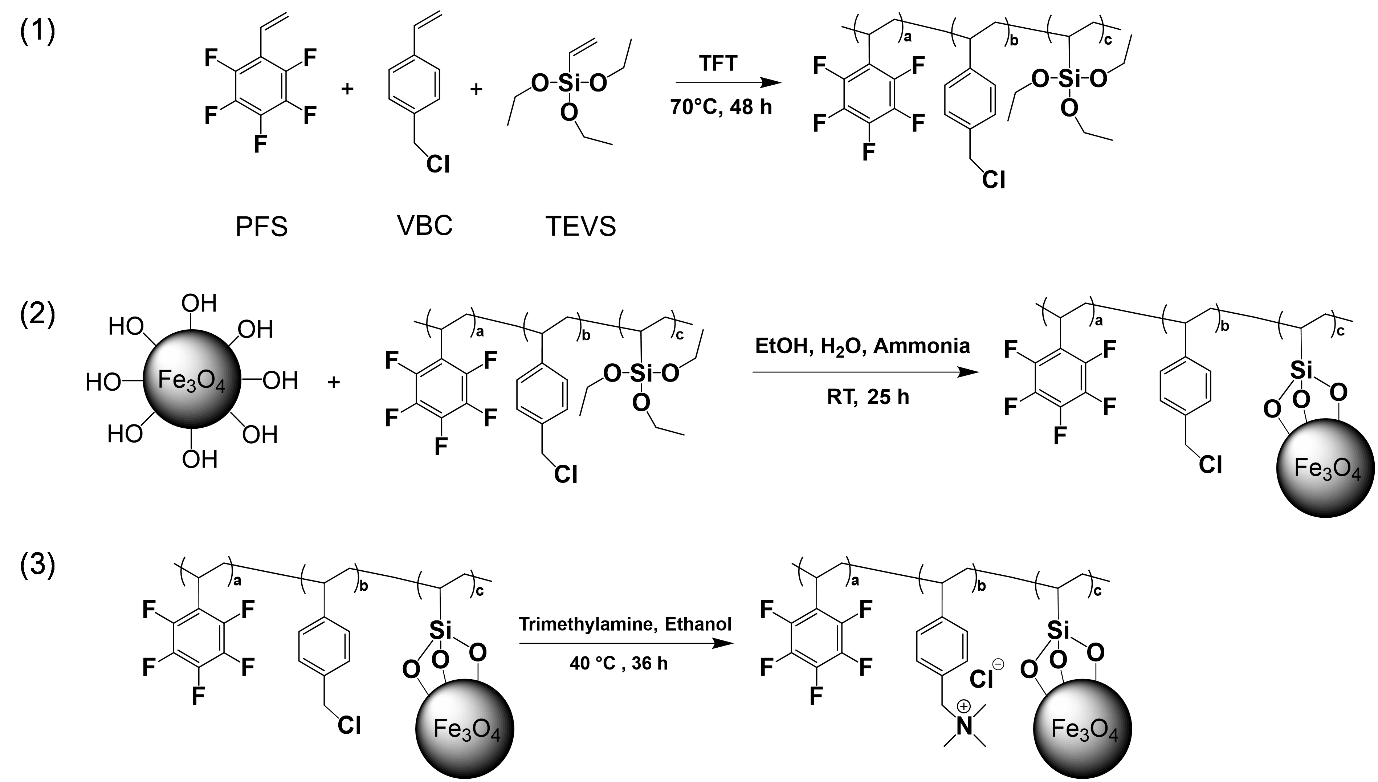
**

**Scheme S1.** Synthetic scheme for producing PFS-LP+@IONPs.

**
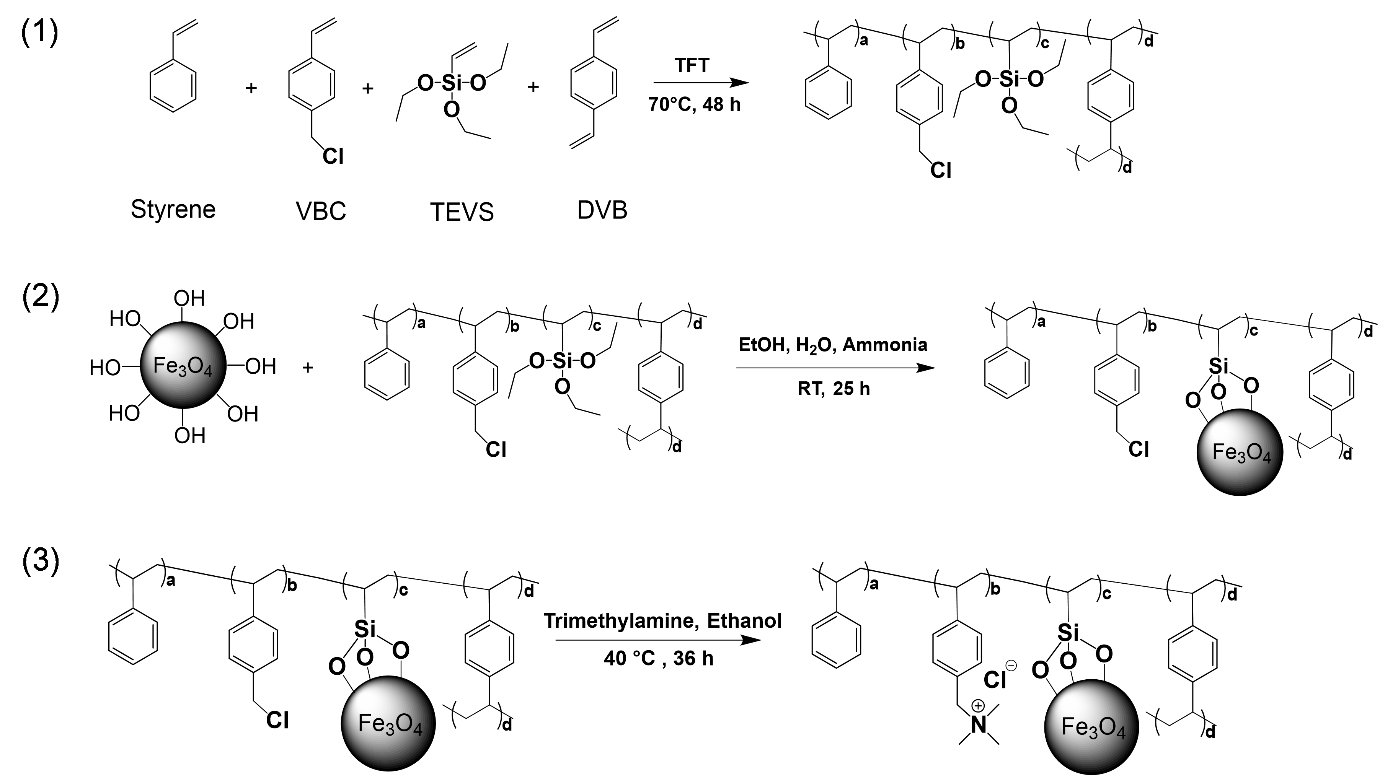
**

**Scheme S2.** Synthetic scheme for producing Sty-R+@IONPs.

**
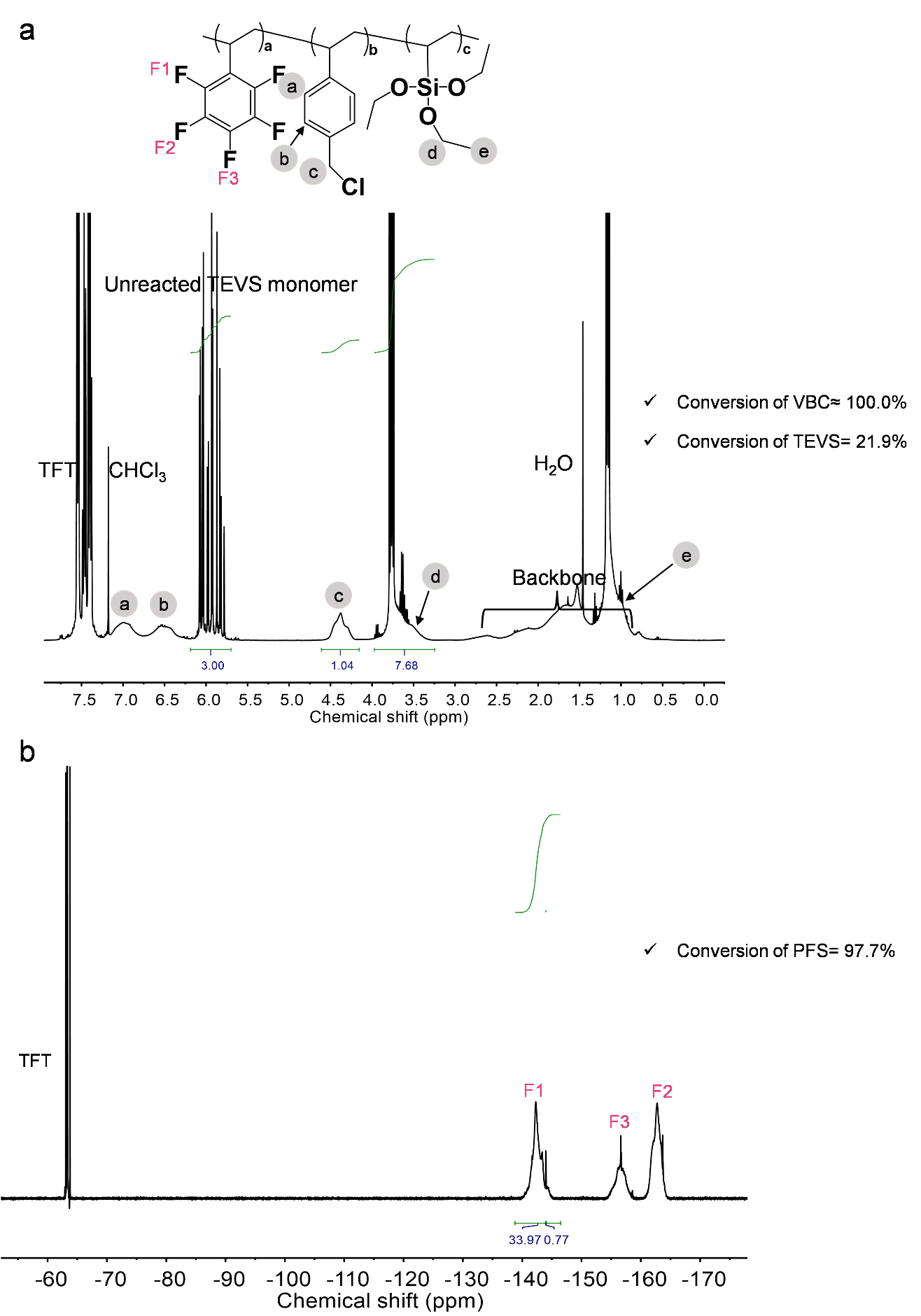
**

**Figure S1**. ^1^H a) and ^19^F b) NMR of PFS-LP in CDCl_3_ before purification.

**
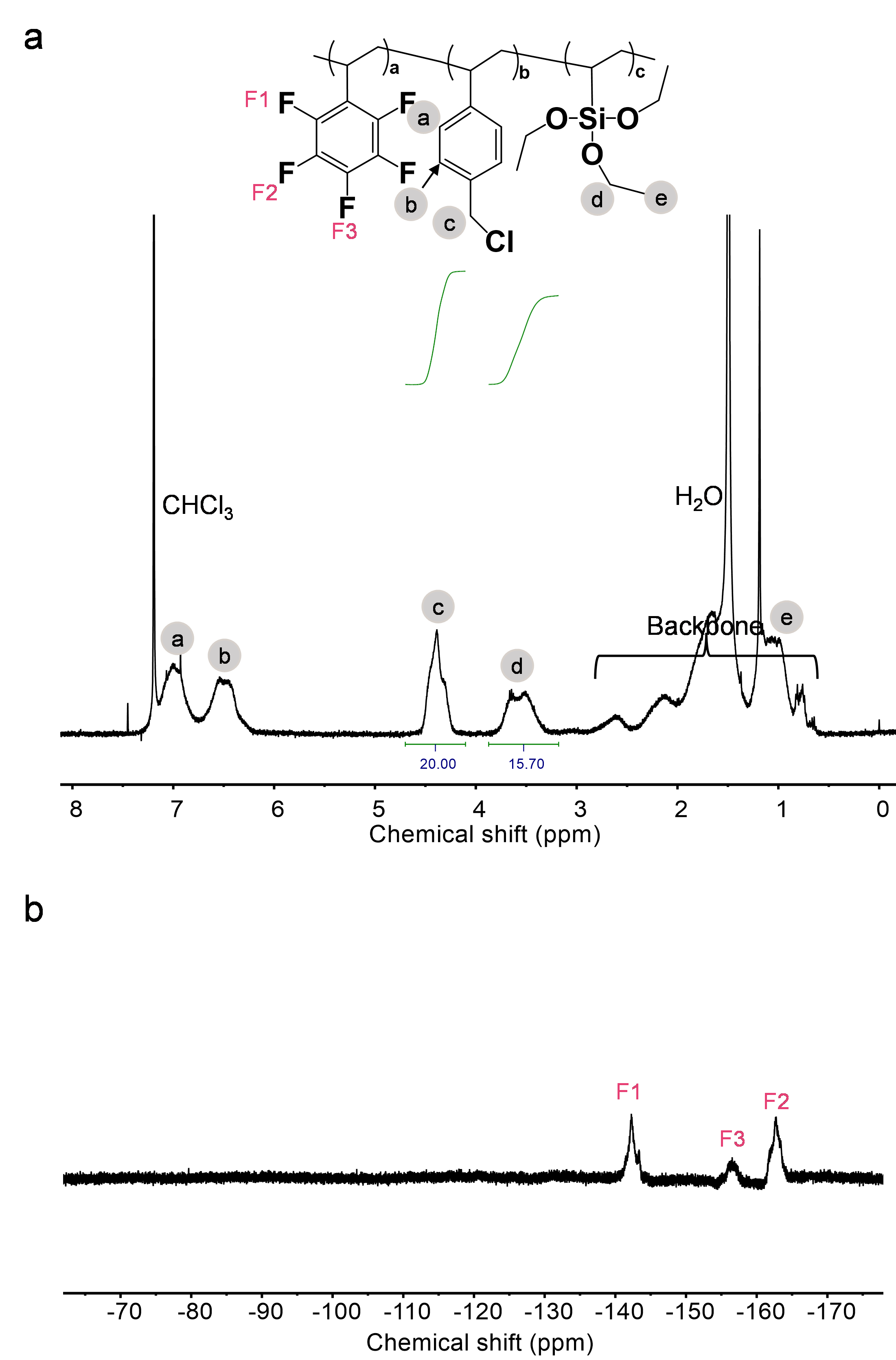
**

**Figure S2**. ^1^H a) and ^19^F b) NMR of PFS-LP in CDCl_3_ after purification.


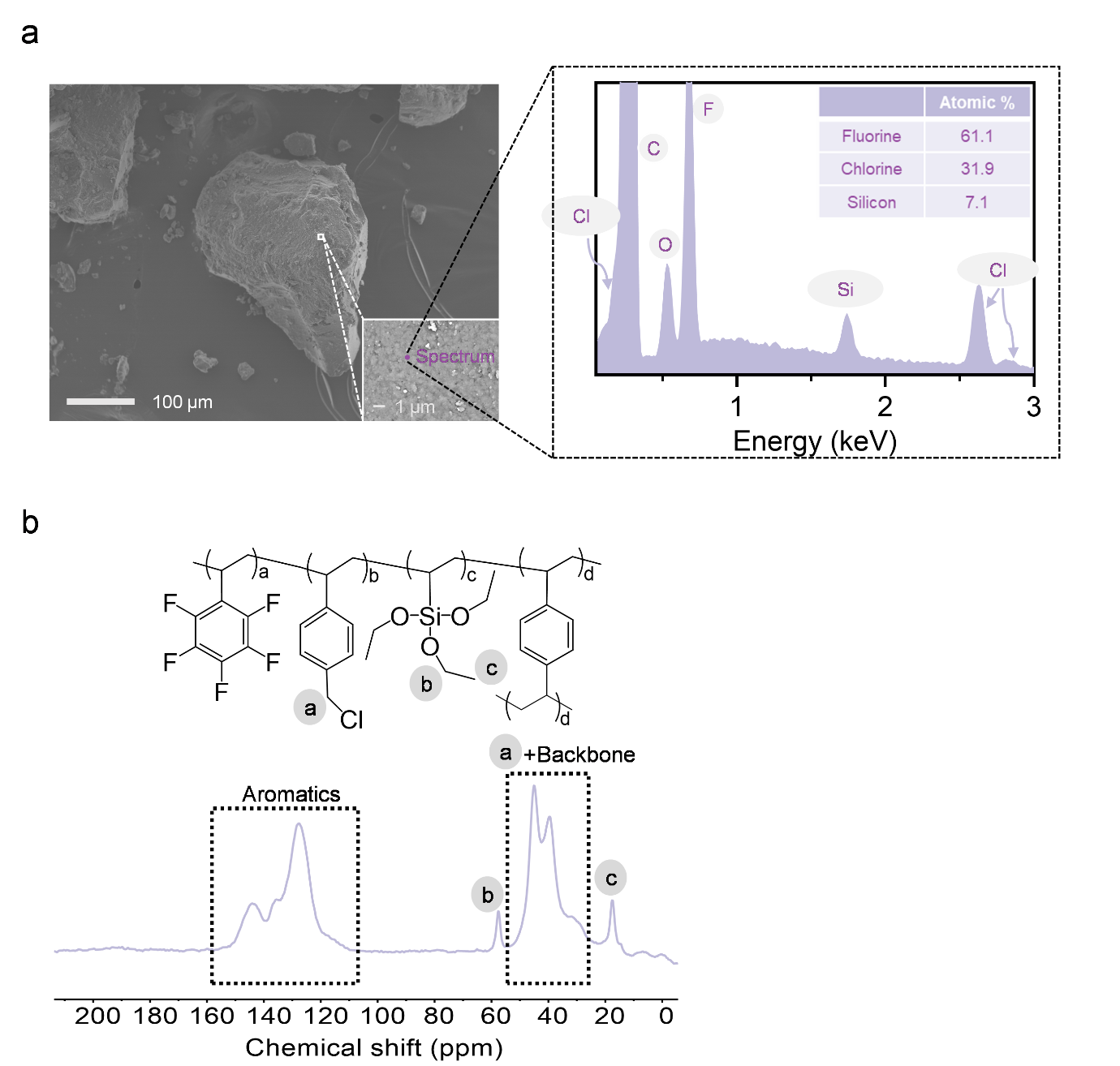


**Figure S3.** Characterizations of PFS-R2 before grafting on IONPs. a), EDS spectrum of PFS-R2; b), ^13^C SS NMR of PFS-R2.

**
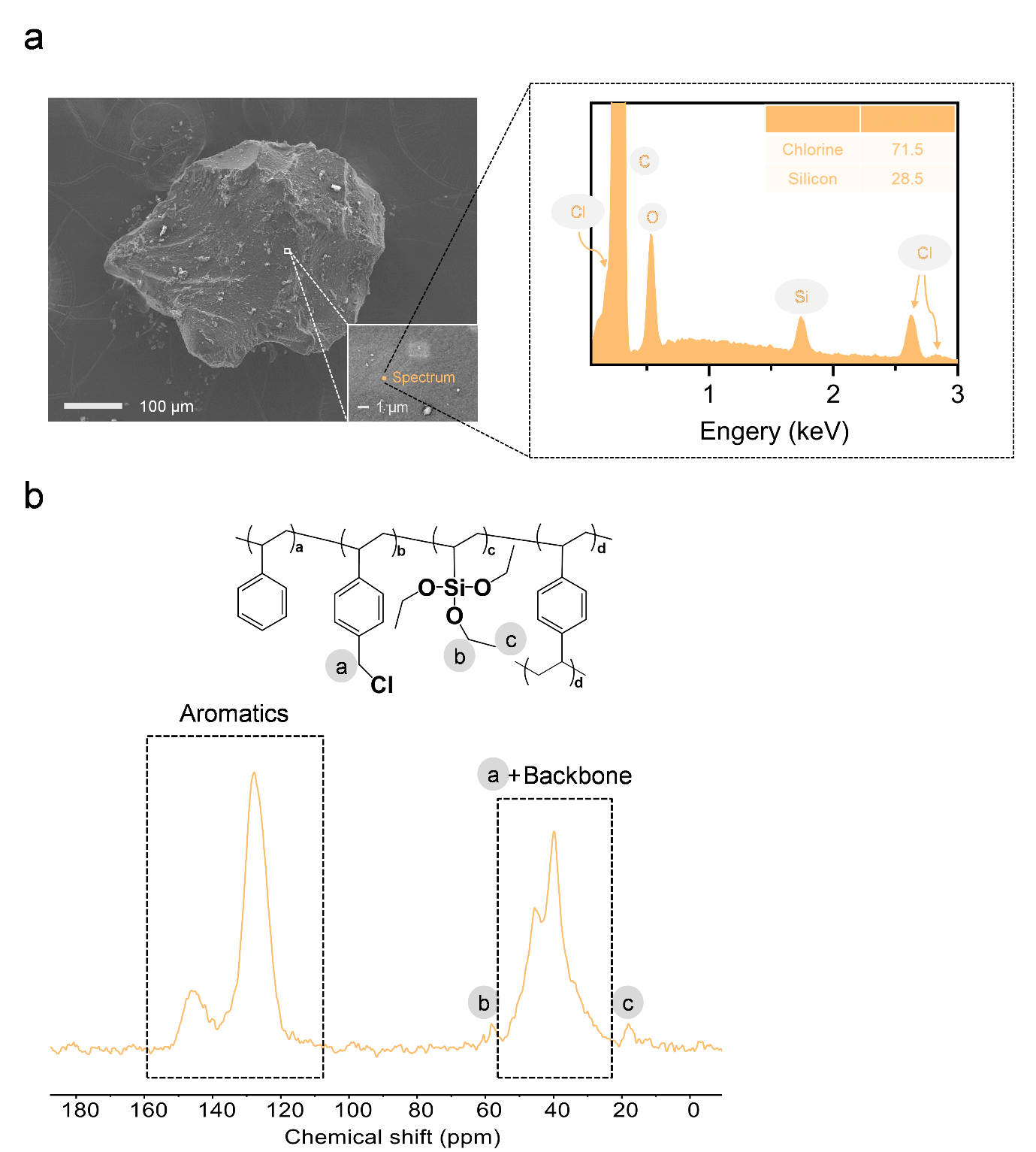
**

**Figure S4.** Characterizations of Sty-R before grafting on IONPs. a), EDS spectrum of Sty-R; b), ^13^C SS NMR of Sty-R.

**
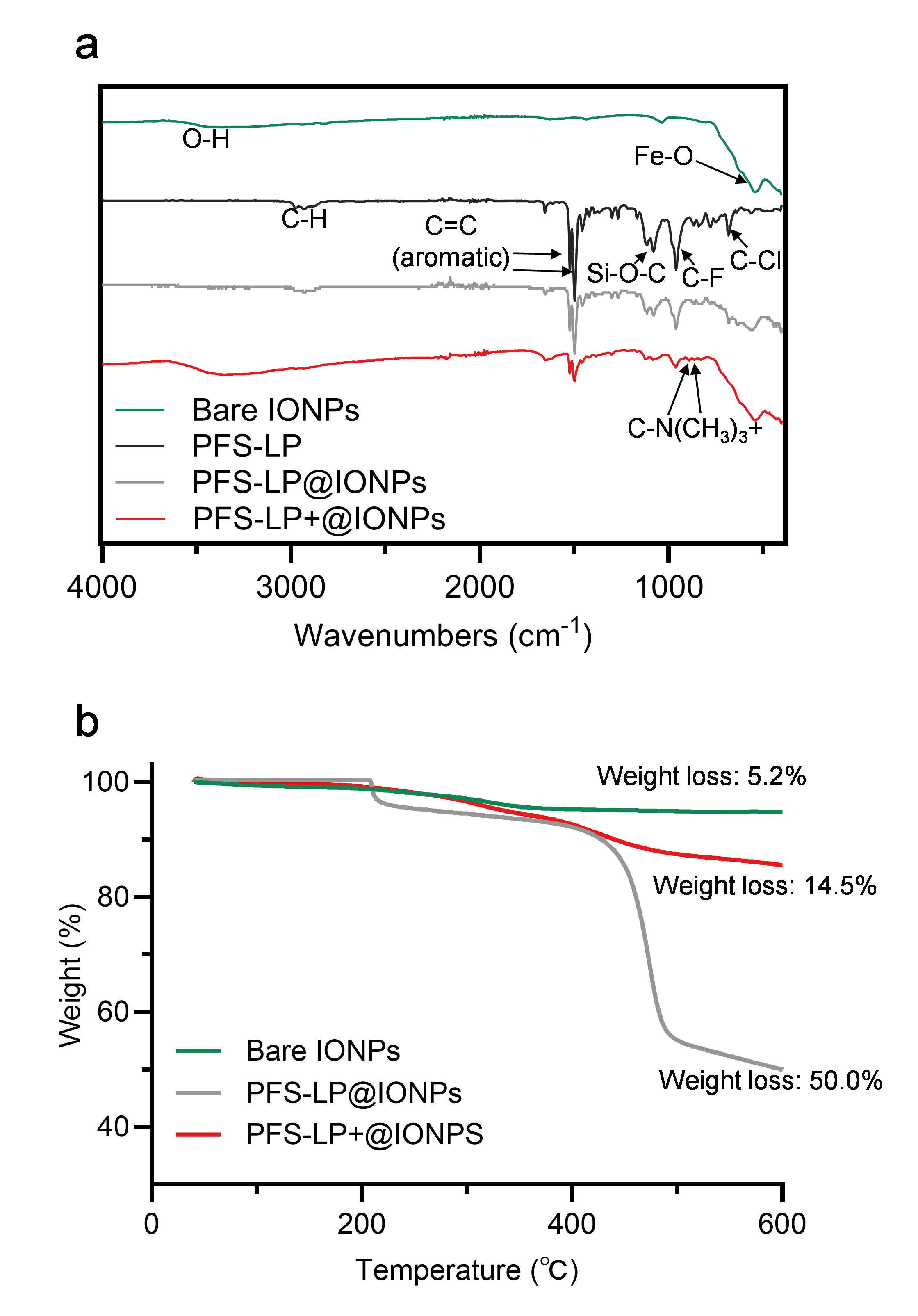
**

**Figure S5.** FTIR a) and TGA b) characterizations for PFS-LP-related magnetic polymeric materials.

**
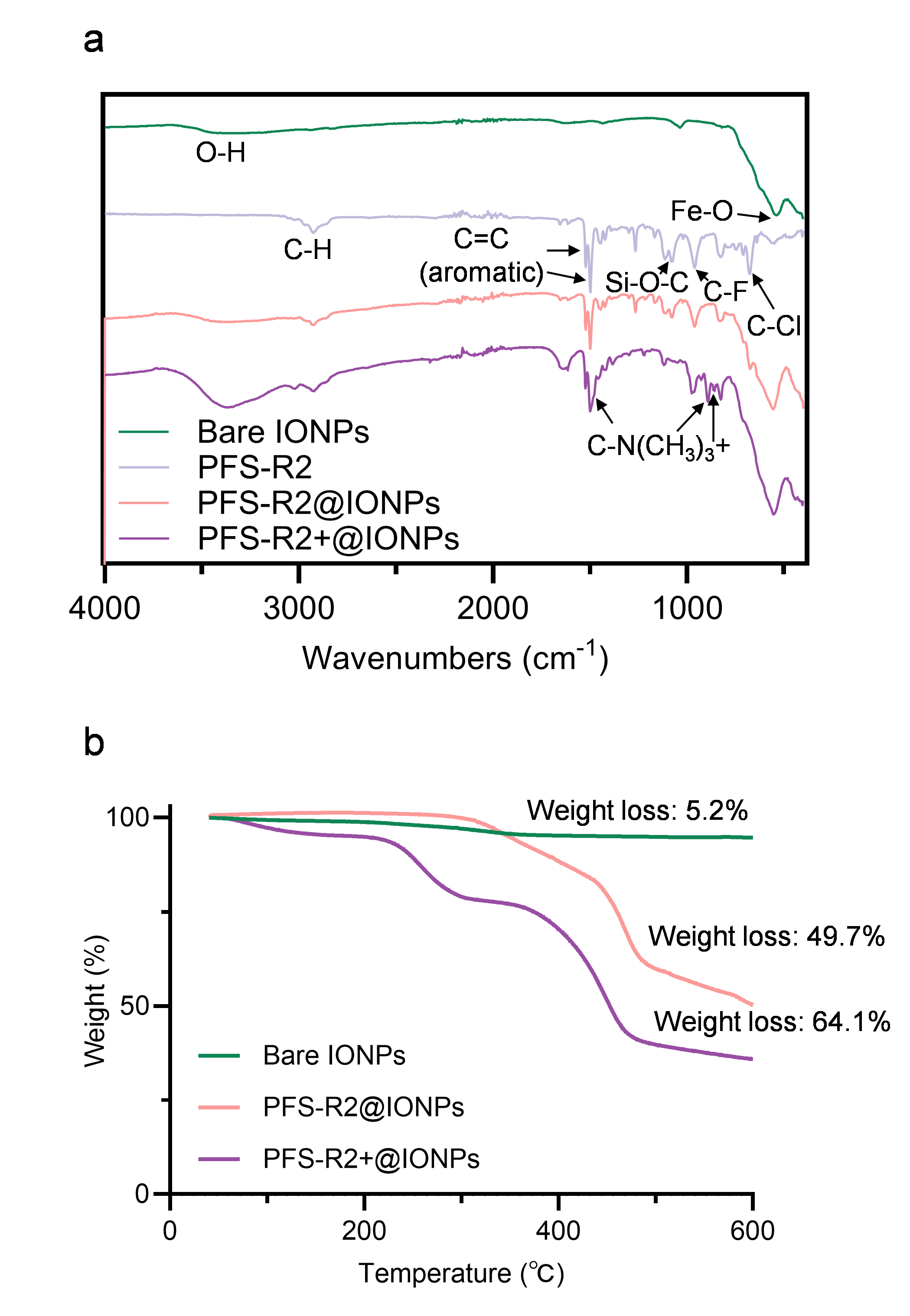
**

**Figure S6.** FTIR a) and TGA b) characterizations for PFS-R2-related magnetic polymeric materials.

**
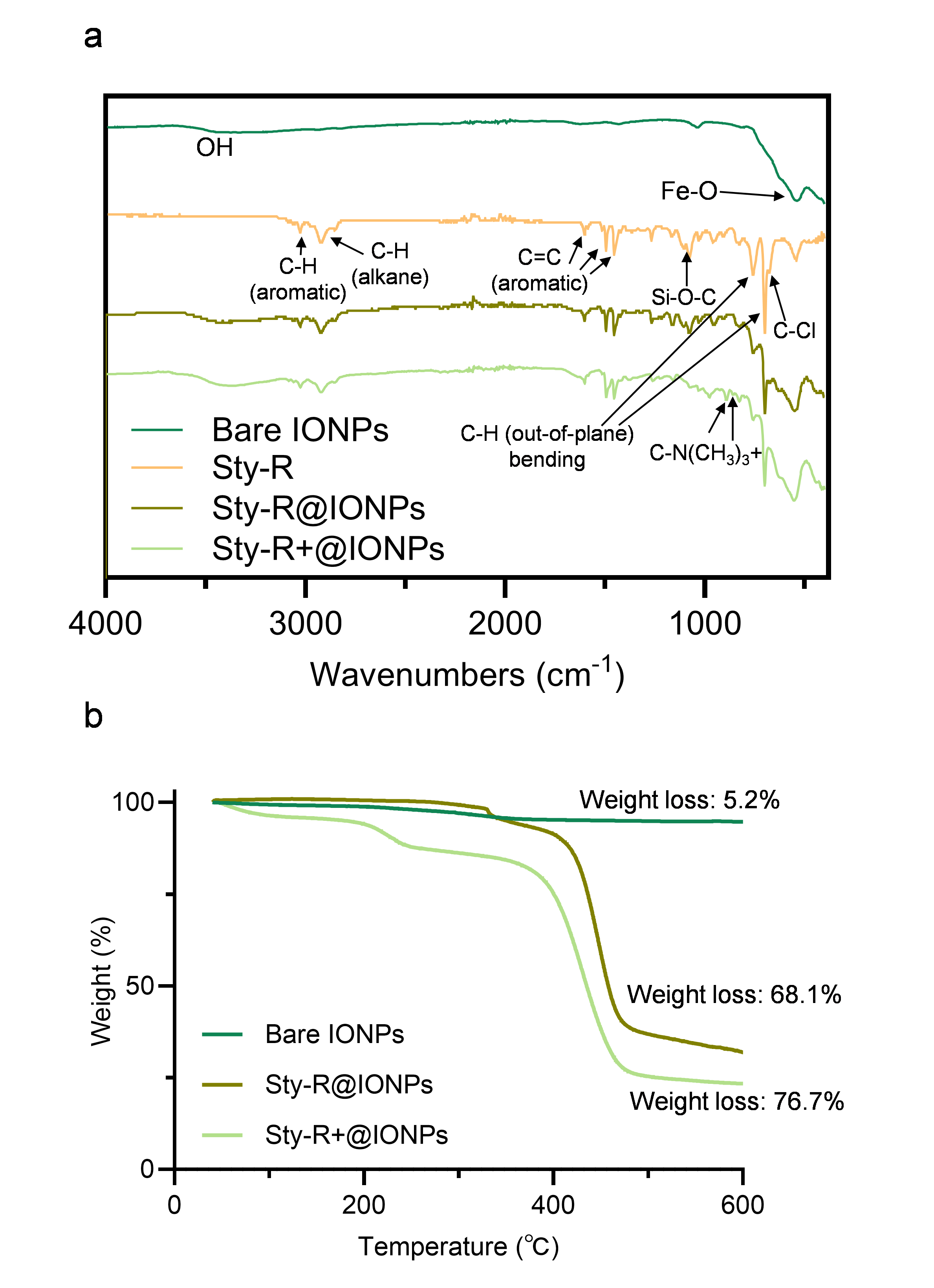
**

**Figure S7.** FTIR a) and TGA b) characterizations for Sty-R-related magnetic polymeric materials.

**
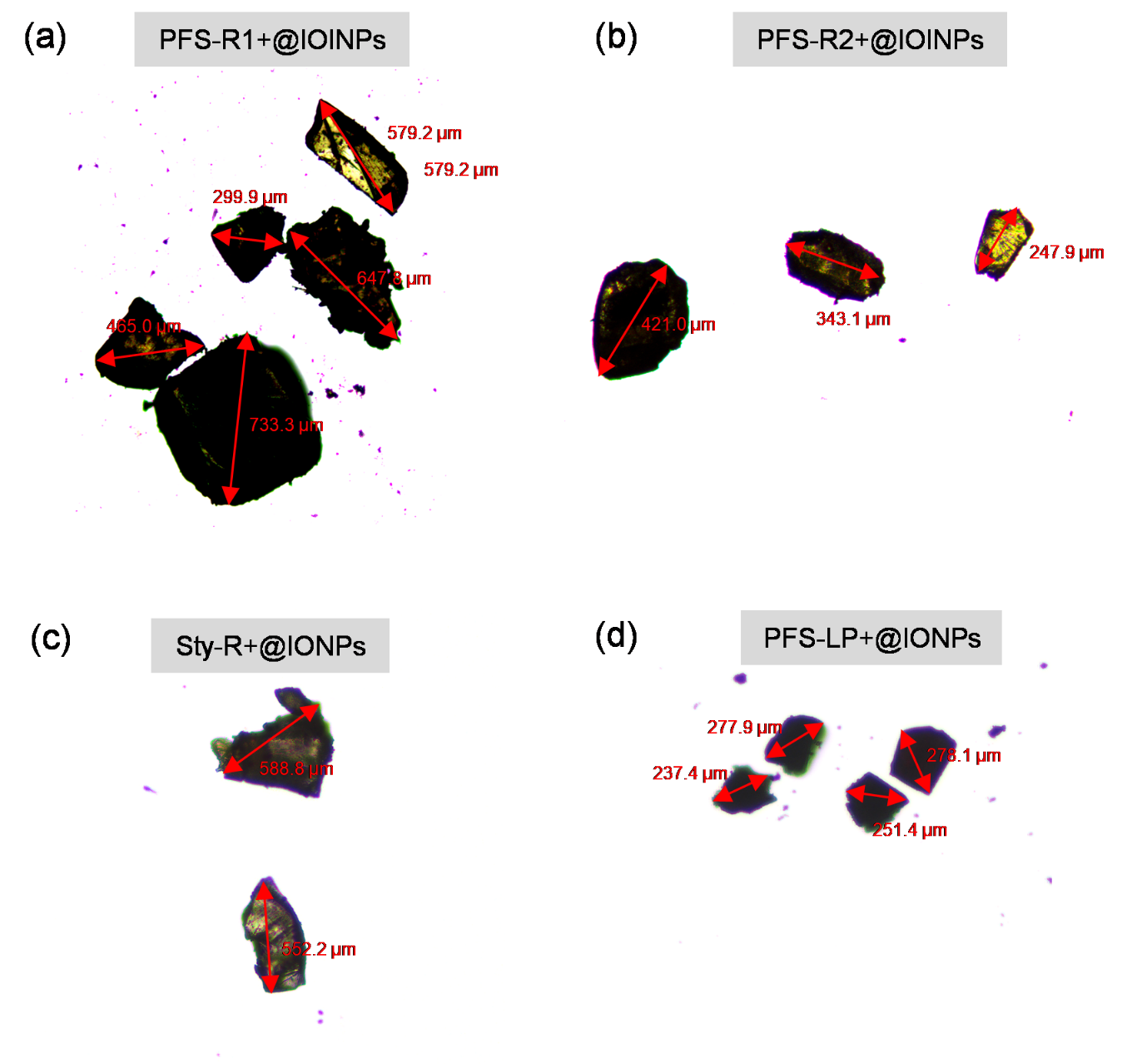
**

**Figure S8.** Photos of four prepared magnetic polymeric sorbents after quaternization taken using an optical microscope.

**
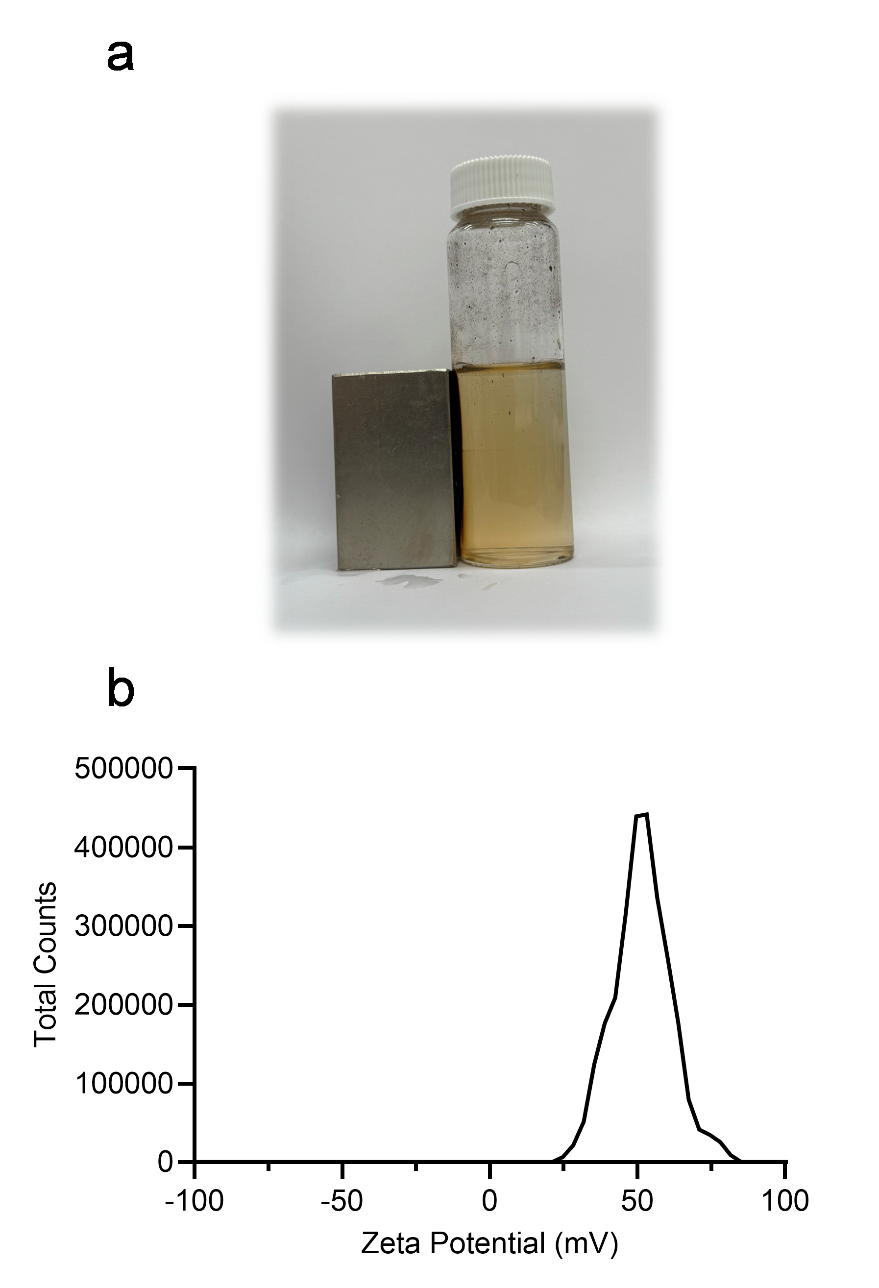
**

**Figure S9.** Characterization of the water solution during the purification process of PFS-LP+ after magnetic separation. a) A picture demonstrating the magnetic separation of PFS-LP+ after 10 minutes; b) Zeta potential of the water solution collected from a).


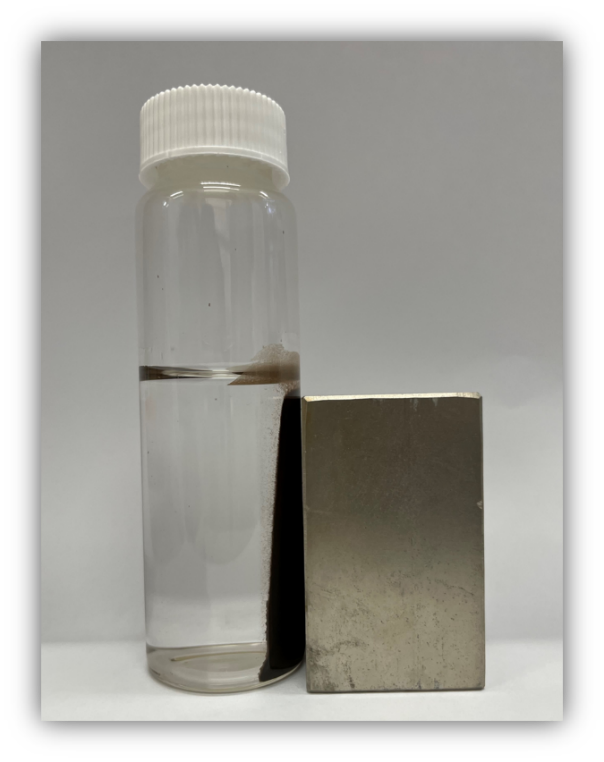


**Figure S10.** A representative image showing the magnetic separation of PFS-R1+@IONPs in water solution after 2 minutes.

**Table S2.** Concentrations (in ppb) of 11 PFAS in original landfill leachate (before manually spiking 11 PFAS).

| **PFAS Name** | **Concentrations (ppb)** |
| --- | --- |
| PFBA | 1.88 |
| PFPeA | 1.16 |
| PFHxA | 3.43 |
| PFHpA | 0.58 |
| PFOA | 1.23 |
| PFNA | 0.06 |
| PFDA | 0.05 |
| PFBS | 21.21 |
| PFHxS | 0.71 |
| PFOS | 0.21 |
| GenX | 0.01 |


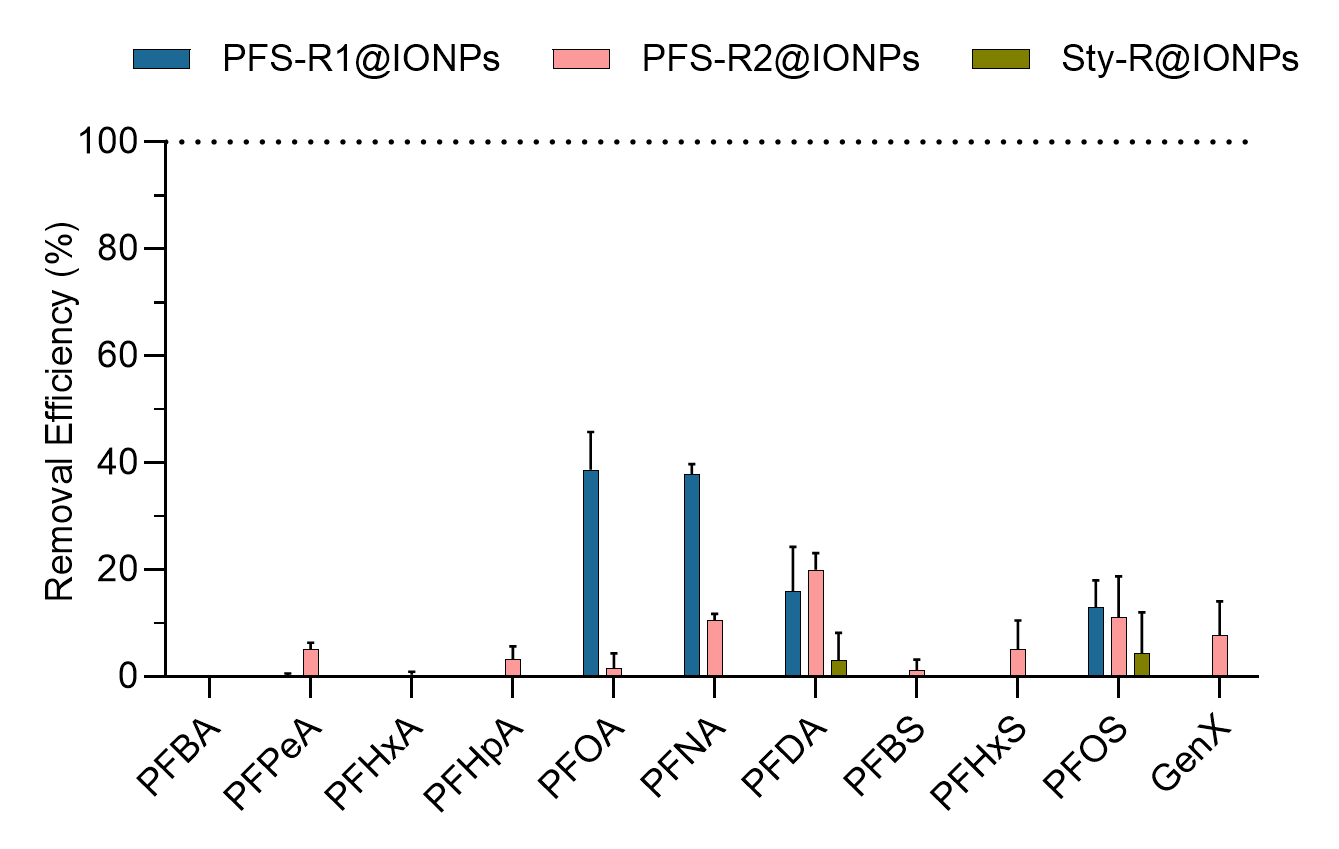


**Figure S11.** Equilibrium sorption of the 11 PFAS by three prepared magnetic polymeric sorbents before quaternization. Sorbent: 2 mg/mL (excluding IONPs). Each PFAS initial concentration: 44-118 ppb. Treatment duration: 25 hours. pH= 8.24. The results are the average of three replicates, and one standard deviation is shown.

**Table S3.** Compositions of original landfill leachate before PFAS spiking.

|  | Compositions | Concentration (mg/L) |
| --- | --- | --- |
| Total Carbon | Total organic carbon (TOC) | 3211 |
|  | Total inorganic carbon (TIC) | 1815 |
|  | Total carbon (TC) | 5026 |
| Element | Al | 0.98 |
|  | As | 0.97 |
|  | B | 5.53 |
|  | Ba | 0.40 |
|  | Ca | 50.10 |
|  | Cd | 0.002 |
|  | Co | 0.06 |
|  | Cr | 1.71 |
|  | Cu | 0.02 |
|  | Fe | 5.37 |
|  | K | 1361.60 |
|  | Mg | 52.80 |
|  | Mn | 0.10 |
|  | Mo | 0.01 |
|  | Na | 3007.40 |
|  | Ni | 0.25 |
|  | P | 20.03 |
|  | Pb | 0.01 |
|  | S | 71.30 |
|  | Se | 0.02 |
|  | Si | 50.13 |
|  | Sr | 0.92 |
|  | V | 0.05 |
|  | Zn | 0.53 |

Note: The concentration of each element was measured using inductively coupled plasma optical emission spectroscopy (ICP-OES).


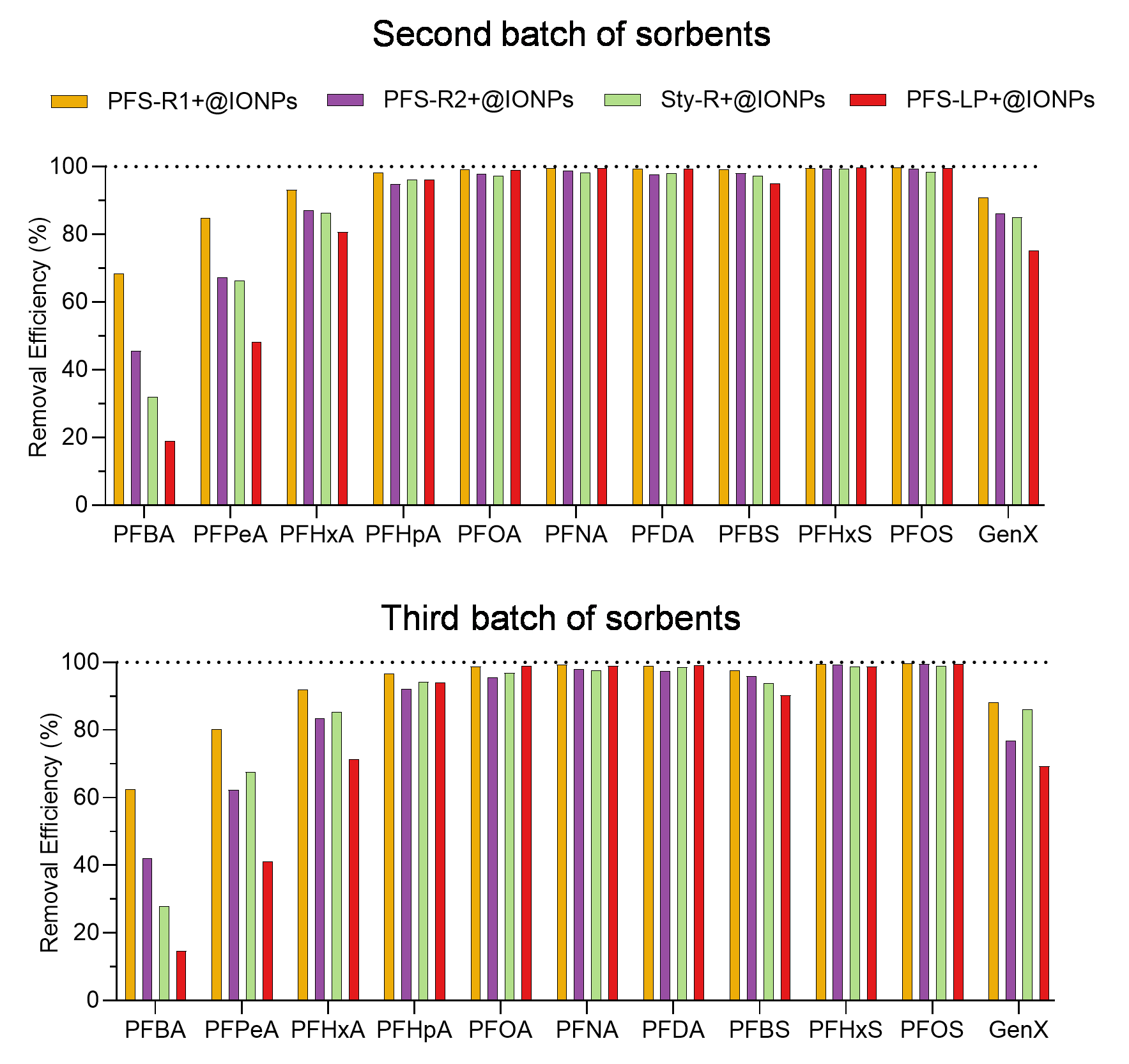


**Figure S12.** Removal 11 PFAS from landfill leachate using four magnetic polymeric sorbents prepared from two additional batches. Sorbent: 2 mg/mL (excluding IONPs). Each PFAS initial concentration: 44-118 ppb. Treatment duration: 25 hours. pH= 8.24.


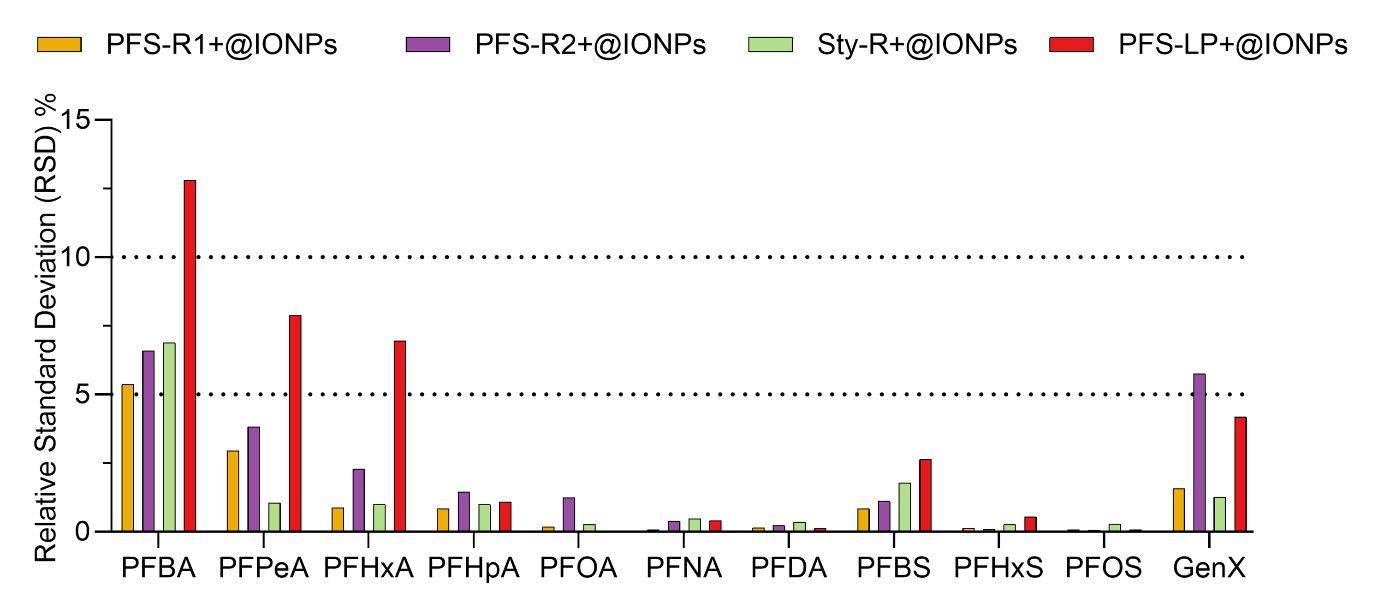
**Figure S13.** Relative standard deviation (RSD) of 11 PFAS removal efficiencies for four magnetic sorbents prepared in three batches. RSD (%)=(standard deviation of removal efficiency across batches/mean removal efficiency across batches) × 100%.


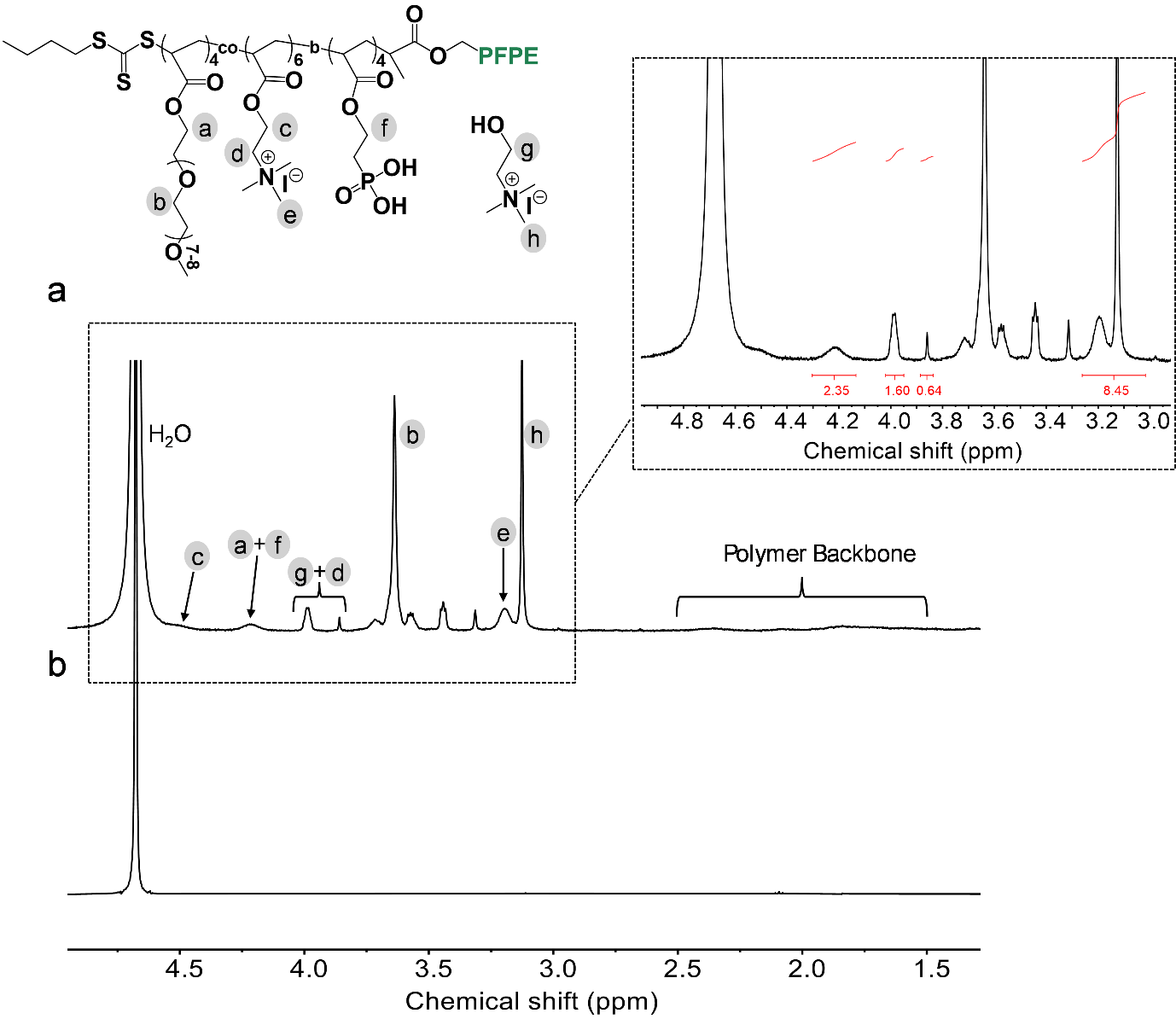


**Figure S14.** ^1^H NMR (in D_2_O) of aqueous solutions containing 0.5 mg/mL sodium phosphate after treating with either PFPE-LP+@IONPs a) or PFS-R1+@IONPs b) for 25 hours and magnetic separation.

**Figure S15.** Removal of 11 different PFAS in landfill leachate by magnetic polymer sorbents and four commercially available sorbents. Sorbent: 8 mg/mL (excluding IONPs) each. Each PFAS initial concentration: 44-118 ppb. Treatment duration: 25 hours. pH= 8.24. The results are the average of three replicates, and one standard deviation is shown.


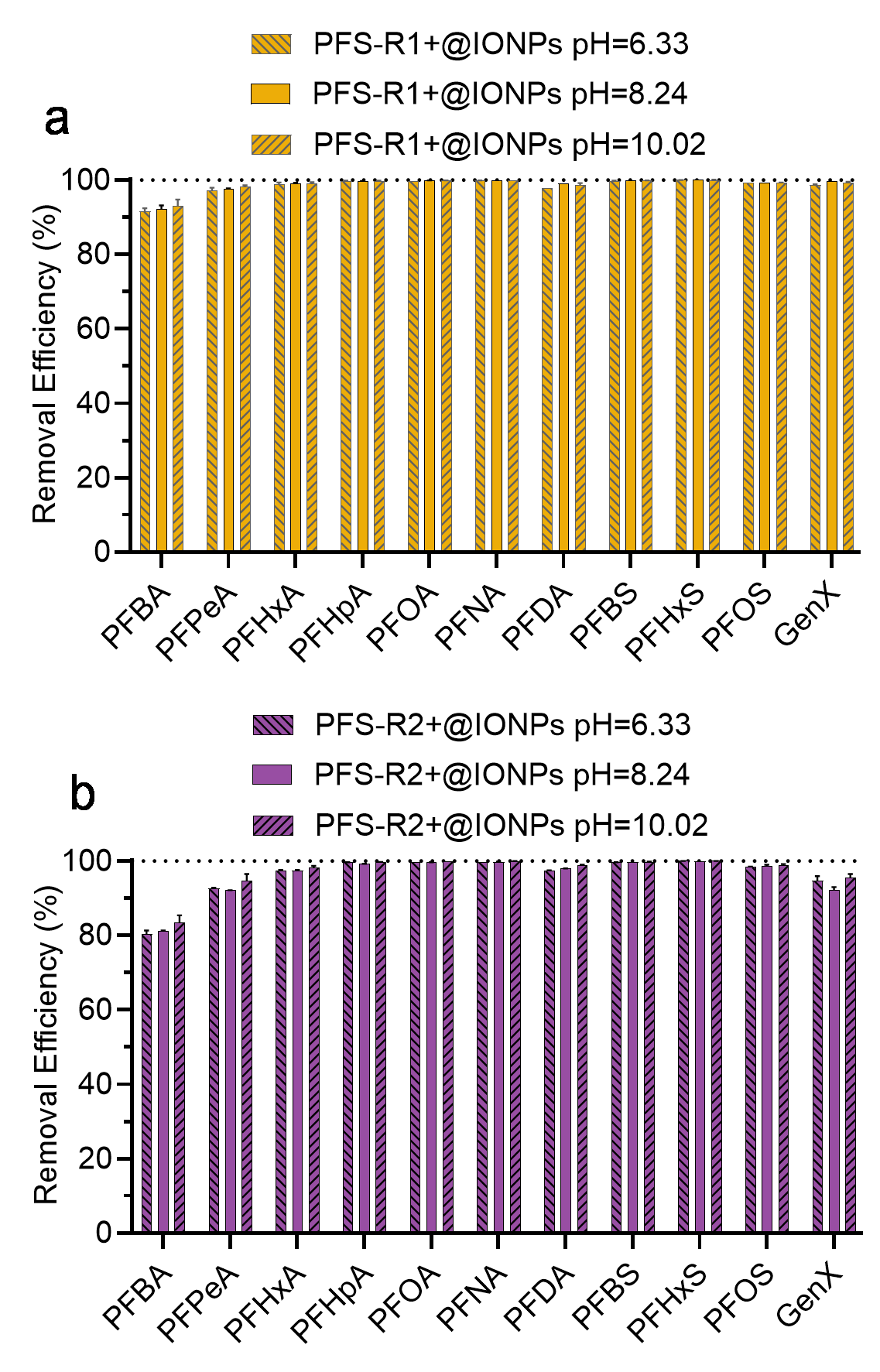


**Figure S16.** Removal of 11 PFAS from landfill leachate using either PFS-R1+@IONPs or PFS-R2+@IONPs at different pH values. Sorbent: 8 mg/mL (excluding IONPs) each. Each PFAS initial concentration: 44-118 ppb. Treatment duration: 25 hours. The results are the average of three replicates, and one standard deviation is shown.


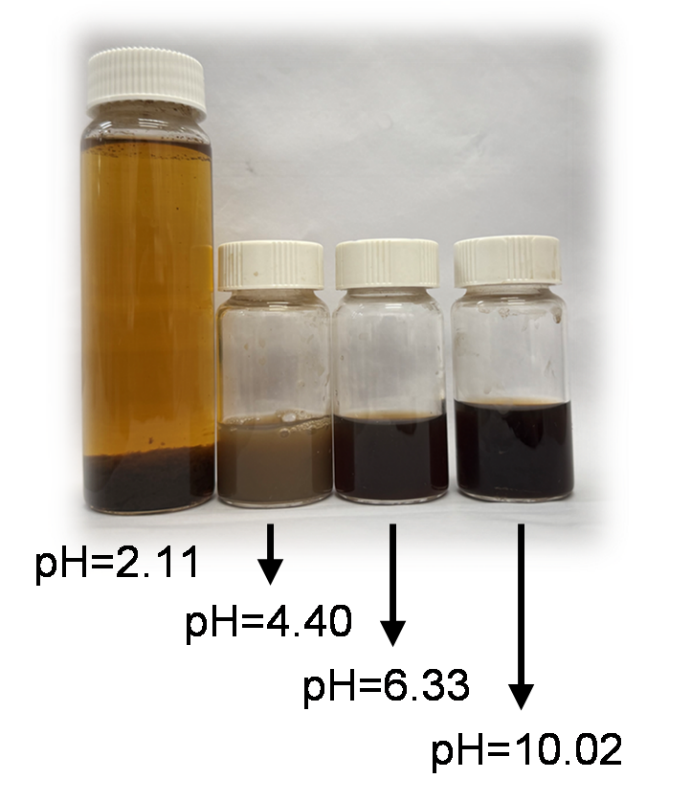


**Figure S17**. Landfill leachate solutions (spiked with 11 PFAS) after pH adjustment (using HCl/NaOH) and before sorbent treatment. Each PFAS initial concentration: 44-118 ppb.





**Figure S18.** Removal efficiency of the 11 PFAS by PFS-R2+@IONPs as a functional of sorption time. Sorbent: 2 mg/mL (excluding IONPs). PFAS initial concentration: 44-118 ppb. pH= 8.24. The results are the average of three replicates, and one standard deviation is shown.





**Figure S19.** Experimental data from kinetics studies for PFS-R1+@IONPs on 11 PFAS, fitted by Pseudo-first-order. Sorbent: 2 mg/mL (excluding IONPs). PFAS initial concentration: 44-118 ppb. pH= 8.24. The results are the average of three replicates, and one standard deviation is shown.

**
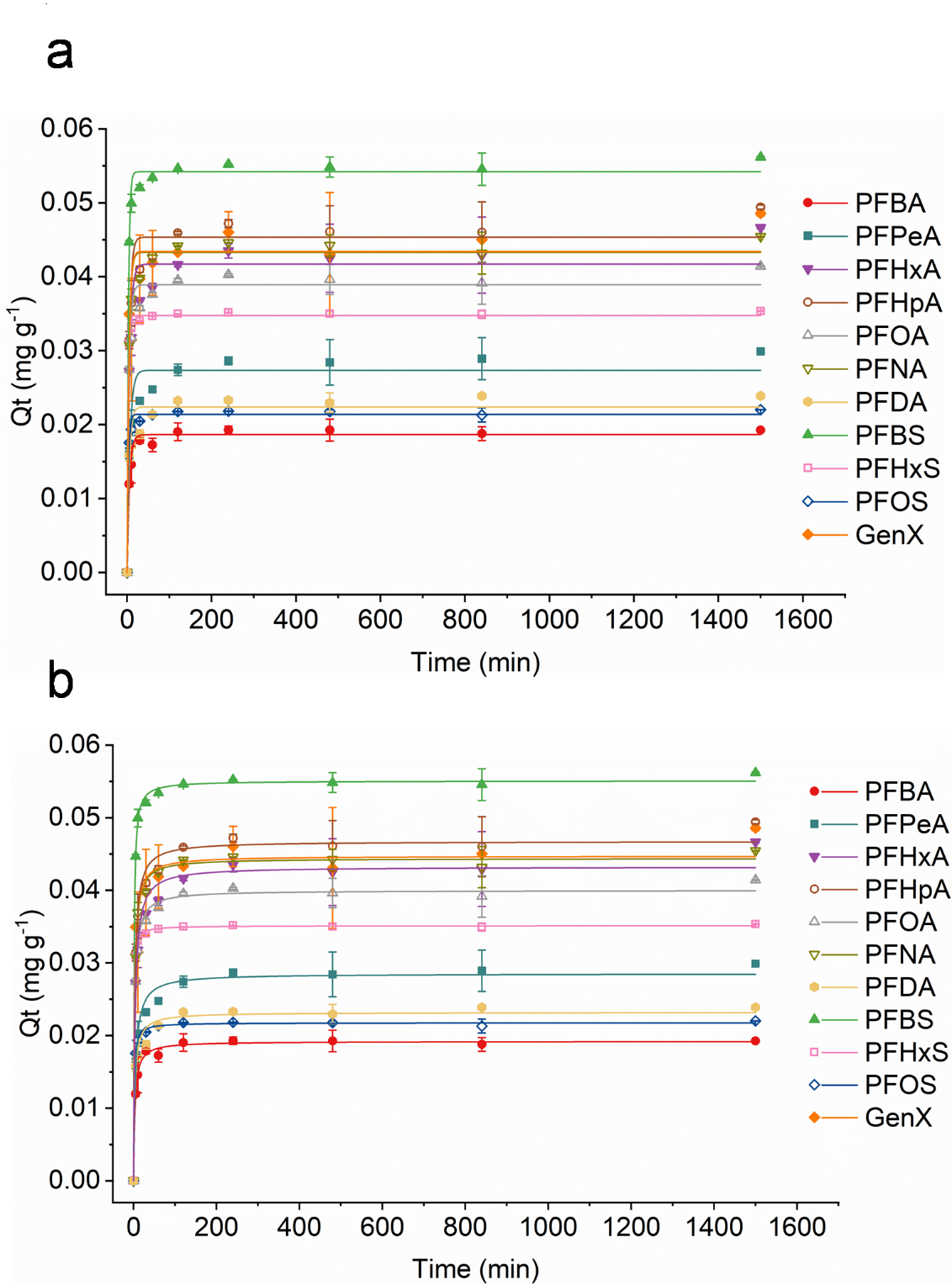
**

**Figure S20.** Experimental data from kinetics studies for PFS-R2+@IONPs on 11 PFAS, fitted by Pseudo-first-order a) and Pseudo-second-order b). Sorbent: 2 mg/mL (excluding IONPs). PFAS initial concentration: 44-118 ppb. pH= 8.24. The results are the average of three replicates, and one standard deviation is shown.

**Table S4.** Parameters achieved from Pseudo-first-order and Pseudo-second-order models for PFS-R1+@IONPs on 11 PFAS.

| PFAS | Pseudo-first-order | | | Pseudo-second-order | | |
| --- | --- | --- | --- | --- | --- | --- |
|  | *k*_1_ | *Q*_e_ | R^2^ | *k*_2_ | *Q*_e_ | R^2^ |
| PFBA | 0.034 | 0.02674 | 0.904 | 1.866 | 0.02844 | 0.966 |
| PFPeA | 0.040 | 0.03504 | 0.915 | 1.692 | 0.03715 | 0.973 |
| PFHxA | 0.106 | 0.04575 | 0.923 | 2.993 | 0.04835 | 0.978 |
| PFHpA | 0.146 | 0.04792 | 0.964 | 4.471 | 0.04995 | 0.994 |
| PFOA | 0.195 | 0.04047 | 0.982 | 8.479 | 0.04167 | 0.998 |
| PFNA | 0.249 | 0.04481 | 0.991 | 11.584 | 0.04579 | 0.9998 |
| PFDA | 0.196 | 0.0232 | 0.945 | 14.760 | 0.02392 | 0.981 |
| PFBS | 0.272 | 0.05526 | 0.990 | 10.770 | 0.05640 | 0.999 |
| PFHxS | 0.376 | 0.03504 | 0.998 | 33.123 | 0.03545 | 0.9996 |
| PFOS | 0.354 | 0.0217 | 0.992 | 43.945 | 0.02203 | 0.999 |
| GenX | 0.131 | 0.04648 | 0.860 | 3.218 | 0.04951 | 0.932 |

**Table S5.** Parameters achieved from Pseudo-first-order and Pseudo-second-order models for PFS-R2+@IONPs on 11 PFAS.

| PFAS | Pseudo-first-order | | | Pseudo-second-order | | |
| --- | --- | --- | --- | --- | --- | --- |
|  | *k*_1_ | *Q*_e_ | R^2^ | *k*_2_ | *Q*_e_ | R^2^ |
| PFBA | 0.181 | 0.01864 | 0.983 | 16.984 | 0.0192 | 0.995 |
| PFPeA | 0.155 | 0.02735 | 0.949 | 8.281 | 0.02852 | 0.986 |
| PFHxA | 0.177 | 0.04171 | 0.954 | 6.738 | 0.04323 | 0.985 |
| PFHpA | 0.205 | 0.04535 | 0.969 | 7.879 | 0.04674 | 0.991 |
| PFOA | 0.214 | 0.03891 | 0.978 | 10.153 | 0.03999 | 0.996 |
| PFNA | 0.229 | 0.0433 | 0.985 | 10.176 | 0.0444 | 0.996 |
| PFDA | 0.197 | 0.02239 | 0.942 | 14.203 | 0.02319 | 0.979 |
| PFBS | 0.330 | 0.0542 | 0.994 | 15.705 | 0.05508 | 0.9987 |
| PFHxS | 0.429 | 0.03476 | 0.998 | 43.046 | 0.03512 | 0.9997 |
| PFOS | 0.320 | 0.0214 | 0.992 | 37.230 | 0.02176 | 0.9987 |
| GenX | 0.236 | 0.04342 | 0.923 | 9.826 | 0.04473 | 0.962 |

**Table S6.** Langmuir and Freundlich constants for the sorption of PFBS using two magnetic polymer sorbents and PFA694E.

|  | **Langmuir Model** | | | **Freundlich Model** | | |
| --- | --- | --- | --- | --- | --- | --- |
| Sorbent Name | *b*  (L mg^-1^) | *Q*_m_  (mg g^-1^) | R^2^ | *K*_F_  ((mg g^-1^)(L mg^-1^)^1/^*^n^*) | *n* | R^2^ |
| PFS-R1+  @IONPs | 0.124 | 128.042 | 0.989 | 16.570 | 1.775 | 0.984 |
| PFA694E | 0.014 | 19.226 | 0.993 | 0.844 | 1.840 | 0.986 |
| PFS-R2+  @IONPs | 0.056 | 263.034 | 0.993 | 14.622 | 1.236 | 0.990 |

**
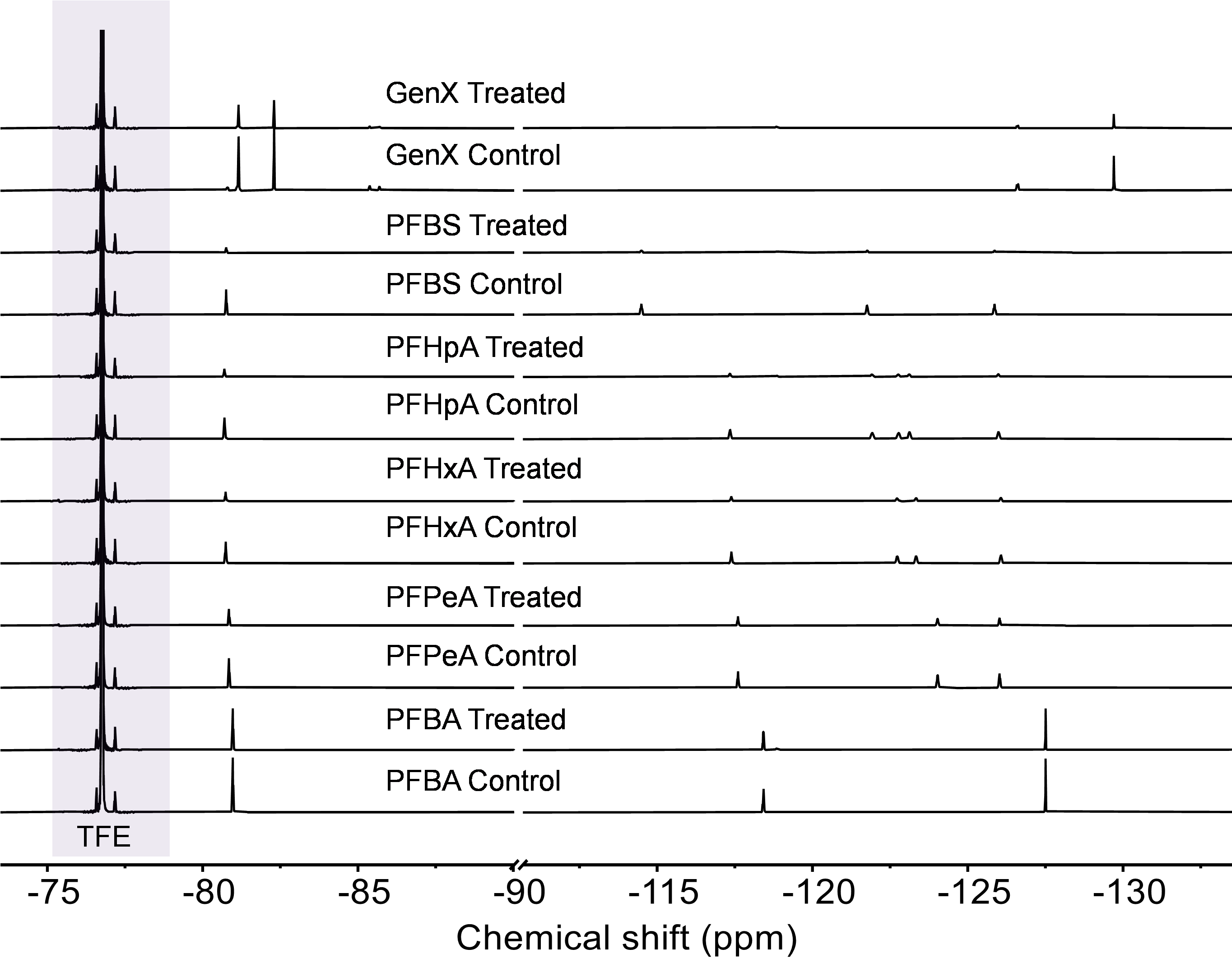
**

**Figure S21.** Stacked ^19^F NMR spectra of five different short-chain PFAS and GenX before and after treatment using PFS-R1+@IONPs. Sorbent: 1 mg/mL (excluding IONPs). PFAS initial concentration: 250 ppm each. Treatment duration: 27 hours. TFE (highlighted in purple) was used as the internal standard for quantifications.

**
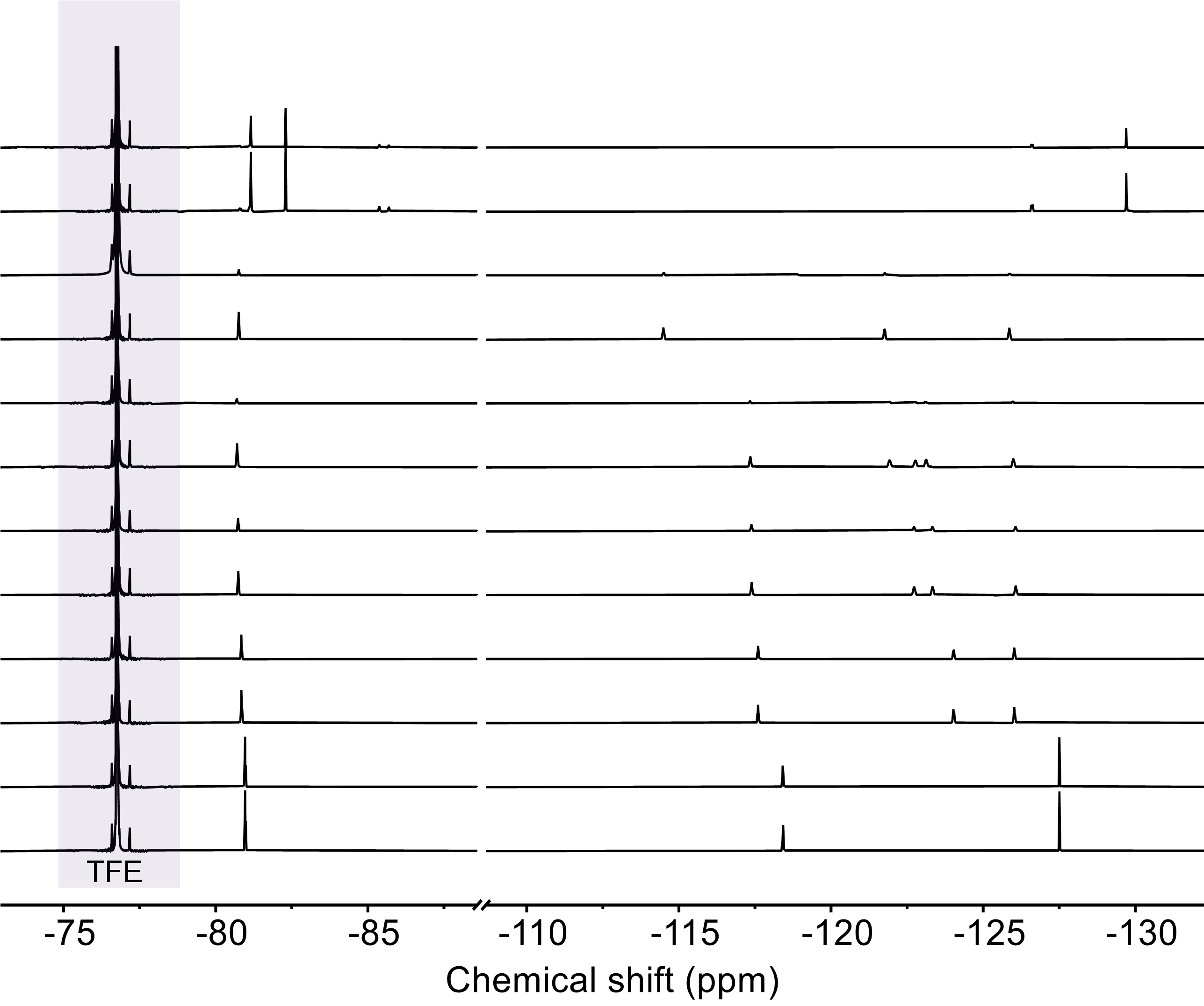
**

**Figure S22.** Stacked ^19^F NMR spectra of five different short-chain PFAS and GenX before and after treatment using PFS-R2+@IONPs. Sorbent: 1 mg/mL (excluding IONPs). PFAS initial concentration: 250 ppm each. Treatment duration: 27 hours. TFE (highlighted in purple) was used as the internal standard for quantifications.

**Table S7.** Compositions of original drinking water before PFAS spiking.

|  | Compositions | Concentration (mg/L) |
| --- | --- | --- |
| Total Carbon | Total organic carbon (TOC) | 0.0 |
|  | Total inorganic carbon (TIC) | 59.27 |
|  | Total carbon (TC) | 47.05 |
| Element | Al | 0.001 |
|  | As | 0.025 |
|  | B | 0.137 |
|  | Ba | 0.068 |
|  | Ca | 33.2 |
|  | Cd | n.d. |
|  | Co | 0.001 |
|  | Cr | 0.006 |
|  | Cu | 0.192 |
|  | Fe | 0.005 |
|  | K | 3.6 |
|  | Mg | 13.9 |
|  | Mn | 0.003 |
|  | Mo | n.d. |
|  | Na | 32.7 |
|  | Ni | 0.009 |
|  | P | 0.005 |
|  | Pb | n.d. |
|  | S | 11.8 |
|  | Se | n.d. |
|  | Si | 4.191 |
|  | Sr | 0.194 |
|  | V | n.d. |
|  | Zn | 0.058 |

Note: The concentration of each element was measured using inductively coupled plasma optical emission spectroscopy (ICP-OES). n.d.: element not tested.

**Table S8.** Compositions of original compost leachate before PFAS spiking.

|  | Compositions | Concentration (mg/L) |
| --- | --- | --- |
| Total Carbon | Total organic carbon (TOC) | 1.62 |
|  | Total inorganic carbon (TIC) | 0.37 |
|  | Total carbon (TC) | 1.99 |
| Element | Al | 0.04 |
|  | As | 0.012 |
|  | B | 0.416 |
|  | Ba | 0.097 |
|  | Ca | 25.2 |
|  | Cd | 0.001 |
|  | Co | 0.001 |
|  | Cr | 0.001 |
|  | Cu | 0.024 |
|  | Fe | 0.626 |
|  | K | 68.3 |
|  | Mg | 25.5 |
|  | Mn | 0.002 |
|  | Mo | 0.01 |
|  | Na | 70.0 |
|  | Ni | 0.01 |
|  | P | 2.79 |
|  | Pb | 0.001 |
|  | S | 45.1 |
|  | Se | 0.002 |
|  | Si | 4.18 |
|  | Sr | 0.073 |
|  | V | 0.006 |
|  | Zn | 0.023 |

Note: The concentration of each element was measured using inductively coupled plasma optical emission spectroscopy (ICP-OES).

**
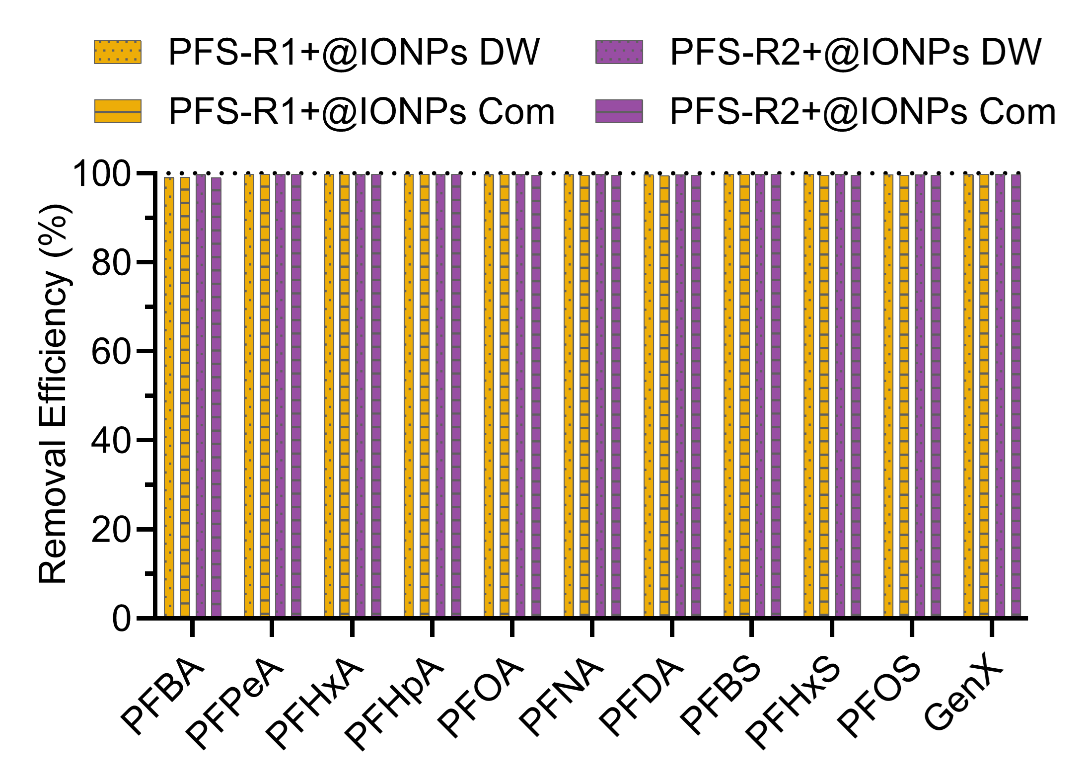
**

**Figure S23.** Removal of 11 PFAS from two different water matrices at environmentally relevant concentrations, including drinking water and compost leachate, using either PFS-R1+@IONPs or PFS-R2+@IONPs. Sorbent: 4 mg/mL (excluding IONPs); PFAS initial concentration: ~1 ppb each; Treatment duration: 2 h.

**Table S9.** Pairwise force field coefficients and partial charge for PFAS molecule.

| Atom | ε (kcal/mol) | σ (Å) | Partial Charge (e) |
| --- | --- | --- | --- |
| C1 | 0.066 | 3.5 | 0.356 |
| C2 | 0.066 | 3.5 | 0.111 |
| C3 | 0.066 | 3.5 | 0.2132 |
| C4 | 0.066 | 3.5 | -0.0031 |
| S5 | 0.25 | 3.55 | 0.7464 |
| O6 | 0.17 | 2.96 | -0.5186 |
| O7, O8 | 0.17 | 2.96 | -0.41 |
| F9 | 0.053 | 2.95 | 0.0041 |
| F10 | 0.053 | 2.95 | -0.1483 |
| F11 | 0.053 | 2.95 | -0.1488 |
| F12 | 0.053 | 2.95 | -0.119 |
| F13 | 0.053 | 2.95 | -0.1189 |
| F14 | 0.053 | 2.95 | -0.1334 |
| F15 | 0.053 | 2.95 | -0.134 |
| F16 | 0.053 | 2.95 | -0.0703 |
| F17 | 0.053 | 2.95 | -0.0698 |

**Table S10.** Pairwise force field coefficients and partial charge for PFS molecule.

| Atom | ε (kcal/mol) | σ (Å) | Partial Charge (e) |
| --- | --- | --- | --- |
| C1 | 0.066 | 3.5 | -0.1815 |
| H2 | 0.03 | 2.5 | 0.0662 |
| C3 | 0.066 | 3.5 | 0.1248 |
| H4 | 0.03 | 2.5 | 0.0687 |
| C5 | 0.07 | 3.55 | -0.0797 |
| H6 | 0.03 | 2.5 | 0.025 |
| C7 | 0.07 | 3.55 | 0.1583 |
| C8 | 0.07 | 3.55 | 0.1547 |
| C9 | 0.07 | 3.55 | 0.0671 |
| F10 | 0.061 | 2.85 | -0.1647 |
| C11 | 0.07 | 3.55 | 0.064 |
| F12 | 0.061 | 2.85 | -0.1614 |
| C13 | 0.07 | 3.55 | 0.2076 |
| F14 | 0.061 | 2.85 | -0.1421 |
| F15 | 0.061 | 2.85 | -0.1406 |
| F16 | 0.061 | 2.85 | -0.1588 |
| H17 | 0.03 | 2.5 | 0.0677 |
| H18 | 0.03 | 2.5 | 0.0247 |

**Table S11.** Pairwise force field coefficients and partial charge for VBTAC molecule.

| Atom | ε (kcal/mol) | σ (Å) | Partial Charge (e) |
| --- | --- | --- | --- |
| C1 | 0.066 | 3.5 | -1.21217 |
| H2 | 0.03 | 2.5 | -1.19457 |
| C3 | 0.066 | 3.5 | -2.36613 |
| H4 | 0.03 | 2.5 | -1.3641 |
| C5 | 0.07 | 3.55 | -2.15304 |
| H6 | 0.03 | 2.5 | -3.35559 |
| H7 | 0.03 | 2.5 | -2.38924 |
| C8 | 0.07 | 3.55 | -0.9242 |
| C9 | 0.07 | 3.55 | -3.17693 |
| C10 | 0.07 | 3.55 | -0.7225 |
| H11 | 0.03 | 2.42 | -0.16579 |
| C12 | 0.07 | 3.55 | -2.97192 |
| H13 | 0.03 | 2.42 | -4.08717 |
| C14 | 0.07 | 3.55 | -1.7459 |
| H15 | 0.03 | 2.42 | 0.182182 |
| H16 | 0.03 | 2.42 | -3.72987 |
| C17 | 0.066 | 3.5 | -1.5645 |
| H18 | 0.03 | 2.5 | -2.49217 |
| H19 | 0.03 | 2.5 | -1.46021 |
| N20 | 0.17 | 3.25 | -0.35042 |
| C21 | 0.066 | 3.5 | -0.46741 |
| C22 | 0.066 | 3.5 | -0.27172 |
| C23 | 0.066 | 3.5 | 0.908008 |
| H24 | 0.03 | 2.5 | -0.51005 |
| H25 | 0.03 | 2.5 | -1.41681 |
| H26 | 0.03 | 2.5 | 0.438834 |
| H27 | 0.03 | 2.5 | 0.635516 |
| H28 | 0.03 | 2.5 | -1.21867 |
| H29 | 0.03 | 2.5 | -0.17175 |
| H30 | 0.03 | 2.5 | 0.990107 |
| H31 | 0.03 | 2.5 | 0.882132 |
| H32 | 0.03 | 2.5 | 1.81129 |
| H33 | 0.03 | 2.5 | -0.22368 |

**Table S12.** Pairwise force field coefficients and partial charge for HA molecule.

| Atom | σ (Å) | ε (kcal/mol) | Partial Charge (e) |
| --- | --- | --- | --- |
| C1 | 3.5 | 0.065998416 | -0.0658 |
| H2 | 2.42 | 0.01499964 | 0.1381 |
| C3 | 3.75 | 0.10499748 | -0.0936 |
| H4 | 2.42 | 0.01499964 | 0.1867 |
| O5 | 2.96 | 0.20999496 | -0.4079 |
| O6 | 3.12 | 0.16999592 | -0.521 |
| H7 | 0 | 0 | 0.2998 |
| C8 | 3.75 | 0.10499748 | 0.2875 |
| O9 | 2.96 | 0.20999496 | -0.5751 |
| C10 | 3.55 | 0.06999832 | 0.0322 |
| C11 | 3.55 | 0.06999832 | -0.3246 |
| C12 | 3.55 | 0.06999832 | -0.3198 |
| C13 | 3.55 | 0.06999832 | 0.4144 |
| H14 | 2.42 | 0.02999928 | 0.1723 |
| C15 | 3.55 | 0.06999832 | -0.401 |
| H16 | 2.42 | 0.02999928 | 0.2107 |
| C17 | 3.55 | 0.06999832 | 0.0798 |
| H18 | 2.42 | 0.02999928 | 0.1963 |
| N19 | 3.25 | 0.16999592 | -0.9025 |
| H20 | 0 | 0 | 0.424 |
| H21 | 0 | 0 | 0.3962 |
| O22 | 2.9 | 0.13999664 | -0.444 |
| C23 | 3.5 | 0.065998416 | 0.2492 |
| C24 | 3.5 | 0.065998416 | 0.2263 |
| H25 | 2.5 | 0.02999928 | -0.0103 |
| H26 | 2.5 | 0.02999928 | 0.0194 |
| C27 | 3.5 | 0.065998416 | -0.3012 |
| H28 | 2.5 | 0.02999928 | 0.0691 |
| H29 | 2.5 | 0.02999928 | 0.1223 |
| O30 | 3.07 | 0.16999592 | -0.4918 |
| H31 | 0 | 0 | 0.3375 |
| C32 | 3.55 | 0.075998176 | -0.3521 |
| C33 | 3.55 | 0.075998176 | -0.1366 |
| C34 | 3.75 | 0.10499748 | 0.3398 |
| C35 | 3.55 | 0.075998176 | -0.1267 |
| H36 | 2.42 | 0.02999928 | 0.1304 |
| C37 | 3.5 | 0.065998416 | -0.0315 |
| C38 | 3.55 | 0.075998176 | -0.1233 |
| H39 | 2.5 | 0.02999928 | 0.0931 |
| H40 | 2.42 | 0.02999928 | 0.0865 |
| C41 | 3.5 | 0.065998416 | -0.0112 |
| H42 | 2.5 | 0.02999928 | 0.058 |
| C43 | 3.5 | 0.065998416 | 0.0966 |
| H44 | 2.5 | 0.02999928 | 0.0806 |
| H45 | 2.5 | 0.02999928 | 0.1333 |
| C46 | 3.75 | 0.10499748 | 0.3792 |
| O47 | 2.96 | 0.20999496 | -0.3557 |
| O48 | 2.96 | 0.20999496 | -0.2384 |
| C49 | 3.55 | 0.075998176 | -0.4884 |
| H50 | 2.42 | 0.02999928 | 0.1908 |
| C51 | 3.55 | 0.075998176 | -0.3059 |
| H52 | 2.42 | 0.02999928 | 0.2546 |
| C53 | 3.75 | 0.10499748 | 0.3848 |
| O54 | 2.96 | 0.20999496 | -0.2998 |
| O55 | 2.96 | 0.20999496 | -0.3192 |
| O56 | 2.96 | 0.20999496 | -0.4924 |
| H57 | 2.5 | 0.02999928 | 0.0742 |
| C58 | 3.5 | 0.065998416 | -0.0879 |
| H59 | 2.5 | 0.02999928 | 0.0856 |
| C60 | 3.5 | 0.065998416 | 0.2073 |
| H61 | 2.5 | 0.02999928 | 0.037 |
| C62 | 3.5 | 0.065998416 | 0.0381 |
| H63 | 2.5 | 0.02999928 | 0.0506 |
| H64 | 2.5 | 0.02999928 | 0.0475 |
| O65 | 3.12 | 0.16999592 | -0.4567 |
| H66 | 0 | 0 | 0.3076 |
| C67 | 3.75 | 0.10499748 | 0.0999 |
| O68 | 2.96 | 0.20999496 | -0.3847 |
| O69 | 2.96 | 0.20999496 | -0.4059 |
| C70 | 3.5 | 0.065998416 | -0.302 |
| H71 | 2.5 | 0.02999928 | 0.1053 |
| O72 | 3.12 | 0.16999592 | -0.3819 |
| H73 | 0 | 0 | 0.2726 |
| C74 | 3.55 | 0.06999832 | -0.0873 |
| C75 | 3.55 | 0.06999832 | 0.1117 |
| C76 | 3.55 | 0.06999832 | -0.0405 |
| C77 | 3.55 | 0.06999832 | 0.1148 |
| C78 | 3.55 | 0.06999832 | -0.1411 |
| H79 | 2.42 | 0.02999928 | 0.1178 |
| C80 | 3.55 | 0.06999832 | 0.2216 |
| H81 | 2.42 | 0.02999928 | 0.0892 |
| N82 | 3.25 | 0.16999592 | -0.7599 |
| H83 | 0 | 0 | 0.2636 |
| H84 | 0 | 0 | 0.3635 |
| O85 | 3.07 | 0.16999592 | -0.5993 |
| H86 | 0 | 0 | 0.3353 |
| O87 | 3.07 | 0.16999592 | -0.5621 |
| H88 | 0 | 0 | 0.3164 |


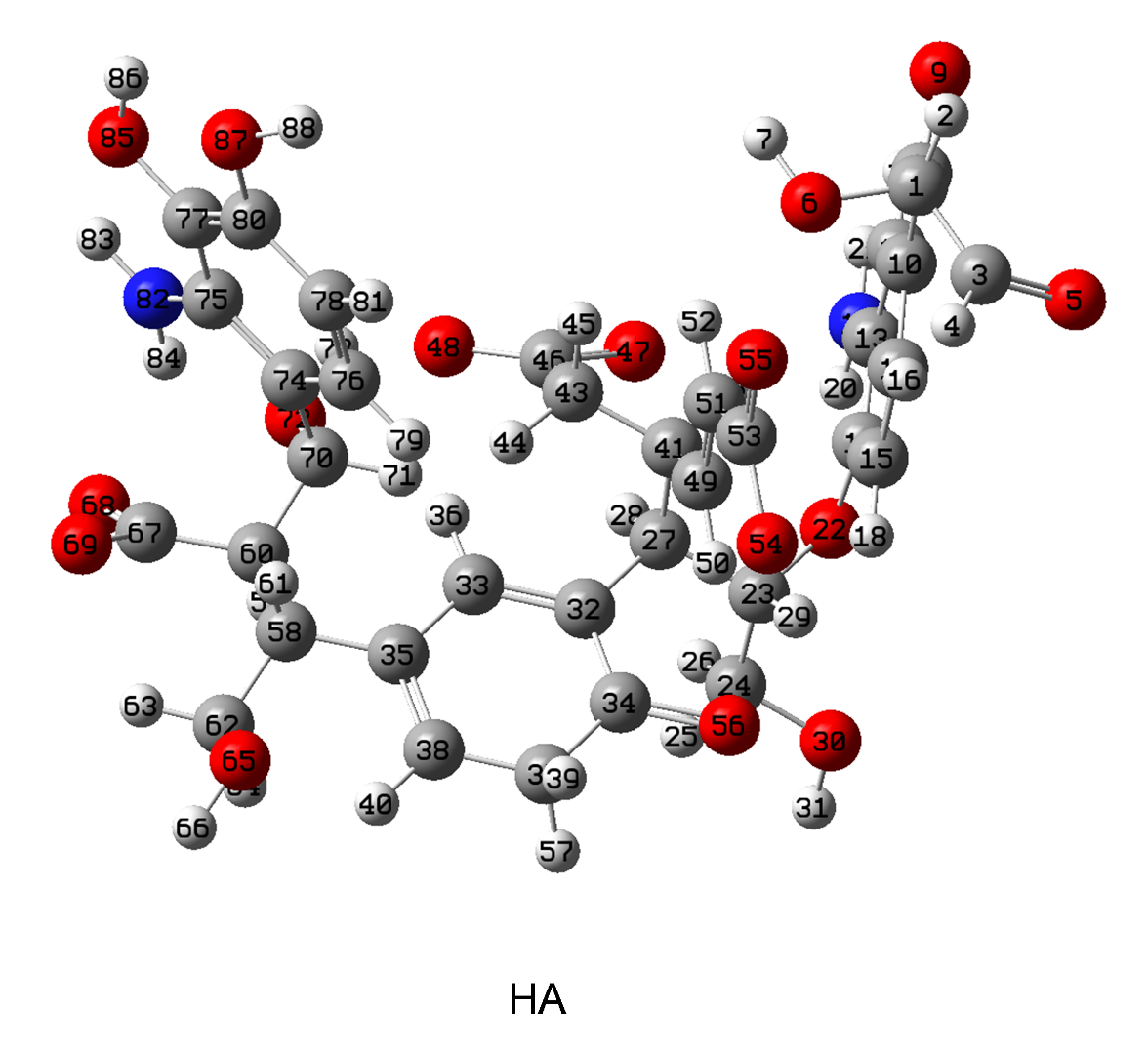


**Figure S24.** Molecular structure of HA modelled by the Temple-Northeastern-Birmingham (TNB) approach.

**
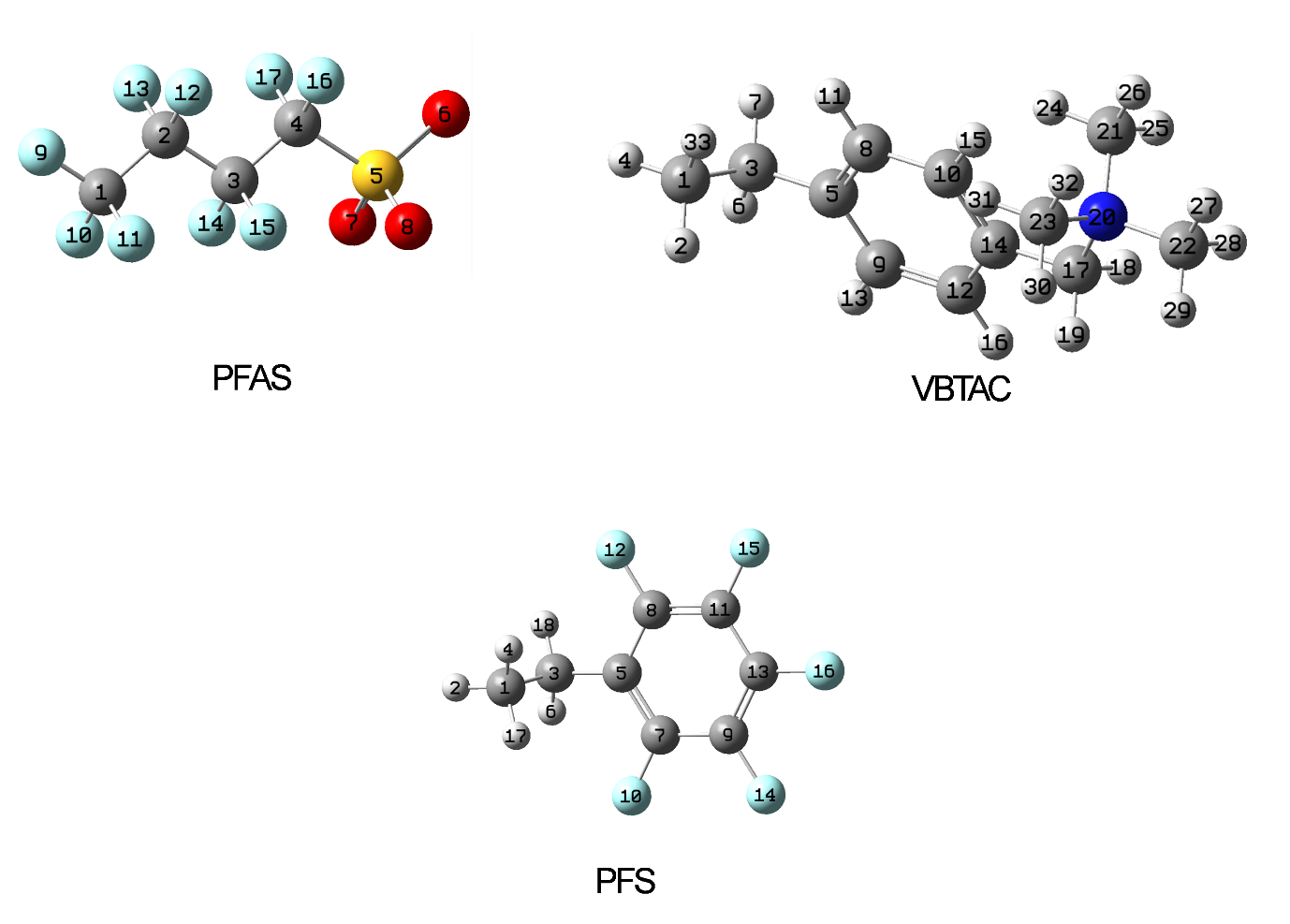
**

**Figure S25.** Chemical structures of PFAS and functional polymer units including VBTAC and PFS.

**
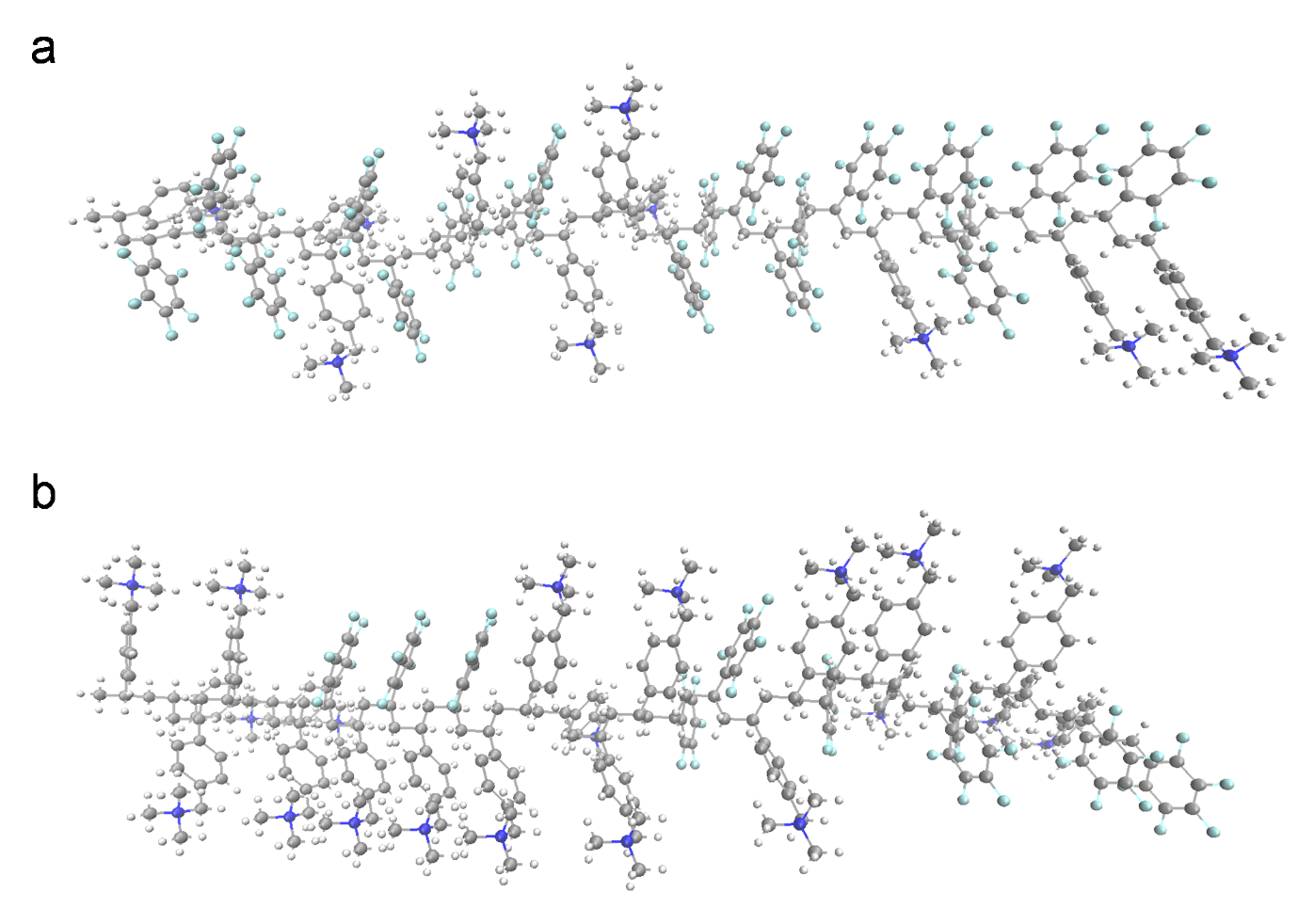
Figure S26.** Initial structures of the simplified PFS-R1+ a) and PFS-R2+ b).

**
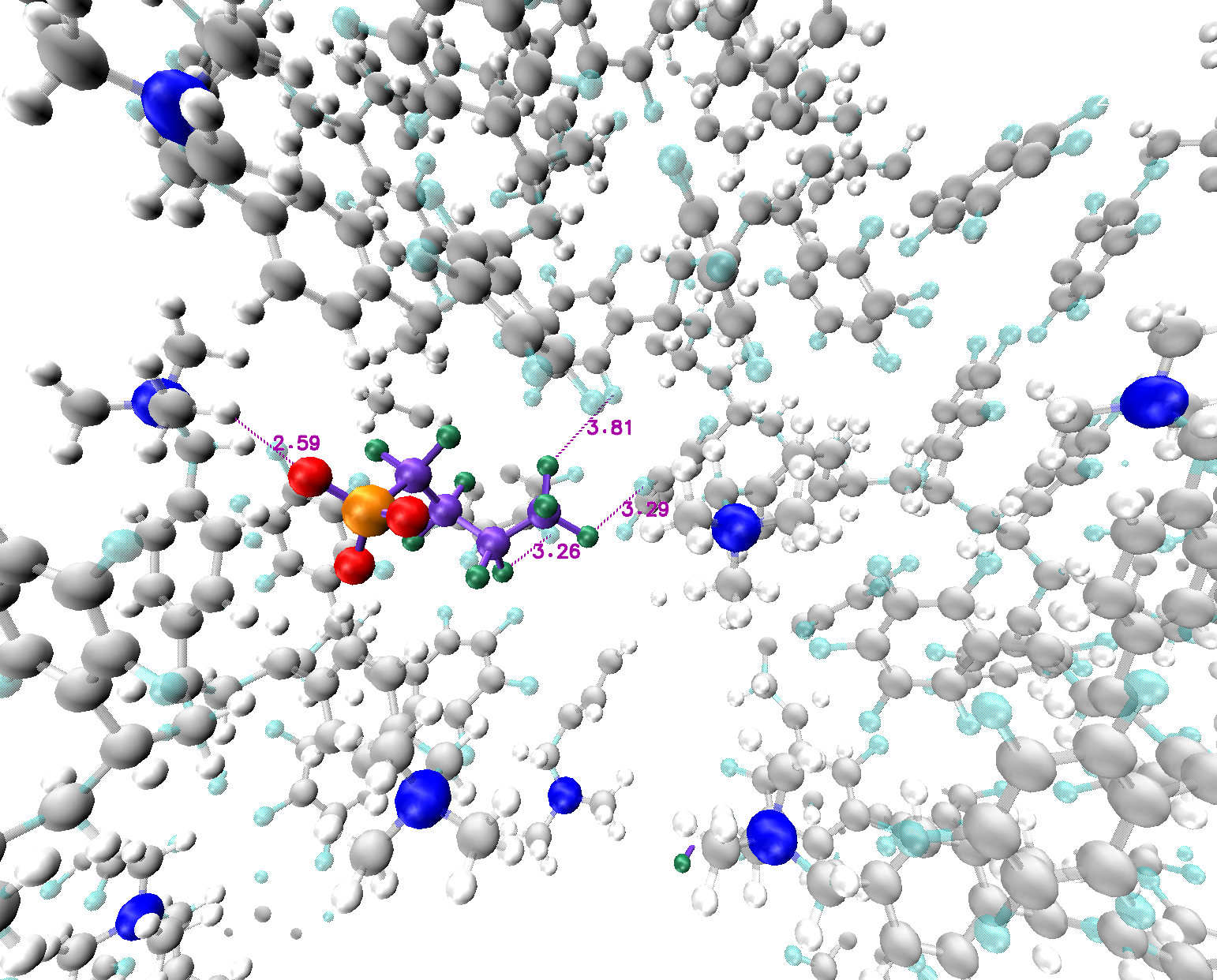
**

**Figure S27.** Snapshot of PFBS interacting with PFS-R1+ at 11 ns (Cl atom hidden for clarity), showing a 2.59 Å distance between an ammonium methyl group and a sulfonate oxygen of PFBS.

**
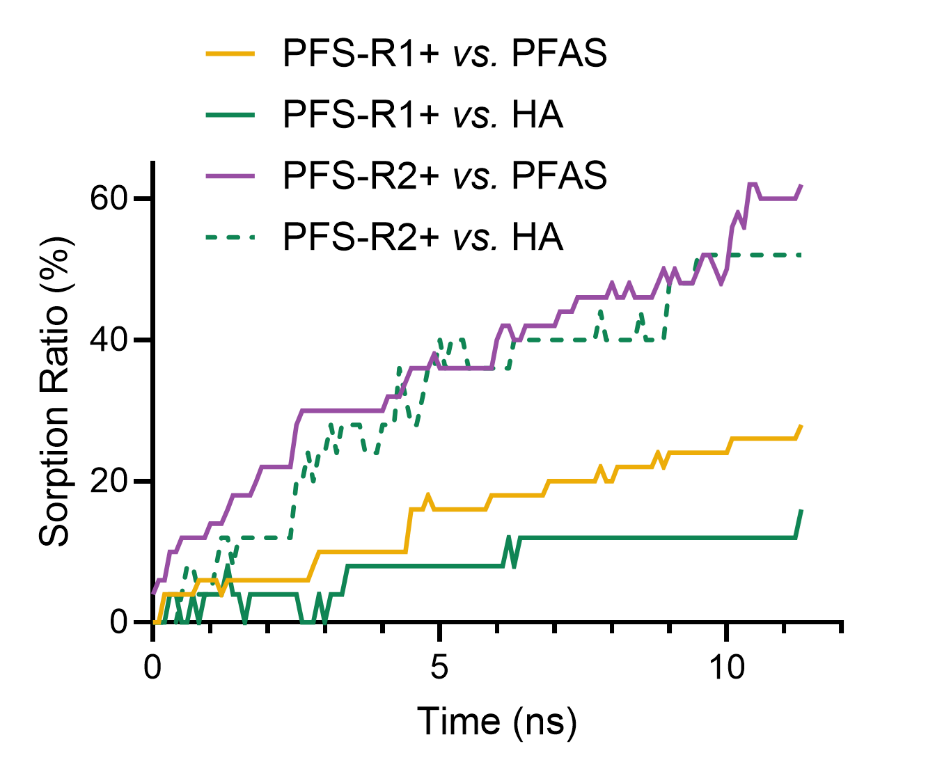
**

**Figure S28.** Sorption ratios of PFBS and HA by PFS-R1+ and PFS-R2+ over simulation time, obtained from molecular dynamics simulations.

**
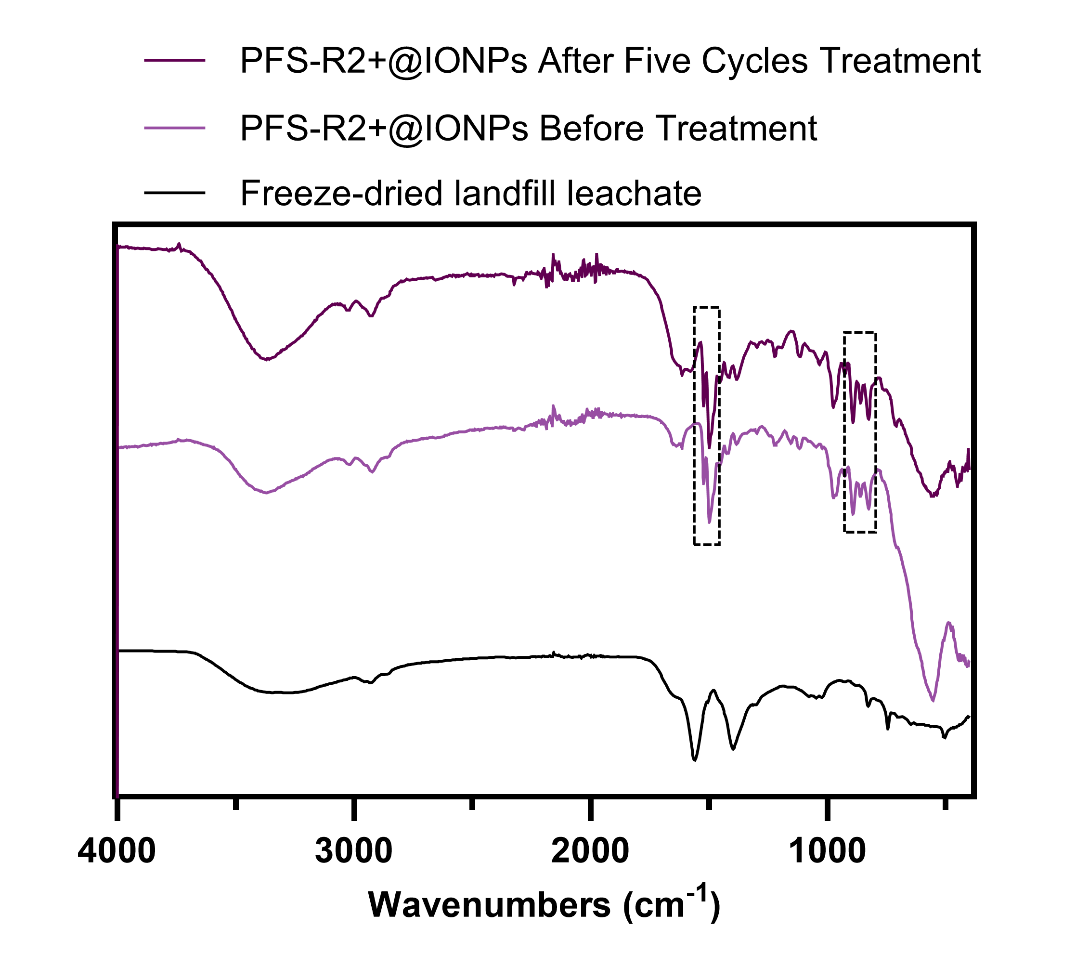
**

**Figure S29.** FTIR stacked spectra of PFS-R2+@IONPs before treatment, freeze-dried PFS-R2+@IONPs after five cycles of sorption and desorption treatment, and freeze-dried landfill leachate.

**Table S13.** Multiple reaction monitoring details for analytes shown in bold font, and internal standards used are shown in italic font.

| Compound ID | Q1 mass | Q3 mass | EP | CE | CXP | Retention time |
| --- | --- | --- | --- | --- | --- | --- |
| **PFBA 1** | 212.8 | 169.0 | -8 | -13 | -18 | 1.4 |
| **PFPeA 1** | 262.8 | 219.0 | -8 | -12 | -25 | 1.73 |
| **PFPeA 2** | 262.8 | 69.0 | -8 | -58 | -8 | 1.73 |
| **PFBS 1** | 298.9 | 80.0 | -10 | -65 | -12 | 1.77 |
| **PFBS 2** | 298.9 | 99.0 | -10 | -36 | -9 | 1.77 |
| **PFHxA 1** | 312.8 | 269.0 | -8 | -12 | -25 | 2.02 |
| **PFHxA 2** | 312.8 | 119.0 | -8 | -24 | -12 | 2.02 |
| **GenX 1** | 328.9 | 185.0 | -10 | -32 | -16 | 2.14 |
| **GenX 2** | 328.9 | 119.0 | -10 | -49 | -10 | 2.14 |
| **PFHpA 1** | 362.8 | 319.0 | -8 | -14 | -25 | 2.39 |
| **PFHpA 2** | 362.8 | 169.0 | -8 | -23 | -18 | 2.39 |
| **PFHxS 1** | 398.8 | 80.0 | -10 | -90 | -12 | 2.4 |
| **PFHxS 2** | 398.8 | 99.0 | -10 | -75 | -9 | 2.4 |
| **PFOA 1** | 412.8 | 369.0 | -9 | -14 | -15 | 2.81 |
| **PFOA 2** | 412.8 | 169.0 | -9 | -23 | -15 | 2.81 |
| **PFNA 1** | 462.8 | 419.0 | -9 | -15 | -35 | 3.27 |
| **PFNA 2** | 462.8 | 169.0 | -9 | -26 | -18 | 3.27 |
| **PFOS 1** | 498.8 | 80.0 | -10 | -100 | -11 | 3.27 |
| **PFOS 2** | 498.8 | 99.0 | -10 | -95 | -13 | 3.27 |
| **PFDA 1** | 512.8 | 469.0 | -10 | -18 | -35 | 3.73 |
| **PFDA 2** | 512.8 | 269.0 | -10 | -23 | -25 | 3.73 |
| *^13^C_4_-PFBA* | 216.8 | 172.0 | -8 | -12 | -14 | 1.39 |
| *^13^C_4_-PFPeA* | 265.9 | 222.0 | -9 | -13 | -20 | 1.72 |
| *^13^C_3_-PFBS* | 301.8 | 80.0 | -10 | -70 | -12 | 1.76 |
| *^13^C_2-_PFHxA* | 314.9 | 270.0 | -8 | -13 | -25 | 2.01 |
| *^13^C_3_-GenX* | 332 | 185.0 | -10 | -32 | -15 | 2.18 |
| *^13^C_3_-PFHpA* | 366.8 | 322.0 | -9 | -14 | -28 | 2.37 |
| *^18^O_2-_PFHxS* | 402.8 | 103.0 | -8 | -80 | -10 | 2.39 |
| *^13^C_4_-PFOA* | 416.9 | 372.0 | -10 | -13 | -28 | 2.81 |
| *^13^C_5_-PFNA* | 467.8 | 423.0 | -9 | -14 | -30 | 3.27 |
| *^13^C_4_-PFOS* | 502.9 | 80.0 | -9 | -115 | -10 | 3.27 |
| *^13^C_2-_PFDA* | 514.8 | 470.0 | -10 | -15 | -35 | 3.72 |

EP=Entrance potential, CE=Collision energy, CXP=Collision exit potential

**References**

(1) Plimpton, S. Fast Parallel Algorithms for Short-Range Molecular Dynamics. *J. Comput. Phys.* **1995,** *117*, 1-19.

(2) Martínez, L.; Andrade, R.; Birgin, E. G.; Martínez, J. M. PACKMOL: A package for building initial configurations for molecular dynamics simulations. *J. Comput. Chem.* **2009,** *30*, 2157-2164.

(3) Davies, G.; Fataftah, A.; Cherkasskiy, A.; Ghabbour, E. A.; Radwan, A.; Jansen, S. A.; Kolla, S.; Paciolla, M. D.; Sein, L. T.; Buermann, W.; Balasubramanian, M.; Budnick, J.; Xing, B. S. Tight metal binding by humic acids and its role in biomineralization. *J. Chem. Soc., Dalton Trans.* **1997,** 4047-4060.

(4) Sutton, R.; Sposito, G. Molecular Structure in Soil Humic Substances:  The New View. *Environ. Sci. Technol.* **2005,** *39*, 9009-9015.

(5) Jorgensen, W. L.; Maxwell, D. S.; TiradoRives, J. Development and Testing of the OPLS All-Atom Force Field on Conformational Energetics and Properties of Organic Liquids. *J. Am. Chem. Soc.* **1996,** *118*, 11225-11236.

(6) Mark, P.; Nilsson, L. Structure and Dynamics of the TIP3P, SPC, and SPC/E Water Models at 298 K. *J. Phys. Chem. A* **2001,** *105*, 9954-9960.

(7) Bayly, C. I.; Cieplak, P.; Cornell, W. D.; Kollman, P. A. A Well-Behaved Electrostatic Potential Based Method Using Charge Restraints for Deriving Atomic Charges: The RESP Model. *J. Phys. Chem.* **1993,** *97*, 10269-10280.

(8) Marenich, A. V.; Cramer, C. J.; Truhlar, D. G. Universal Solvation Model Based on Solute Electron Density and on a Continuum Model of the Solvent Defined by the Bulk Dielectric Constant and Atomic Surface Tensions. *J. Phys. Chem. B* **2009,** *113*, 6378-6396.

(9) Zhao, Y.; Truhlar, D. G. The M06 suite of density functionals for main group thermochemistry, thermochemical kinetics, noncovalent interactions, excited states, and transition elements: two new functionals and systematic testing of four M06-class functionals and 12 other functionals. *Theor. Chem. Acc.* **2008,** *120*, 215-241.

(10) Weigend, F.; Ahlrichs, R. Balanced basis sets of split valence, triple zeta valence and quadruple zeta valence quality for H to Rn: Design and assessment of accuracy. *Phys. Chem. Chem. Phys.* **2005,** *7*, 3297-3305.

(11) Frisch, M. J.; Trucks, G. W.; Schlegel, H. B.; Scuseria, G. E.; Robb, M. A.; Cheeseman, J. R.; Scalmani, G.; Barone, V.; Petersson, G. A.; Nakatsuji, H.; Li, X.; Caricato, M.; Marenich, A. V.; Bloino, J.; Janesko, B. G.; Gomperts, R.; Mennucci, B.; Hratchian, H. P.; Ortiz, J. V.; Izmaylov, A. F.; Sonnenberg, J. L.; Williams; Ding, F.; Lipparini, F.; Egidi, F.; Goings, J.; Peng, B.; Petrone, A.; Henderson, T.; Ranasinghe, D.; Zakrzewski, V. G.; Gao, J.; Rega, N.; Zheng, G.; Liang, W.; Hada, M.; Ehara, M.; Toyota, K.; Fukuda, R.; Hasegawa, J.; Ishida, M.; Nakajima, T.; Honda, Y.; Kitao, O.; Nakai, H.; Vreven, T.; Throssell, K.; Montgomery Jr., J. A.; Peralta, J. E.; Ogliaro, F.; Bearpark, M. J.; Heyd, J. J.; Brothers, E. N.; Kudin, K. N.; Staroverov, V. N.; Keith, T. A.; Kobayashi, R.; Normand, J.; Raghavachari, K.; Rendell, A. P.; Burant, J. C.; Iyengar, S. S.; Tomasi, J.; Cossi, M.; Millam, J. M.; Klene, M.; Adamo, C.; Cammi, R.; Ochterski, J. W.; Martin, R. L.; Morokuma, K.; Farkas, O.; Foresman, J. B.; Fox, D. J. *Gaussian 16 Rev. A.03*, Wallingford, CT, 2016.

(12) Jewett, A. I.; Stelter, D.; Lambert, J.; Saladi, S. M.; Roscioni, O. M.; Ricci, M.; Autin, L.; Maritan, M.; Bashusqeh, S. M.; Keyes, T.; Dame, R. T.; Shea, J. E.; Jensen, G. J.; Goodsell, D. S. Moltemplate: A Tool for Coarse-Grained Modeling of Complex Biological Matter and Soft Condensed Matter Physics. *J. Mol. Biol.* **2021,** *433*, 166841.

(13) Zhang, T. Y.; Xing, Y. H.; Zhang, J. G.; Li, X. The competition of humic acid aggregation and adsorption on clay particles and its role in retarding heavy metal ions. *Sci. Total Environ.* **2024,** *954*, 176459.

(14) Barbosa, G. D.; Turner, C. H. Investigating the molecular-level thermodynamics and adsorption properties of per- and poly-fluoroalkyl substances. *J. Mol. Liq.* **2023,** *389*, 122826.

(15) Humphrey, W.; Dalke, A.; Schulten, K. VMD: Visual Molecular Dynamics. *J. Mol. Graph.* **1996,** *14*, 33-38.
